# Solid-Phase Synthesis of Gly-Ψ[CH(CF_3_)NH]-Peptides

**DOI:** 10.1021/acs.joc.1c00853

**Published:** 2021-06-03

**Authors:** Clara Sgorbati, Eliana Lo Presti, Greta Bergamaschi, Monica Sani, Alessandro Volonterio

**Affiliations:** † Department of Chemistry, Materials, and Chemical Engineering “G. Natta”, Politecnico di Milano, Via Mancinelli 7, 20131 Milan, Italy; ‡ Consiglio Nazionale delle Ricerche, Istituto di Scienze e Tecnologie Chimiche “G. Natta” (SCITEC), Via Mario Bianco 9, 20131 Milan, Italy

## Abstract

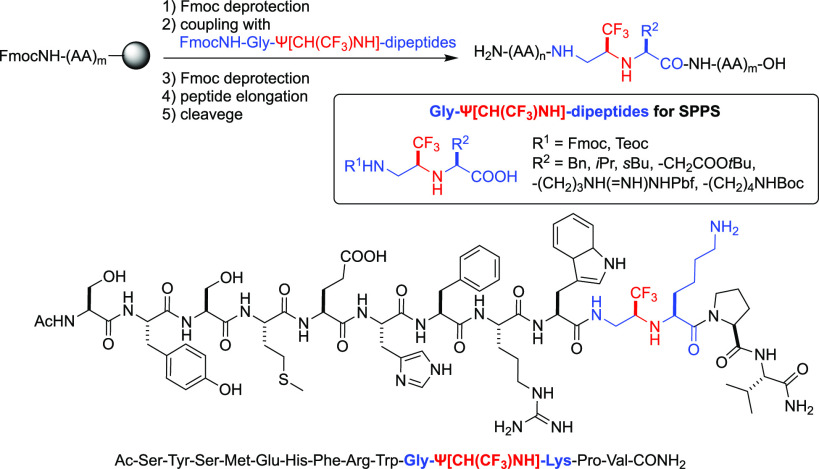

The solid-phase synthesis of Gly-Ψ[CH(CF_3_)NH]-peptides is presented. In order to achieve this goal, the synthesis of Gly-Ψ[CH(CF_3_)NH]-dipeptides having the C-terminus unprotected, the N-terminus protected as Fmoc- or Teoc-, and possibly side chain functionalities protected with acid-labile protecting groups has been developed. A selected small library of six peptidomimetics, encompassing analogues of biological relevant peptides, have been obtained in high purity.

The use of peptides as drugs, although rather desired due to their synthetic accessibility and selectivity being natural ligands for receptors and enzymes, is hampered mainly by their low metabolic stability as well as poor bioavailability.^1^ Among others, one efficient strategy in order to increase the stability of peptides to amide bond hydrolysis by proteases is the backbone modification.^2^ Accordingly, many peptide bond surrogates have been developed over the years, as well as synthetic strategies to site specifically incorporate them into a growing peptide.^3^ For drug discovery purposes and structure–activity studies, particularly important are those strategies that can be applied to solid-phase peptide synthesis (SPPS).^4^

In previous works, we introduced the stereogenic trifluoroethylamino function −CH(CF_3_)NH– as a hydrolytically stable peptide bond surrogate in which the carbonyl bond was replaced by the isopolar CH–CF_3_ bond (Figure 1).^5^ Besides the hydrolytic stability, the main characteristics featured by this unit that make it very promising for the replacement of the amide bond are (1) planarity and low basicity of the amino group;^5a^ (2) isopolarity of the CH–CF_3_ bond with the carbonyl bond;^5a^ (3) the good hydrogen bond donor ability of the NH moiety;^5c^ (4) the possibility to induce conformational orientation similar to the biologically active ones due to the bulkiness and spatial arrangement of the CF_3_ group;^5a^ and (5) the possibility to study the properties of the peptide by ^19^F NMR in solution and solid state^6^ (Figure 1). Indeed, this surrogate has been exploited at Merck-Frosst in Canada for the synthesis of Odanacatib, an inhibitor of Cathepsin K that reached phase III clinical trials for the treatment of postmenopausal osteoporosis.^7^

**Figure 1:**
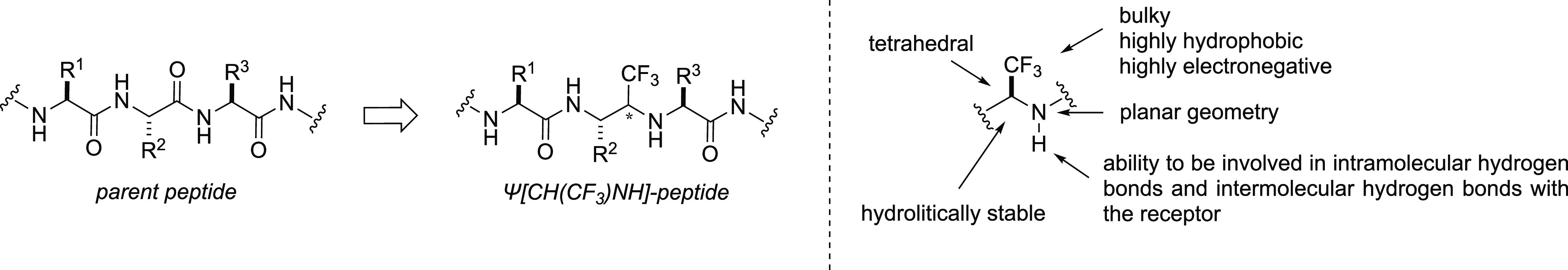
Ψ[CH(CF_3_)NH]-peptides and principal features of the trifluoroethylamine function.

To date, we have reported the solution phase synthesis of Gly-Ψ[NHCH(CF_3_)] partially modified retropeptides (PMR)^5a^ and Gly-Ψ[CH(CF_3_)NH]-peptides^5b,5c^ through stereoselective Michael addition of α-aminoesters to CF_3_-containing Michael acceptors. We have also developed the SPPS of Ψ[NHCH(CF_3_)] PMR peptides for which the synthetic pathway contains steps easily amenable to the solid phase.^8^ To address the impact of the trifluoroethylamino replacement for the native peptide bond on the structural properties, as well as on the activity of larger peptides, we describe herein the SPPS of this class of peptidomimetics.

Because the key steps for the synthesis of Gly-Ψ[CH(CF_3_)NH]-peptides are the Michael addition to *trans*-3,3,3-trifluoro-1-nitropropene **2**,^5b^ followed by reduction of the nitro group in heterogeneous conditions (not amenable to solid phase), we thought to develop the synthesis of suitable N^α^-protected dipeptides **4** as building blocks to be incorporated in the SPPS of larger Gly-Ψ[CH(CF_3_)NH]-peptides (Table 1). The right choice of the N-terminus and C-terminus protecting groups of the dipeptides is fundamental. On one hand, the N^α^-protecting group must be amenable to SPPS (we choose Fmoc and Teoc groups); on the other hand, the ester functionality must be orthogonal to the selected N^α^-protecting group and to any possible side chain protecting groups.

**Table 1:**
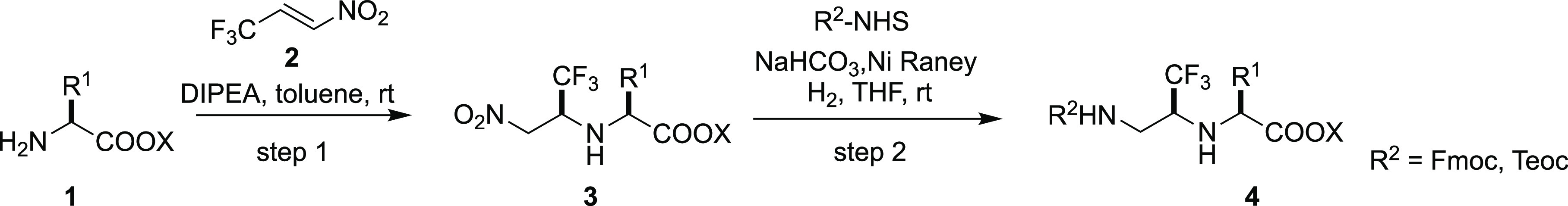

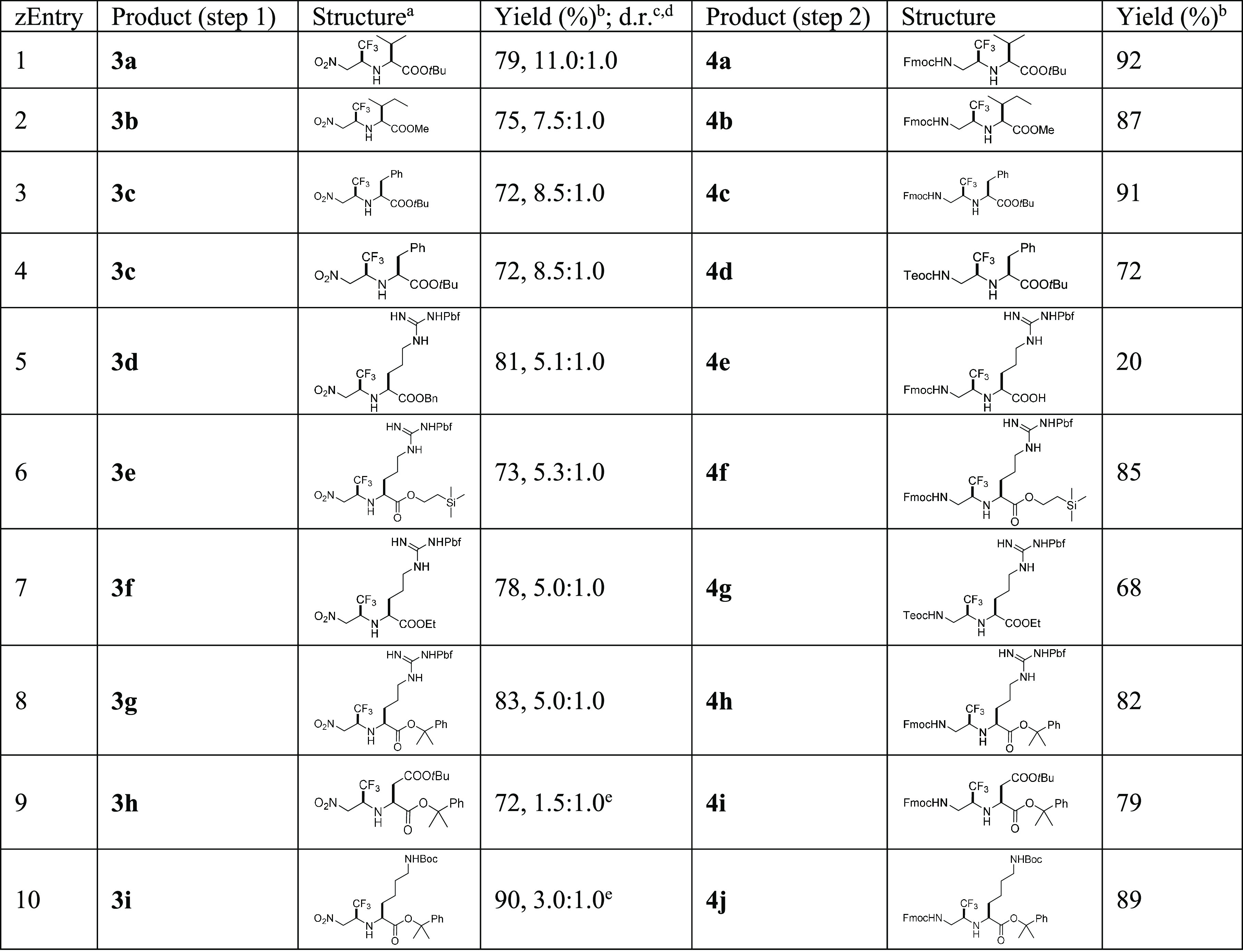
Solution-Phase Synthesis of Gly-Ψ[CH(CF_3_)NH]-Dipeptide Building Blocks

a Major diastereoisomer.

b Isolated yields.

c Diastereoisomeric ratio.

d Determined by integrating the ^19^F NMR signals.

e Step 1 performed in DCM.

Consequently, α-aminoesters **1** were reacted with trifluoromethyl-nitroalkene **2** in toluene in the presence of a catalytic amount of DIPEA, producing intermediate **3** as a mixture of diastereoisomers which in most cases are easily separated by flash chromatography (step 1, Table 1).^5b,9^ In step 2, the nitro group was hydrogenated in the presence of Ni-Raney and Fmoc-NHS (entries 1–3, 5, 6, 8–10, Table 1) or Teoc-NHS (entries 4, 7, Table 1), providing N-Fmoc and N-Teoc dipeptides **4** in good yields and without evidence of epimerization. A range of α-aminoesters **1** with different side chains and diverse ester groups were used. The following step was the selective hydrolysis of the ester function considering that the N-Fmoc protecting group is stable to acids, while the N-Teoc protecting group is stable also in base conditions. When the side chain of Gly-dipeptide **4** does not contain functional groups (entries 1–4, Table 1), the selective acidic hydrolysis of N-Fmoc/*tert*-butyl esters and the acid/basic hydrolysis of N-Teoc/alkyl esters can be selectively obtained. Accordingly, by treatment of dipeptides **4a**,**c** with a 30% solution of TFA/DCM overnight at rt, followed by coupling with H-Phe-OMe and H-Ile-OMe, respectively, promoted by HBTU, Gly-Ψ[CH(CF_3_)NH]-tripeptides **5a**,**b** were obtained in good overall yields (Scheme 1a,b).

**Scheme 1:**
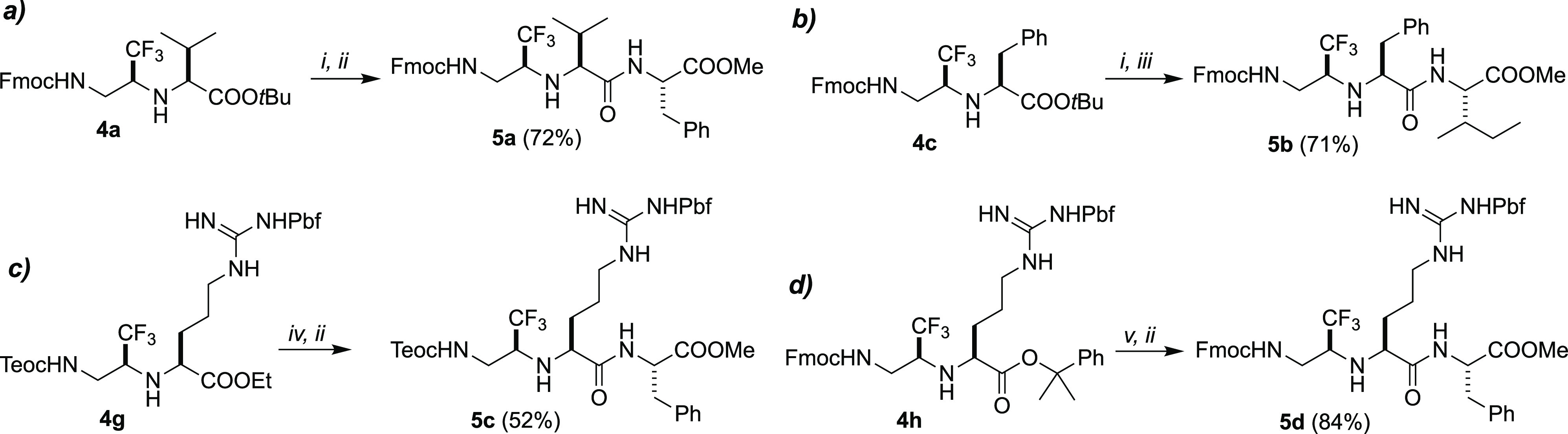
Selective Ester Deprotection and Solution Phase Coupling (*i*) 30% TFA, DCM, 12 h, rt; (*ii*) H-Phe-OMe, HBTU, DIPEA, DMF; (*iii*) H-Ile-OMe, HBTU, DIPEA, DMF; (*iv*) 1 M NaOH, THF, 24 h, rt; (*v*) 2% TFA, DCM, 1 h, rt.

However, when the side chain of Gly-dipeptide **4** contains functional groups bearing permanent acid labile protecting groups, like Pbf in Arg, Boc in Lys, and *tert*-Bu ester in Asp (entries 5–10, Table 1), the choice of alternative, suitable ester protecting groups is mandatory. For this reason, we prepared the starting intermediate **3d**–**g** from N^ω^-Pbf-Arg protected as benzyl, (trimethylsilyl)ethyl, ethyl, and 2-phenyl-isopropyl (cumyl)^4b^ esters, respectively (entries 5–8, Table 1). Treatment of O_2_N-Gly-Ψ[CH(CF_3_)NH]-N^ω^-Pbf-Arg-OBn **3e** with Ni-Raney in the presence of Fmoc-NHS resulted in the simultaneous reduction/Fmoc protection of the nitro group together with hydrogenolysis of the benzyl ester, leading to the formation of desired final dipeptide **4e** but in very low yield (entry 5, Table 1). All the attempts in order to selectively hydrolyze the (trimethylsilyl)ethyl ester in dipeptide **4f** with different fluoride containing reagents and reaction conditions failed, resulting in the cleavage also of the Fmoc protecting group (data not shown). Finally, both the basic hydrolysis of N-Teoc dipeptide ethyl ester **4g** and the hydrolysis of the cumyl ester **4h** in mild acid condition and short time (2% TFA, 1 h) were successful in the selective ester hydrolysis maintaining both the N^α^ and N^ω^ protecting groups. Indeed, Gly-Ψ[CH(CF_3_)NH]-tripeptides **5c**,**d** were obtained after selective ester hydrolysis of compound **4g**,**h**, respectively, followed by coupling with H-Phe-OMe, without clear evidence of N^α^/N^ω^ deprotection (Scheme 1c,d).

Once the deprotection/coupling protocols in solution phase were optimized, we turned our attention to the SPPS of the Gly-Ψ[CH(CF_3_)NH]-peptides. Even if both the procedures presented are suitable, we decided to use the Fmoc/O-cumyl (or O-tBu) strategy instead of Teoc/O-alkyl mainly for two re-sons: (1) although cumyl esters are not commercially available, in general, the synthesis of FmocNH-Gly-Ψ[CH(CF_3_)NH]-AA-OH displays higher overall yields, and (2) solid-phase Fmoc-deprotection can be easily monitored by UV.

We started with the synthesis of four tetrapeptides **6**–**9** incorporating FmocNH-Gly-Ψ[CH(CF_3_)NH]-AA-OH dipeptide building blocks coming from ester cleavage of **4c**,**i**,**j**,**h**, respectively, using 2-chlorotrityl chloride resin (entries 1–4, Table 2). These first attempts were made manually in a single vial using a DIC/Oxyma pure protocol for couplings, piperidine/DMF for Fmoc removal, and TFA/TIS/water/thioanisole mixture for resin cleavage. After HPLC purification, the peptides were obtained in high purity, acceptable yields, and without clear evidence of epimerization. Encouraged by these results, we transferred the protocol in a Biotage Alstra microwave automated synthesizer for the synthesis of longer and biologically relevant mimetics, in which the original peptide bond involving Gly amino acid is substituted with our fluorinated surrogate. In particular, we prepared analogues of opioid-binding Leu-enkephalin **10**, which was previously synthesized by multi-step synthesis in solution,^10^ and of the 12-mer peptide hormone α-melanotropin **11**. We were delighted to obtain the final peptides in good purity and yield also in these cases, demonstrating the suitability of SPPS for the preparation of Gly-Ψ[CH(CF_3_)NH]-peptides.

**Table 2:**
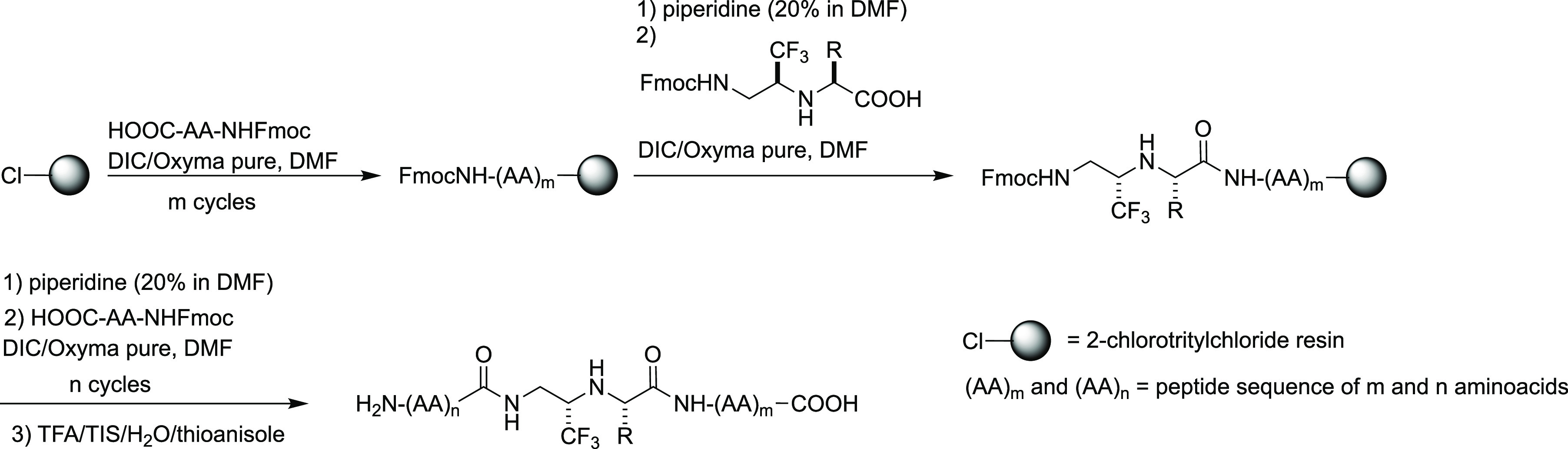
Solid-Phase Synthesis of Gly-Ψ[CH(CF_3_)NH]-Peptides

entry	FmocNH-Gly-Ψ[CH(CF_3_)NH]-dipeptide	peptide sequence	purity (%)^e^	mass, mg (yield, %)	calculated mass	observed mass
1	**4c**^a^	H_2_N-Tyr-GlyΨ[CH(CF_3_)NH]-Phe-Ala-OH, **6**^c^	96	9.4 (34)	510.51	511.2 [M + H]^+^
2	**4i**^b^	H_2_N-Tyr-GlyΨ[CH(CF_3_)NH]-Asp-Ala-OH, **7**^c^	97	10.3 (38)	478.17	479.2 [M + H]^+^
3	**4j**^b^	H_2_N-Tyr-GlyΨ[CH(CF_3_)NH]-Lys-Ala-OH, **8**^c^	95	8.4 (32)	491.51	492.2 [M + H]^+^
4	**4h**^b^	H_2_N-Tyr-GlyΨ[CH(CF_3_)NH]-Arg-Ala-OH, **9**^c^	96	20.3 (45)	519.53	520.3 [M + H]^+^
5	**4c**^a^	H_2_N-Tyr-Gly-GlyΨ[CH(CF_3_)NH]-Phe-Leu-OH, **10**^d^	96	15.3 (28)	609.65	610.4 [M + H]^+^
6	**4j**^b^	Ac-Ser-Tyr-Ser-Met-Glu-His-Phe-Arg-Trp-GlyΨ[CH(CF_3_)NH]-Lys-Pro-Val-CONH_2_, **11**^d^	95^f^	19.9 (25)	1719.82	859.9 [M + H]^2+^

a Treated with 30% TFA in DCM to form the free carboxylic acid from the precursor *tert-*Bu ester.

b Treated with 2% TFA in DCM to form the free carboxylic acid from the precursor cumyl ester.

c Synthesis performed manually in a single vial.

d Synthesis performed in an automated synthesizer.

e Determined by reverse phase HPLC.

f Improved to >98% after a second purification.

In conclusion, we have provided a general, highly efficient protocol for the SPPS of peptides containing the trifluoroethylamine unit as surrogate of a peptide bond involving glycine amino acid. All peptides, encompassing analogues of biologically relevant peptides, were obtained in acceptable yields and high purity. Since the key step for the synthesis of Gly-Ψ[CH(CF_3_)NH]-peptides envisages the reduction of a nitro group in which conditions are not compatible with solid-phase synthesis, we first developed a strategy for the preparation of dipeptides having the N^α^/side chain functional groups orthogonally protected and the free carboxylic acid at the C-terminus. The efficiency of the protocol will spur the application of the trifluoroethylamino peptide bond replacement in the combinatorial synthesis, physicochemical and biological properties studies, and high throughput screening of Gly-Ψ[CH(CF_3_)NH]-peptides.

## Experimental Procedures

### Materials

Commercially available reagent-grade solvents were employed without purification. CTC resin and N^α^-Fmoc-l-amino acids used during chain assembly were purchased from Iris Biotech GmbH (Marktredwitz, Germany). Ethyl cyanoglyoxylate-2-oxime (Oxyma) was purchased from Novabiochem (Darmstadt, Germany); *N*,*N*′-dimethylformamide (DMF) and trifluoroacetic acid (TFA) were from Carlo Erba (Rodano, Italy). *N*,*N*′-Diisopropylcarbodiimide (DIC), dichloromethane (DCM), and all other organic reagents and solvents, unless stated otherwise, were purchased in high purity from Sigma-Aldrich (Steinheim, Germany). All solvents for solid-phase peptide synthesis (SPPS) were used without further purification. HPLC grade acetonitrile (ACN) and ultrapure 18.2 Ω water (Millipore Milli-Q) were used for the preparation of all buffers for liquid chromatography. The chromatographic columns were from Phenomenex (Torrance CA, USA). All amino acids are of l-configuration unless otherwise stated. TLC were run on silica gel 60 F254 Merck. Visualization of the developed chromatogram was achieved with UV light and ceric ammonium molybdate (CAM) or ninhydrin stains. Flash chromatography (FC) was performed with silica gel 60 (60–200 μm, Merck). ^1^H, ^13^C, and ^19^F NMR spectra were run at 400 or 500 MHz. Chemical shifts are expressed in ppm (δ), using tetramethylsilane (TMS) as internal standard for ^1^H and ^13^C nuclei (δH and δC = 0.00), while C_6_F_6_ was used as external standard (δF −162.90) for ^19^F. ESI mass spectra were performed by a Bruker Esquire 3000+ instrument equipped with an MS detector composed by an ESI ionization source and a Single Quadrupole mass selective detector or by an Agilent Technologies 1200 Series HPLC system equipped with a DAD and a 6120 MS detector composed by an ESI ionization source and a Single Quadrupole mass selective detector. Optical rotations were measured on a Propol Digital Polarimeter with a sodium lamp.

Synthesis and characterization of compounds **2** and **3a**–**d** were reported in ref (5b). The synthesis of amino acid cumyl esters is described in ref (4b).

### Solution Phase Synthesis: General Procedures

#### Synthesis of Nitro-Michael Adducts **3**. Typical Procedure

To a stirred solution of **2** (0.76 mmol, 107 mg) and H-Ile-OMe hydrochloride (0.51 mmol, 93 mg) in toluene (7 mL) at rt was added DIPEA (0.56 mmol, 73 μL). After half an hour at rt, the solvent was removed in vacuo, and the crude was dissolved in EtOAc and washed once with 1 N HCl. The organic layer was dried over anhydrous Na_2_SO_4_. The solvent was removed in vacuo, and the crude was purified by FC (hexane/diisopropyl ether 9:1), affording 106 mg (75%) of the two pure diastereoisomers **(*****S*****)-3b** (*R*_*f*_ = 0.31, hexane/*iso-*Pr_2_O 7:3) and **(*****R*****)-3b** (*R*_*f*_ = 0.41, hexane/*iso-*Pr_2_O 7:3), in a 7.5:1 ratio as a colorless amorphous solids.

##### 2-(Trimethylsilyl)ethyl N^ω^-((2,2,4,6,7-Pentamethyl-2,3-Dihydrobenzofuran-5-yl)sulfonyl)-N^2^-((*S*)-1,1,1-trifluoro-3-nitropropan-2-yl)-l-argininate **3e**

Yellowish amorphous solid (253 mg, 73%). *R*_*f*_ 0.41 (60:40 AcOEt:hexane); [α]_D_^20^ +3.2° (*c* = 0.96, CHCl_3_); ^1^H NMR (500 MHz, CDCl_3_) δ 6.23–6.20 (br m, 2H), 6.06 (br s, 1H), 4.60 (dd, *J* = 13.5 and 4.5 Hz, 1H), 4.54 (dd, *J* = 13.5 and 7.4 Hz, 1H), 4.22–4.15 (m, 2H), 3.93 (br s, 1H), 3.41 (br s, 1H), 3.20–3.14 (br m, 2H), 2.96 (s, 2H), 2.56 (s, 3H), 2.49 (s, 3H), 2.11 (br s, 1H), 2.10 (s, 3H), 1.73–1.55 (m, 4H), 1.49 (s, 6H), 1.00 (t, *J* = 9.0 Hz, 2H), 0.05 (s, 9H); ^13^C{^1^H} NMR (125 MHz, CDCl_3_) δ 174.4, 159.1, 156.6, 138.6, 133.0, 132.5, 125.0, 124.9 (q, *J* = 281.2 Hz), 117.8, 86.7, 74.4, 64.2, 60.9, 57.8 (q, *J* = 30.0 Hz), 43.5, 41.1, 30.9, 28.8, 25.8, 19.4, 18.1, 17.7, 12.7, 1.24; ^19^F NMR (470 MHz, CDCl_3_): δ −76.1 (d, *J* = 7.4 Hz); MS (ESI) *m*/*z* 668.5 [M + H]^+^, 690.5 [M + Na]^+^; Anal. Calcd for C_27_H_44_F_3_N_5_O_7_SSi: C 48.56, H 6.64, N 10.49; found: C 48.54, H 6.63, N 10.50.

##### Ethyl N^ω^-((2,2,4,6,7-Pentamethyl-2,3-dihydrobenzofuran-5-yl)sulfonyl)-N^2^-((*S*)-1,1,1-trifluoro-3-nitropropan-2-yl)-l-argininate **3f**

Yellowish amorphous solid (232 mg, 78%). *R*_*f*_ 0.55 (80:20 AcOEt:hexane); [α]_D_^20^ −11.2° (*c* = 1.00, CHCl_3_); ^1^H NMR (400 MHz, CDCl_3_) δ 6.22–6.01 (br m, 3H), 4.60 (dd, *J* = 11.6 and 4.4 Hz, 1H), 4.53 (dd, *J* = 11.6 and 7.6 Hz, 1H), 4.14–4.09 (m, 2H), 3–94–3.91(br s, 1H), 3.44–3.42 (br s, 1H), 3.19–3.14 (m, 2H), 2.95 (s, 2H), 2.54 (s, 3H), 2.48 (s, 3H), 2.09 (s, 3H), 2.08 (br s, 1H), 1.73–1.70 (m, 4H), 1.46 (s, 6H), 1.22 (t, *J* = 7.2 Hz, 3H); ^13^C{^1^H} NMR (101 MHz, CDCl_3_) δ 174.0, 158.8, 156.4, 138.2, 132.9, 132.2, 124.7, 124.6 (q, *J* = 284.8 Hz), 117.5, 86.4, 74.1, 61.4, 60.4, 57.5 (q, *J* = 30.3 Hz), 43.2, 40.7, 30.8, 28.5, 25.5, 21.0, 17.8, 14.1, 12.3; ^19^F NMR (376 MHz, CDCl_3_): δ = −75.1 (d, *J* = 7.1 Hz). MS (ESI) *m*/*z* 596.4 [M + H]^+^, 618.3 [M + Na]^+^; Anal. Calcd for C_24_H_36_F_3_N_5_O_7_S: C 48.40, H 6.09, N 11.76; found: C 48.41, H 6.11, N 11.74.

##### 2-Phenylpropan-2-yl N^ω^-((2,2,4,6,7-Pentamethyl-2,3-dihydrobenzofuran-5-yl)sulfonyl)-N^2^-((*S*)-1,1,1-trifluoro-3-nitropropan-2-yl)-l-argininate **3g**

White amorphous solid (352 mg, 83%). *R*_*f*_ 0.50 (60:40 AcOEt:hexane); [α]_D_^20^ +5.5° (*c* = 1.00, CHCl_3_); ^1^H NMR (400 MHz, CDCl_3_) δ 7.25–7–19 (m, 5H), 5.93–5.88 (br m, 3H), 4.44 (dd, *J* = 13.6 and 4.8 Hz, 1H), 4.37 (dd, *J* = 13.6 and 7.2 Hz, 1H), 3.78 (br q, *J* = 6.0 Hz, 1H), 3.34 (br s, 1H), 3.11–3.07 (br m, 2H), 2.87 (s, 2H), 2.49 (s, 3H), 2.42 (s, 3H), 2.01 (s, 3H), 1.99 (br s, 1H), 1.70 (s, 3H), 1.69 (s, 3H), 1.54–1.45 (m, 4H), 1.37 (s, 6H); ^13^C{^1^H} NMR (101 MHz, CDCl_3_) δ 173.0, 158.8, 156.2, 144.9, 138.3, 132.8, 132.3, 128.4, 127.3, 124.7, 124.6 (q, *J* = 283.8 Hz), 124.3, 117.6, 86.5, 83.4, 73.9, 61.0, 57.6 (q, *J* = 30.3 Hz), 43.2, 40.8, 30.7, 28.6, 28.3, 28.1, 25.5, 19.2, 17.8, 12.4; ^19^F NMR (376 MHz, CDCl_3_): δ −75.0 (d, *J* = 7.1 Hz); MS (ESI) *m*/*z* 686.3 [M + H]^+^, 708.3 [M + Na]^+^, 724.3 [M + K]^+^; Anal. Calcd for C_31_H_42_F_3_N_5_O_7_S: C 54.30, H 6.17, N 10.21; found: C 54.31, H 6.15, N 10.21.

##### 4-(*tert*-Butyl) 1-(2-Phenylpropan-2-yl) ((*S*)-1,1,1-Trifluoro-3-nitropropan-2-yl)-l-aspartate **3h**

Mixture of diastereoisomers: yellowish gum (265 mg, 72%). *R*_*f*_ 0.60 (70:30 AcOEt:hexane); ^1^H NMR (400 MHz, CDCl_3_) δ (major diast.) 7.36–7.28 (m, 5H), 4.64 (dd, *J* = 13.6 and 4.8 Hz, 1H), 4.58 (dd, *J* = 13.6 and 4.0 Hz, 1H), 4.09–4.03 (m, 1H), 3.81 (dd, *J* = 8.0 and 4.4 Hz, 1H), 2.77 (dd, *J* = 16.0 and 4.0 Hz, 1H), 2.61 (dd, *J* = 16.4 and 4.0 Hz, 1H), 1.81 (s, 6H), 1.48 (s, 9H); ^1^H NMR (400 MHz, CDCl_3_) δ (minor diast.) 7.36–7.28 (m, 5H), 4.59 (dd, *J* = 13.2 and 6.0 Hz, 1H), 4.58 (dd, *J* = 13.2 and 9.2 Hz, 1H), 4.21–4.16 (m, 1H), 3.86 (dd, *J* = 8.0 and 4.0 Hz, 1H), 2.70 (dd, *J* = 16.4 and 4.0 Hz, 1H), 2.50 (dd, *J* = 16.4 and 8.0 Hz, 1H), 1.80 (s, 6H), 1.49 (s, 9H); ^13^C{^1^H} NMR (101 MHz, CDCl_3_) δ (major diast.) 171.7, 169.8, 144.9, 128.4, 127.3, 124.5 (q, *J* = 183.8 Hz),124.2 83.7, 81.7, 74.3, 58.0 (q, *J* = 30.3 Hz), 57.9, 40.0, 28.4, 28.1, 28.0; ^13^C{^1^H} NMR (101 MHz, CDCl_3_) δ (minor diast.) 171.0, 169.8, 144.8, 128.3, 127.3, 124.8 (q, *J* = 185.8 Hz), 124.2, 83.6, 81.6, 74.4, 57.9 (q, *J* = 29.3 Hz), 56.7, 40.2, 28.3, 28.1, 28.0; ^19^F NMR (376 MHz, CDCl_3_) δ (major diast.) −75.3 (d, *J* = 7.5 Hz); ^19^F NMR (376 MHz, CDCl_3_) δ (minor diast.) −74.3 (d, *J* = 3.7 Hz); MS (ESI) *m*/*z* 471.0.3 [M + Na]^+^, 487.1 [M + K]^+^; Anal. Calcd for C_20_H_27_F_3_N_2_O_6_: C 53.57, H 6.07, N 6.25; found: C 53.58, H 6.07, N 6.27.

##### 2-Phenylpropan-2-yl N^6^-(*tert*-Butoxycarbonyl)-N^2^-((*S*)-1,1,1-trifluoro-3-nitropropan-2-yl)-l-lysinate **3i**

Mixture of diastereoisomers: yellowish gum (312 mg, 90%). *R*_*f*_ 0.48 (70:30 AcOEt:hexane); ^1^H NMR (400 MHz, CDCl_3_) δ (major diast.) 7.27–7.16 (m, 5H), 4.50 (br s, 1H), 4.48 (dd, *J* = 14.0 and 5.2 Hz, 1H), 4.38 (dd, *J* = 13.6 and 5.2 Hz, 1H), 3.82–3.75 (m, 1H), 3.34 (dd, *J* = 8.0 and 5.2 Hz, 1H), 3.01 (br s, 2H), 1.96 (br s, 1H), 1.71 (s, 6H), 1.42–1.19 (m, 6H), 1.36 (s, 9H); ^1^H NMR (400 MHz, CDCl_3_) δ (minor diast.) 7.27–7.16 (m, 5H), 4.50 (br s, 1H), 4.49–4.42 (m, 1H), 4.26 (dd, *J* = 12.8 and 10.0 Hz, 1H), 3.92–3.85 (m, 1H), 3.27 (dd, *J* = 7.6 and 4.8 Hz, 1H), 3.01 (br s, 2H), 1.96 (br s, 1H), 1.70 (s, 6H), 1.42–1.19 (m, 6H), 1.36 (s, 9H); ^13^C{^1^H} NMR (101 MHz, CDCl_3_) δ (major diast.) 173.2, 156.1, 145.0, 128.4, 127.3, 124.6 (q, *J* = 283.8 Hz), 124.3, 83.2, 74.2, 61.2, 57.9 (q, *J* = 30.3 Hz), 40.3, 33.4, 29.7, 28.4, 28.1; ^13^C{^1^H} NMR (101 MHz, CDCl_3_) δ (minor diast.) 172.4, 156.1, 145.0, 128.4, 127.3, 124.2, 121.8 (q, *J* = 282.8 Hz), 83.1, 74.6, 60.4, 58.4 (q, *J* = 29.3 Hz), 40.3, 33.8, 29.7, 28.4, 27.9; ^19^F NMR (376 MHz, CDCl_3_) δ (major diast.) −75.3 (d, *J* = 7.5 Hz); ^19^F NMR (376 MHz, CDCl_3_) δ (minor diast.) −74.0 (d, *J* = 7.5 Hz); MS (ESI) *m*/*z* 528.2 [M + Na]^+^, 544.2 [M + K]^+^; Anal. Calcd for C_23_H_34_F_3_N_3_O_6_: C 54.65, H 6.78, N 8.31; found: C 54.66, H 6.80, N 8.29.

#### Synthesis of Fmoc- and Teoc-NH Dipeptides **4**. Typical Procedure

To a solution of **3a** (0.64 mmol, 200 mg) in THF (6.4 mL) were added solids Fmoc-NHS (0.83 mmol, 280 mg) and NaHCO_3_ (1.34 mmol, 113 mg) at rt. Ni-Raney (1 mL/mmol, slurry in H_2_O) was added, and the mixture was stirred under a hydrogen atmosphere overnight. The mixture was filtered on a Celite pad and eluted with AcOEt. The solution was washed with brine twice, dried over anhydrous Na_2_SO_4_, filtered, and concentrated under reduced pressure. The crude product was purified by FC (hexane/AcOEt 80:20), affording 298 mg (92%) of compound **4a** as a white amorphous solid.

##### *tert*-Butyl ((*S*)-3-((((9*H*-Fluoren-9-yl)methoxy)carbonyl)amino)-1,1,1-trifluoropropan-2-yl)-l-valinate **4a**

*R*_*f*_ 0.50 (30:70 AcOEt:hexane); [α]_D_^20^ +12.3° (*c* = 0.90, CHCl_3_); ^1^H NMR (400 MHz, CDCl_3_) δ 7.68 (d, *J* = 7.6 Hz, 2H), 7.57 (d, *J* = 7.2 Hz, 2H), 7.31 (t, *J* = 7.6 Hz, 2H), 7.22 (t, *J* = 7.6 Hz, 2H), 5.99 (br s, 1H), 4.29 (quintet, *J* = 7.2 Hz, 1H), 4.16 (t, *J* = 7.2 Hz, 1H), 3.55–3.48 (m, 1H), 3.26–3.24 (m, 1H), 3.13 (d, *J* = 4.0 Hz, 1H), 3.02–2.98 (m, 1H), 1.98–1.94 (m, 1H), 1.64 (br s, 1H), 1.41 (s, 9H), 0.93 (d, *J* = 6.8 Hz, 3H), 0.82 (d, *J* = 6.8 Hz, 3H); ^13^C{^1^H} NMR (101 MHz, CDCl_3_) δ 175.0, 156.7, 144.0, 141.3, 127.7, 127.0, 125.3, 119.9, 82.0, 67.1, 66.5, 59.1 (q, *J* = 27.3 Hz), 47.2, 40.2, 31.7, 28.1, 19.3, 17.3, the CF_3_ signal was obscured due to its low intensity; ^19^F NMR (376 MHz, CDCl_3_): δ −75.5 (d, *J* = 7.5 Hz); MS (ESI) *m*/*z* 507.3 [M + H]^+^, 529.3 [M + Na]^+^, 545.3 [M + K]^+^; Anal. Calcd for C_27_H_33_F_3_N_2_O_4_: C 64.02, H 6.57, N 5.53; found: C 64.01, H 6.59, N 5.53.

##### Methyl ((*S*)-3-((((9*H*-Fluoren-9-yl)methoxy)carbonyl)amino)-1,1,1-trifluoropropan-2-yl)-l-isoleucinate **4b**

Yellowish amorphous solid (105 mg, 87%). *R*_*f*_ 0.20 (20:80 AcOEt:hexane); [α]_D_^20^ +11.8° (*c* = 1.00, CHCl_3_); ^1^H NMR (400 MHz, CD_3_OD) δ 7.72 (d, *J* = 7.6 Hz, 2H), 7.62 (dd, *J* = 7.2 and 2.8 Hz, 2H), 7.32 (t, *J* = 7.6 Hz, 2H), 7.25 (t, *J* = 7.6 Hz, 2H), 4.35–4.32 (m, 2H), 4.18 (t, *J* = 6.8 Hz, 1H), 3.54 (s, 3H), 3.35 (dd, *J* = 14.0 and 3.6 Hz, 1H), 3.32–3.18 (m, 2H), 3.12–3.09 (m, 1H), 1.62–1.60 (m, 1H), 1.53–1.50 (m, 1H), 1.19–1.16 (m, 1H), 0.88–0.81 (m, 6H); ^13^C{^1^H} NMR (101 MHz, CD_3_OD) δ 175.2, 158.0, 144.0, 143.9, 141.2, 127.4, 126.7, 124.9, 119.5, 78.0, 66.7, 65.7, 59.1 (q, *J* = 27.3 Hz), 50.8, 39.1, 38.3, 24.8, 14.7, 10.3, the CF_3_ signal was obscured due to its low intensity; ^19^F NMR (376 MHz, CD_3_OD): δ −76.8 (d, *J* = 7.5 Hz); MS (ESI) *m*/*z* 501.2 [M + Na]^+^, 517.2 [M + K]^+^; Anal. Calcd for C_25_H_29_F_3_N_2_O_4_: C 62.75, H 6.11, N 5.85; found: C 62.77, H 6.10, N 5.86.

##### *tert*-Butyl ((*S*)-3-((((9*H*-Fluoren-9-yl)methoxy)carbonyl)amino)-1,1,1-trifluoropropan-2-yl)-l-phenylalaninate **4c**

White amorphous solid (98 mg, 91%). *R*_*f*_ 0.62 (60:40 *iso-*Pr_2_O:hexane); [α]_D_^20^ +7.8° (*c* = 1.00, CHCl_3_); ^1^H NMR (400 MHz, CDCl_3_) δ 7.68 (d, *J* = 7.6 Hz, 2H), 7.55 (d, *J* = 6.8 Hz, 2H), 7.32 (t, *J* = 7.6 Hz, 2H), 7.24–7.12 (m, 7H), 5.68 (br s, 1H), 4.28 (d, *J* = 7.2 Hz, 2H), 4.13 (t, *J* = 7.2 Hz, 1H), 3.60 (t, *J* = 6.0 Hz, 1H), 3.50–3.46 (m, 1H), 3.05–3.02 (m, 2H), 2.93 (dd, *J* = 13.6 and 5.6 Hz, 1H), 2.81 (dd, *J* = 13.6 and 7.6 Hz, 1H), 1.66 (br s, 1H), 1.35 (s, 9H); ^13^C{^1^H} NMR (101 MHz, CDCl_3_) δ 174.9, 156.6, 144.0, 141.3, 136.7, 129.3, 128.6, 127.0, 126.9, 125.3, 119.9, 82.4, 82.2, 67.0, 61.3, 58.5 (q, *J* = 28.3 Hz), 47.3, 39.9, 28.0, the CF_3_ signal was obscured due to its low intensity; ^19^F NMR (376 MHz, CDCl_3_): δ −75.2 (d, *J* = 7.1 Hz); MS (ESI) *m*/*z* 577.4 [M + Na]^+^, 593.4 [M + K]^+^; Anal. Calcd for C_31_H_33_F_3_N_2_O_4_: C 67.14, H 6.00, N 5.05; found: C 67.15, H 5.99, N 5.05.

##### *tert*-Butyl ((*S*)-1,1,1-Trifluoro-3-(((2-(trimethylsilyl)ethoxy)carbonyl)amino)propan-2-yl)-l-phenylalaninate **4d**

Yellowish gum (102 mg, 72%). *R*_*f*_ 0.75 (30:70 AcOEt:hexane); [α]_D_^20^ −1.8° (*c* = 1.00, CHCl_3_); ^1^H NMR (400 MHz, CDCl_3_) δ 7.26–7.14 (m, 5H), 5.35 (br s, 1H), 4.11 (t, *J* = 8.4 Hz, 2H), 3.61 (t, *J* = 6.8 Hz, 1H), 3.51–3.48 (m, 1H), 3.10–3.03 (m, 2H), 2.93 (dd, *J* = 13.6 and 6.4 Hz, 1H), 2.84 (dd, *J* = 7.2 Hz, 1H), 1.70 (br s, 1H), 1.37 (s, 9H), 0.94 (t, *J* = 8.4 Hz, 2H), 0.00 (s, 9H); ^13^C{^1^H} NMR (101 MHz, CDCl_3_) δ, 175.2, 158.4, 138.2, 130.8, 129.9, 128.3, 127.1 (q, *J* = 284.8 Hz), 83.5, 64.7, 63.0, 59.9 (q, *J* = 27.3 Hz), 41.4, 29.4, 19.2, 0.0; ^19^F NMR (376 MHz, CDCl_3_): δ −75.1 (br s); MS (ESI) *m*/*z* 499.2 [M + Na]^+^, 515.2 [M + K]^+^; Anal. Calcd for C_22_H_35_F_3_N_2_O_4_Si: C 55.44, H 7.40, N 5.88; found: C 55.45, H 7.41, N 5.86.

##### N^2^-((*S*)-3-((((9*H*-Fluoren-9-yl)methoxy)carbonyl)amino)-1,1,1-trifluoropropan-2-yl)-N^ω^-((2,2,4,6,7-pentamethyl-2,3-dihydrobenzofuran-5-yl)sulfonyl)-l-arginine **4e**

Yellowish gum (26 mg, 20%). *R*_*f*_ 0.15 (80:20 AcOEt:hexane); [α]_D_^20^ −8.8° (*c* = 1.00, CH_3_OH); ^1^H NMR (400 MHz, CD_3_OD) δ 7.73 (d, *J* = 7.6 Hz, 2H), 7.63 (d, *J* = 7.6 Hz, 2H), 7.34 (t, *J* = 7.6 Hz, 2H), 7.27 (t, *J* = 7.6 Hz, 2H), 4.30 (d, *J* = 7.2 Hz, 2H), 4.19 (t, *J* = 7.2 Hz, 1H), 3.46–3.44 (m, 1H), 3.40–3.38 (m, 1H), 3.29–3.26 (m, 1H), 3.20–3.16 (m, 2H), 2.91 (s, 3H), 2.59 (s, 3H), 2.52 (s, 3H), 2.05 (s, 3H), 1.71–1.55 (m, 4H), 1.38 (s, 6H); ^13^C{^1^H} NMR (101 MHz, CD_3_OD) δ 174.9, 157.1, 156.3, 155.0, 142.4, 139.6, 136.6, 130.7, 125.9, 125.3, 123.4, 123.2, 118.0, 115.7, 84.8, 76.5, 65.4, 58.6, 57.0 (q, *J* = 26.3 Hz), 45.5, 41.0, 37.8, 28.8, 25.8, 16.8, 15.5, 9.7; ^19^F NMR (376 MHz, CD_3_OD): δ −76.2 (d, *J* = 7.1 Hz); MS (ESI) *m*/*z* 760.4 [M + H]^+^, 782.4 [M + Na]^+^, 798.4 [M + K]^+^; Anal. Calcd for C_37_H_44_F_3_N_5_O_7_S: C 58.49, H 5.84, N 9.22; found: C 58.50, H 5.83, N 9.20.

##### 2-(Trimethylsilyl)ethyl N^2^-((*S*)-3-((((9*H*-Fluoren-9-yl)methoxy)carbonyl)amino)-1,1,1-trifluoropropan-2-yl)-N^ω^-((2,2,4,6,7-pentamethyl-2,3-dihydrobenzofuran-5-yl)sulfonyl)-l-argininate **4f**

Yellowish amorphous solid (134 mg, 85%). *R*_*f*_ 0.36 (80:20 AcOEt:hexane); [α]_D_^20^ −15.2° (*c* = 1.00, CHCl_3_); ^1^H NMR (400 MHz, CD_3_OD) δ 7.84 (d, *J* = 7.6 Hz, 2H), 7.74 (d, *J* = 7.6 Hz, 2H), 7.43 (t, *J* = 7.6 Hz, 2H), 7.35 (t, *J* = 7.6 Hz, 2H), 4.40–4.37 (m, 2H), 4.32–4.29 (m, 1H), 4.21–4.18 (m, 2H), 3.52–3.48 (m, 1H), 3.41–3.39 (m, 1H), 3.32–3.29 (m, 2H), 3.23–3.19 (m, 2H), 3.01 (s, 2H), 2.62 (s, 3H), 2.56 (s, 3H), 2.11 (s, 3H), 1.72–1.68 (m, 2H), 1.63–1.58 (m, 2H), 1.48 (s, 6H), 1.00 (t, *J* = 8.8 Hz, 2H), 0.00 (s, 9H); ^13^C{^1^H} NMR (101 MHz, CD_3_OD) δ 174.9, 158.4, 157.9, 143.95, 143.91, 141.2, 138.0, 133.0, 132.1, 127.4, 126.7, 125.0, 124.6, 119.5, 117.0, 86.2, 78.0, 66.9, 63.0, 60.4, 58.5 (q, *J* = 26.3 Hz), 47.0, 42.6, 30.2, 27.3, 18.1, 17.0, 16.9, 11.1, −2.9, the CF_3_ signal was obscured due to its low intensity; ^19^F NMR (376 MHz, CD_3_OD): δ −76.7 (d, *J* = 7.1 Hz); MS (ESI) *m*/*z* 882.5 [M + Na]^+^; Anal. Calcd for C_42_H_56_F_3_N_5_O_7_SSi: C 58.65, H 6.56, N 8.14; found: C 58.64, H 6.56, N 8.15.

##### Ethyl N^ω^-((2,2,4,6,7-Pentamethyl-2,3-dihydrobenzofuran-5-yl)sulfonyl)-N^2^-((*S*)-1,1,1-trifluoro-3-(((2-(trimethylsilyl)ethoxy)carbonyl)amino)propan-2-yl)-l-argininate **4g**

White amorphous solid (154 mg, 68%). *R*_*f*_ 0.50 (80:20 AcOEt:hexane); [α]_D_^20^ −4.6° (*c* = 1.00, CHCl_3_); ^1^H NMR (400 MHz, CDCl_3_) δ 6.24–6.21 (m, 3H), 5.60 (br s, 1H), 4.13–409 (m, 5H), 3.47–3.44 (m, 1H), 3.38–3.35 (m, 1H), 3.18–3.13 (m, 3H), 2.91 (s, 2H), 2.53 (s, 3H), 2.47 (s, 3H), 2.06 (s, 3H), 1.68–1.54 (m, 4H), 1.47 (s, 6H), 1.22 (t, *J* = 7.2 Hz, 3H), 0.95 (t, *J* = 6.4 Hz, 2H), 0.00 (s, 9H); ^13^C{^1^H} NMR (101 MHz, CDCl_3_) δ 176.5, 172.7, 160.4, 158.8, 157.7, 139.9, 133.8, 127.3 (q, *J* = 283.8 Hz), 126.2, 119.0, 87.9, 78.8, 64.9, 62.8, 61.9, 44.7, 42.3, 32.1, 30.1, 27.2, 22.5, 20.7, 19.3, 19.2, 15.7, 13.9, 0.00; ^19^F NMR (376 MHz, CDCl_3_): δ −75.2 (d, *J* = 7.2 Hz); MS (ESI) *m*/*z* 709.5 [M + H]^+^; Anal. Calcd for C_30_H_50_F_3_N_5_O_7_SSi: C 50.76, H 7.10, N 9.87; found: C 50.77, H 7.10, N 9.90.

##### 2-Phenylpropan-2-yl N^2^-((*S*)-3-((((9*H*-Fluoren-9-yl)methoxy)carbonyl)amino)-1,1,1-trifluoropropan-2-yl)-N^ω^-((2,2,4,6,7-pentamethyl-2,3-dihydrobenzofuran-5-yl)sulfonyl)-l-argininate **4h**

White amorphous solid (256 mg, 82%). *R*_*f*_ 0.60 (60:40 AcOEt:hexane); [α]_D_^20^ −12.2° (*c* = 1.00, CHCl_3_); ^1^H NMR (400 MHz, CD_3_OD) δ 7.81 (d, *J* = 7.2 MHz, 2H), 7.63 (d, *J* = 7.6 Hz, 1H), 7.60 (d, *J* = 7.6 Hz, 1H), 7.42–7.39 (m, 2H), 7.33–7.27 (m, 2H), 7.23–7.20 (m, 2H), 7.14 (t, *J* = 7.6 Hz, 2H), 7.10–7.06 (m, 1H), 4.27–4.18 (m, 2H), 4.10 (t, *J* = 6.8 Hz, 1H), 3.40–3.36 (m, 2H), 3.22–3.20 (m, 4H), 2.97 (s, 2H), 2.60 (s, 3H), 2.53 (s, 3H), 2.07 (s, 3H), 1.70 (s, 3H), 1.67 (s, 3H), 1.59–1.45 (m, 4H), 1.43 (s, 6H); ^13^C{^1^H} NMR (101 MHz, CDCl_3_) δ 173.1, 158.8, 156.2, 144.9, 138.3, 132.8, 132.3, 128.4, 127.3, 124.7, 124.6 (q, *J* = 283.8 Hz), 124.3, 86.5, 83.4, 74.0, 61.0, 60.4, 57.6 (q, *J* = 30.3 Hz), 43.2, 40.8, 30.7, 28.6, 28.3, 28.1, 25.5, 21.0, 19.2, 17.8, 14.2, 12.4; ^19^F NMR (376 MHz, CD_3_OD): δ −76.5 (d, *J* = 7.6 Hz); MS (ESI) *m*/*z* 878.5 [M + H]^+^, 900.5 [M + Na]^+^, 916.4 [M + K]^+^; Anal. Calcd for C_46_H_54_F_3_N_5_O_7_S: C 62.93, H 6.20, N 7.98; found: C 62.94, H 6.21, N 7.98.

##### 4-(*tert*-Butyl) 1-(2-Phenylpropan-2-yl) ((*S*)-3-((((9*H*-Fluoren-9-yl)methoxy)carbonyl)amino)-1,1,1-trifluoropropan-2-yl)-l-aspartate **4i**

Yellowish gum (121 mg, 79%). *R*_*f*_ 0.62 (30:70 AcOEt:hexane); [α]_D_^20^ +7.2° (*c* = 1.00, CHCl_3_); ^1^H NMR (400 MHz, CD_3_OD) δ 7.76 (d, *J* = 7.6 Hz, 2H), 7.57 (t, *J* = 7.6 Hz, 2H), 7.36–7.32 (m, 2H), 7.27–7.04 (m, 7H), 4.35–4.33 (m, 1H), 4.22–4.16 (m, 2H), 4–08–4.05 (m, 1H), 3.70 (t, *J* = 6.4 Hz, 1H), 3.38 (dd, *J* = 14.0 and 3.6 Hz, 1H), 3.18 (dd, *J* = 13.2 and 3.6 Hz, 1H), 2.65 (dd, *J* = 15.6 and 5.2 Hz, 1H), 2.51 (dd, *J* = 15.6 and 7.2 Hz, 1H), 1.66 (s, 3H), 1.65 (s, 3H), 1.40 (s, 9H); ^13^C{^1^H} NMR (101 MHz, CD_3_OD) δ 172.0, 170.2, 157.8, 145.2, 144.0, 141.2, 127.9, 127.8, 127.3, 126.7, 126.4 (q, *J* = 282.1 Hz), 125.0, 124.0, 119.4, 82.9, 81.0, 66.7, 58.0 (q, *J* = 30.2 Hz), 57.6, 39.3, 27.6, 27.4, 26.9, 16.9, 16.8; ^19^F NMR (376 MHz, CD_3_OD): δ −76.6 (d, *J* = 7.6 Hz); MS (ESI) *m*/*z* 663.4 [M + Na]^+^, 679.4 [M + K]^+^; Anal. Calcd for C_35_H_39_F_3_N_2_O_6_: C 65.61, H 6.14, N 4.37; found: C 65.60, H 6.15, N 4.39.

##### 2-Phenylpropan-2-yl N^2^-((*S*)-3-((((9*H*-Fluoren-9-yl)methoxy)carbonyl)amino)-1,1,1-trifluoropropan-2-yl)-N^6^-(*tert*-butoxycarbonyl)-l-lysinate **4j**

White amorphous solid (201 mg, 89%). *R*_*f*_ 0.44 (30:70 AcOEt:hexane); [α]_D_^20^ +13.4° (*c* = 1.00, CHCl_3_); ^1^H NMR (400 MHz, CD_3_OD) δ 7.81 (d, *J* = 7.6 Hz, 2H), 7.66 (d, *J* = 7.6 Hz, 2H), 7.41–7.38 (m, 4H), 7.34–7.30 (m, 4H), 7.24–7.20 (m, 1H), 4.40–4.36 (m, 2H), 4.23 (t, *J* = 6.8 Hz, 1H), 3.44–3.41 (m, 1H), 3.33–3.28 (m, 2H), 3.25–3.22 (m, 1H), 3.00–2.96 (m, 2H), 1.78 (s, 6H), 1.70–1.64 (m, 2H), 1.43–1.32 (m, 4H), 1.41 (s, 9H); ^13^C{^1^H} NMR (101 MHz, CDCl_3_) δ 174.4, 156.8, 156.0, 145.0, 144.0, 141.3, 128.3, 127.7, 127.3, 127.0, 126.1 (q, *J* = 283.8 Hz), 125.3, 124.3, 119.9, 83.2, 79.1, 77.3, 67.1, 60.8, 58.9 (q, *J* = 28.3 Hz), 47.2, 40.3, 40.0, 33.2, 29.8, 28.4, 22.8; ^19^F NMR (376 MHz, CD_3_OD): δ −75.4 (d, *J* = 7.6 Hz); MS (ESI) *m*/*z* 720.5 [M + Na]^+^; Anal. Calcd for C_38_H_46_F_3_N_3_O_6_: C 65.41, H 6.64, N 6.02; found: C 65.41, H 6.65, N 6.00.

### Selective Ester Deprotection and Synthesis of Tripeptides **5**

#### Typical Procedure for the *tert*-Butyl Ester Hydrolysis and Solution Phase Coupling

Dipeptide **4a** (0.20 mmol, 100 mg) was dissolved in a mixture of TFA/DCM (30% v/v, 2.0 mL). The solution was stirred at rt until complete consumption of the starting material (TLC monitoring). The solvents were evaporated and co-evaporated twice with cyclohexane. The crude was dissolved in DMF (2.0 mL) and HBTU (0.24 mmol, 91 mg), DIPEA (0.40 mmol, 125 μL) and H-Phe-OMe hydrochloride (0.24 mmol, 52 mg) were added. The solution was stirred at rt overnight. The reaction was diluted with HCl 1 N and extracted with AcOEt. The combined organic layers were washed twice with water, once with sat bicarbonate solution and twice with brine. The organic phase was dried over anhydrous Na_2_SO_4_, filtered, and concentrated under reduced pressure. The crude product was purified by FC (hexane/AcOEt 80:20), affording 88 mg (72%) of compound **5a** as a white amorphous solid.

##### Methyl ((*S*)-3-((((9*H*-Fluoren-9-yl)methoxy)carbonyl)amino)-1,1,1-trifluoropropan-2-yl)-l-valyl-l-phenylalaninate **5a**

*R*_*f*_ 0.50 (30:70 AcOEt:hexane); [α]_D_^20^ +26.5° (*c* = 1.00, CHCl_3_); ^1^H NMR (400 MHz, CD_3_OD) δ 7.74 (d, *J* = 7.6 Hz, 2H), 7.61 (t, *J* = 7.6 Hz, 2H), 7.36–7.34 (m, 2H), 7.29–7.26 (m, 2H), 7.07–7.04 (m, 5H), 4.67 (dd, *J* = 10.4 and 5.2 Hz, 1H), 4.33 (d, *J* = 6.8 Hz, 2H), 4.17 (t, *J* = 6.8 Hz, 1H), 3.62 (s, 3H), 3.16–3.09 (m, 2H), 3.02 (dd, *J* = 14.4 and 8.0 Hz, 1H), 2.89 (d, *J* = 6.8 Hz, 1H), 2.81 (dd, *J* = 13.6 and 10.4 Hz, 1H), 2.63–2.59 (m, 1H), 1.64 (octet, *J* = 6.8 Hz, 1H), 0.79 (d, *J* = 6.8 Hz, 3H), 0.78 (d, *J* = 6.8 Hz, 3H); ^13^C{^1^H} NMR (101 MHz, CDCl_3_) δ 173.4, 173.2, 157.2, 143.8, 141.4, 136.4, 129.1, 128.6, 127.8, 127.1, 127.0, 125.0, 120.1, 77.2, 67.2, 67.0, 58.2, 52.6, 47.2, 40.2, 37.5, 31.4, 19.1, 17.6, the CF_3_ and C-CF_3_ signals were obscured due to their low intensity; ^19^F NMR (376 MHz, CD_3_OD): δ −76.2 (d, *J* = 7.6 Hz); MS (ESI) *m*/*z* 612.4 [M + H]^+^, 634.4 [M + Na]^+^; Anal. Calcd for C_33_H_36_F_3_N_3_O_5_: C 64.80, H 5.93, N 6.87; found: C 64.78, H 5.94, N 6.87.

##### Methyl ((*S*)-3-((((9*H*-Fluoren-9-yl)methoxy)carbonyl)amino)-1,1,1-trifluoropropan-2-yl)-l-phenylalanyl-l-isoleucinate **5b**

Yellowish amorphous solid (156 mg, 71%). *R*_*f*_ 0.20 (20:80 AcOEt:hexane); [α]_D_^20^ +15.5° (*c* = 1.00, CHCl_3_); ^1^H NMR (400 MHz, CDCl_3_) δ 7.69 (d, *J* = 7.6 Hz, 2H), 7.56–7.50 (m, 2H), 7.33 (t, *J* = 7.6 Hz, 2H), 7.24–7.12 (m, 8H), 5.93 (br s, 1H), 4.59 (dd, *J* = 9.2 and 5.2 Hz, 1H), 4.40–4.37 (m, 1H), 4.21–4.18 (m, 1H), 4.12–4.08 (m, 1H), 3.75–3.71 (m, 1H), 3.67 (s, 3H), 3.53–3.51 (m, 1H), 3.30–3.27 (m, 1H), 3.14–3.10 (m, 2H), 2.73 (dd, *J* = 13.6 and 8.8 Hz, 1H), 1.86–1.82 (m, 1H), 1.75 (br s, 1H), 1.33–1.28 (m, 1H), 1.10–1.06 (m, 1H), 0.81–0.78 (m, 6H); ^13^C{^1^H} NMR (101 MHz, CDCl_3_) δ 173.8, 173.0, 157.1, 143.9, 143.7, 141.3, 136.1, 129.1, 128.8, 127.8, 127.2, 127.1, 126.0 (q, *J* = 284.1 Hz), 120.0, 67.1, 62.2, 56.2, 52.4, 47.2, 40.5, 39.7, 37.7, 25.1, 15.6, 11.5; ^19^F NMR (376 MHz, CDCl_3_): δ −73.7 (d, *J* = 7.6 Hz); MS (ESI) *m*/*z* 626.4 [M + H]^+^, 648.4 [M + Na]^+^, 664.4 [M + K]^+^; Anal. Calcd for C_33_H_36_F_3_N_3_O_5_: C 64.80, H 5.93, N 6.87; found: C 64.78, H 5.94, N 6.87.

#### Typical Procedure for the Methyl Ester Hydrolysis and Solution Phase Coupling

Dipeptide **4g** (0.20 mmol, 150 mg) was dissolved in THF (1 mL), and a 1 N NaOH aqueous solution (1 mL) was added at rt. The solution was stirred at rt until complete consumption of the starting material (TLC monitoring). The solution was diluted with 1 N aqueous HCl until acid pH and extracted with AcOEt. The combined organic layers were dried over anhydrous Na_2_SO_4_, filtered, and concentrated under reduced pressure. The crude was dissolved in DMF (2.0 mL) and HBTU (0.24 mmol, 91 mg), DIPEA (0.40 mmol, 125 μL) and H-Phe-OMe hydrochloride (0.24 mmol, 52 mg) were added. The solution was stirred at rt overnight. The reaction was diluted with HCl 1 N and extracted with AcOEt. The combined organic layers were washed twice with water, once with sat bicarbonate solution and twice with brine. The organic phase was dried over anhydrous Na_2_SO_4_, filtered, and concentrated under reduced pressure. The crude product was purified by FC (hexane/AcOEt 20:80), affording 91 mg (52%) of compound **5c** as a white amorphous solid.

##### Methyl N^ω^-((2,2,4,6,7-Pentamethyl-2,3-dihydrobenzofuran-5-yl)sulfonyl)-N^2^-((*S*)-1,1,1-trifluoro-3-(((2-(trimethylsilyl)ethoxy)carbonyl)amino)propan-2-yl)-l-arginyl-l-phenylalaninate **5c**

*R*_*f*_ 0.30 (80:20 AcOEt:hexane); [α]_D_^20^ −6.3° (*c* = 0.90, CHCl_3_); ^1^H NMR (400 MHz, CD_3_OD) δ 7.17–7.13 (m, 5H), 4.71 (dd, *J* = 10.0 and 5.2 Hz, 1H), 4.11 (t, *J* = 8.0 Hz, 2H), 3.66 (s, 3H), 3.27–3.23 (m, 2H), 3.19 (dd, *J* = 14.0 and 8.0 Hz, 1H), 3.07–3.00 (m, 3H), 2.94–2.92 (m, 2H), 2.71–2.68 (m, 1H), 2.53 (s, 3H), 2.46 (s, 3H), 2.03 (s, 3H), 1.39 (s, 9H), 0.96 (t, *J* = 8.0 Hz, 2H), 0.00 (s, 9H); ^13^C{^1^H} NMR (101 MHz, CD_3_OD) δ 172.0, 158.5, 156.7, 138.0, 137.0, 133.0, 132.1, 128.9, 128.8, 128.2, 128.1, 126.5, 124.6, 117.1, 86.2, 78.1, 62.9, 60.8, 57.9 (q, *J* = 26.3 Hz), 53.4, 51.5, 42.6, 36.8, 30.5, 27.3, 18.2, 17.3, 17.0, 11.1, −2.8; ^19^F NMR (376 MHz, CD_3_OD): δ −75.8 (d, *J* = 7.6 Hz); MS (ESI) *m*/*z* 865.6 [M + Na]^+^; Anal. Calcd for C_38_H_57_F_3_N_6_O_8_SSi: C 54.14, H 6.82, N 9.97; found: C 54.12, H 6.81, N 9.99.

#### Typical Procedure for the Cumyl Ester Hydrolysis and Solution Phase Coupling

Dipeptide **4h** (0.20 mmol, 175 mg) was dissolved in a mixture of TFA/DCM (2% v/v, 2.0 mL). The solution was stirred at rt for 1 h. The solvents were evaporated and co-evaporated twice with cyclohexane. The crude was dissolved in DMF (2.0 mL) and HBTU (0.24 mmol, 91 mg), DIPEA (0.40 mmol, 125 μL) and H-Phe-OMe hydrochloride (0.24 mmol, 52 mg) were added. The solution was stirred at rt overnight. The reaction was diluted with HCl 1 N and extracted with AcOEt. The combined organic layers were washed twice with water, once with sat bicarbonate solution and twice with brine. The organic phase was dried over anhydrous Na_2_SO_4_, filtered, and concentrated under reduced pressure. The crude product was purified by FC (hexane/AcOEt 30:70), affording 155 mg (84%) of compound **5d** as a white amorphous solid.

##### Methyl N^2^-((*S*)-3-((((9*H*-Fluoren-9-yl)methoxy)carbonyl)amino)-1,1,1-trifluoropropan-2-yl)-N^ω^-((2,2,4,6,7-pentamethyl-2,3-dihydrobenzofuran-5-yl)sulfonyl)-l-arginyl-l-phenylalaninate **5d**

*R*_*f*_ 0.38 (80:20 AcOEt:hexane); [α]_D_^20^ −13.2° (*c* = 1.00, CHCl_3_); ^1^H NMR (400 MHz, CDCl_3_) δ 7.88 (br d, *J* = 8.4 Hz, 1H), 7.79 (d, *J* = 7.6 Hz, 2H), 7.67–7.62 (m, 2H), 7.43 (t, *J* = 7.6 Hz, 2H), 7.35–7.29 (m, 2H), 7.15–7.11 (m, 5H), 6.32 (br s, 2H), 6.02 (br s, 1H), 5.51 (br s, 1H), 4.86–4.78 (m, 1H), 4.46–4.38 (m, 2H), 4.24–4.20 (m, 1H), 3.75 (s, 3H), 3.45 (br s, 1H), 3.26–3.15 (m, 4H), 2.98–2.92 (m, 4H), 2.64 (s, 3H), 2.56 (br s, 1H), 2.55 (s, 3H), 2.12 (s, 3H), 1.71–1.39 (m, 4H), 1.47 (s, 6H); ^13^C{^1^H} NMR (101 MHz, CDCl_3_) δ 172.6, 158.9, 157.1, 156.6, 144.0, 143.8, 141.4, 138.4, 132.8, 132.3, 129.4, 128.4, 127.8, 127.1, 126.8, 125.2, 125.0, 124.7, 120.0, 117.6, 86.4, 66.8, 60.6, 58.2 (q, *J* = 26.4 Hz), 53.4, 52.6, 47.2, 43.3, 39.7, 37.2, 28.6, 25.4, 19.3, 17.9, 12.5; ^19^F NMR (376 MHz, CDCl_3_): δ −75.2 (br s); MS (ESI) *m*/*z* 943,4 [M + Na]^+^; Anal. Calcd for C_47_H_55_F_3_N_6_O_8_S: C 61.29, H 6.02, N 9.12; found: C 61.30, H 6.00, N 9.10.

### Solid Phase Peptide Synthesis: General Procedures

#### Synthesis of Peptides **6**–**9**: General Procedures

##### CTC Resin Loading

CTC resin (1.6 mmol/g loading) was swollen in CH_2_C_l2_ for 30 min and then washed with DMF (3 × 3 mL). A solution of entering Fmoc-amino acid (0.5 equiv) and DIEA (2.5 equiv) in NMP (3 mL) was added, and the resin was shaken at rt for 2 h. The resin was washed with DMF (2 × 3 mL), and capping was performed by treatment with methanol/DIEA in DCM (1 × 30 min). The resin was then washed with DMF (2 × 3 mL), CH_2_C_l2_ (2 × 3 mL), and DMF (2 × 3 mL). The resin was subsequently submitted to manual peptide assembly (Fmoc-SPPS).

##### Loading Estimation of the First Amino Acid

The resin was treated with piperidine/DMF (1:5, v/v, 3 mL, 2 × 5 min) and then washed with DMF (5 × 3 mL). The combined deprotection and washings solution were made up to 25 mL with DMF. The solution was diluted 50-fold with DMF, and the UV absorbance of the piperidine-fulvene adduct was measured (λ = 301 nm, ε = 7800 M^–1^ cm^–1^) to estimate the amount of amino acid loaded onto the resin.

##### Peptide Assembly via Manually SPPS

Peptides were assembled manually in a 0.1 mmol scale. Activation of entering Fmoc-protected amino acids was performed using 0.5 M Oxyma in DMF/0.5 M DIC in DMF (1:1:1 molar ratio), with a 5 equiv excess over the initial resin loading. Coupling steps were performed for 45 min at room temperature. Fmoc-deprotection steps were performed by treatment with a 20% piperidine solution in DMF at room temperature (1 × 10 min). Following each coupling or deprotection step, peptidyl resin was washed with DMF (2 × 3.5 mL), DCM (1 × 3.5 mL), and DMF (2 × 3.5 mL). Upon complete chain assembly, resin was washed with DCM (5 × 3.5 mL) and gently dried under nitrogen flow. The cleavage step was performed as indicated below.

#### Synthesis of Peptides **10**–**11**

##### Resin Loading

Resin (0.5 mmol/g loading) was swollen in CH_2_Cl_2_ for 30 min and then washed with DMF (3 × 3 mL). A solution of entering Fmoc-amino acid, HBTU, and DIEA (1:1:2, 5 equiv over resin loading) in NMP (3 mL) was added, and the resin was shaken at rt for 4 h. The resin was washed with DMF (2 × 3 mL), and capping was performed by treatment with acetic anhydride/DIEA in DCM (1 × 30 min). The resin was then washed with DMF (2 × 3 mL), CH_2_Cl_2_ (2 × 3 mL), and DMF (2 × 3 mL). The resin was subsequently submitted to fully automated iterative peptide assembly (Fmoc-SPPS).

##### Loading Estimation of the First Amino Acid

The resin was treated with piperidine/DMF (1:5, v/v, 3 mL, 2 × 5 min) and then washed with DMF (5 × 3 mL). The combined deprotection and washings solution were made up to 25 mL with DMF. The solution was diluted 50-fold with DMF, and the UV absorbance of the piperidine-fulvene adduct was measured (λ = 301 nm, ε = 7800 M^–1^ cm^–1^) to estimate the amount of amino acid loaded onto the resin.

#### Peptide Assembly via Iterative Fully Automated Microwave Assisted SPPS

Peptides were assembled by stepwise microwave-assisted Fmoc-SPPS on a Biotage ALSTRA Initiator + peptide synthesizer, operating in a 0.1 mmol scale. Activation of entering Fmoc-protected amino acid (0.3 M solution in DMF) was performed using 0.5 M Oxyma in DMF/0.5 M DIC in DMF (1:1:1 molar ratio), with a 5 equiv excess over the initial resin loading. Coupling steps were performed for 30 min at 30 °C. Fmoc-deprotection steps were performed by treatment with a 20% piperidine solution in DMF at room temperature (1 × 10 min). Following each coupling or deprotection step, peptidyl-resin was washed with DMF (4 × 3.5 mL). Upon complete chain assembly, resin was washed with DCM (5 × 3.5 mL) and gently dried under a nitrogen flow. The cleavage step was performed as indicated below.

##### Cleavage from the Resin

Resin-bound peptide was treated with an ice-cold TFA, TIS, water, and thioanisole mixture (92.5:2.5:2.5:2.5 v/v/v/v, 4 mL). After gently shaking the resin for 3 h at room temperature, the resin was filtered and washed with neat TFA (2 × 4 mL). The combined cleavage solutions were worked-up as indicated below.

##### Workup and Purification

The cleavage mixture was concentrated under a nitrogen stream and then added dropwise to ice-cold diethyl ether (40 mL) to precipitate the crude peptide. The crude peptide was collected by centrifugation and washed with further cold diethyl ether to remove scavengers. Peptide was then dissolved in 0.1% TFA aqueous buffer (with addition of ACN to aid dissolution, if necessary). Residual diethyl ether was removed by a gentle nitrogen stream.

Peptides were purified on a reverse-phase preparative Shimadzu HPLC Pominence system equipped with an FRC-10A fraction collector and UV–vis detector (monitoring at 230 and 280 nm), using a Shimadzu C18, 10 um, 250 × 20 mm column. Gradients were run using a solvent system consisting of A: 97.5% H_2_O, 2.5% ACN, 0.7% TFA; eluent B: 30% H_2_O, 70% ACN, 0.7% TFA, and pure fractions were lyophilized on a Christ Alpha 2-4 LO plus freeze-dryer and analyzed by ESI-MS.

Pure peptides were analyzed on a Shimadzu Prominece reverse-phase HPLC (RP-HPLC) system equipped with Shimadzu LC-20AD pumps, and a Shimadzu SPD-M20A UV–vis detector using a Shimadzu C18, 5 μm, 150 × 4.6 mm column at a flow rate of 1 mL/min. RP-HPLC gradients were run using a solvent system consisting of solution A (97.5% H_2_O, 2.5% ACN, 0.7% TFA) and B (30% H_2_O, 70% ACN, 0.7% TFA). Analytical RP-HPLC data are reported as column retention time (tR) in minutes (min).

## Supporting Information Available

Copies of ^1^H, ^19^F, ^13^C NMR, and MS spectra for all new compounds, and analytical HPLC traces for peptides (PDF)
