# Supplementary material for: Solid-Phase Synthesis of Gly-Ψ[CH(CF3)NH]-Peptides
Source: J Org Chem. 2021 Jun 3;86(13):9225–32. doi: 10.1021/acs.joc.1c00853 (PMC8279481; doi:10.1021/acs.joc.1c00853)

SUPPORTING INFORMATION

Solid-Phase Synthesis of Gly-Ψ[CH(CF_3)NH]-Peptides.

Clara Sgorbati,[†] Eliana Lo Presti,[§] Greta Bergamaschi,[§] Monica Sani,[§] Alessandro Volonterio*,[†,§]

[†]Department of Chemistry, Materials, and Chemical Engineer "G. Natta", Politecnico di Milano, Via Mancinelli 7, 20131 Milan, Italy;

[§]Consiglio Nazionale delle Ricerche, Istituto di Scienze e Tecnologie Chimiche "G. Natta" (SCITEC), Via Mario Bianco 9, 20131 Milan, Italy

Corresponding author: alessandro.volonterio@polimi.it

Table of contents

Page S2-S47	Copies of ^{1}H, ^{13}C, ^{19}F NMR and MS spectra of compounds **3e-i**, **4a-j** and **5a-d**
Pages S48-S54	Copies of the HPLC chromatograms and MS spectra of peptides **6-11**

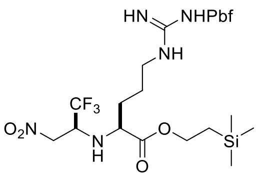

3e ^{1}H NMR (500 MHz, $CDCl_3$); ^{19}F NMR (470 MHz, $CDCl_3$)

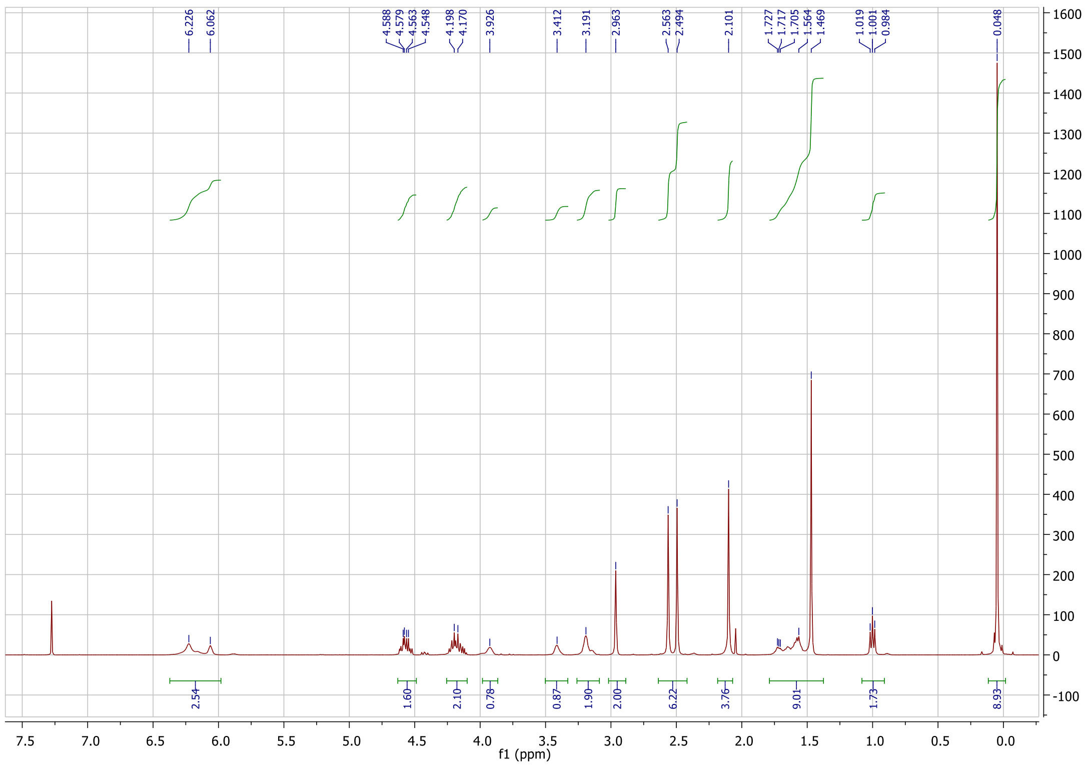

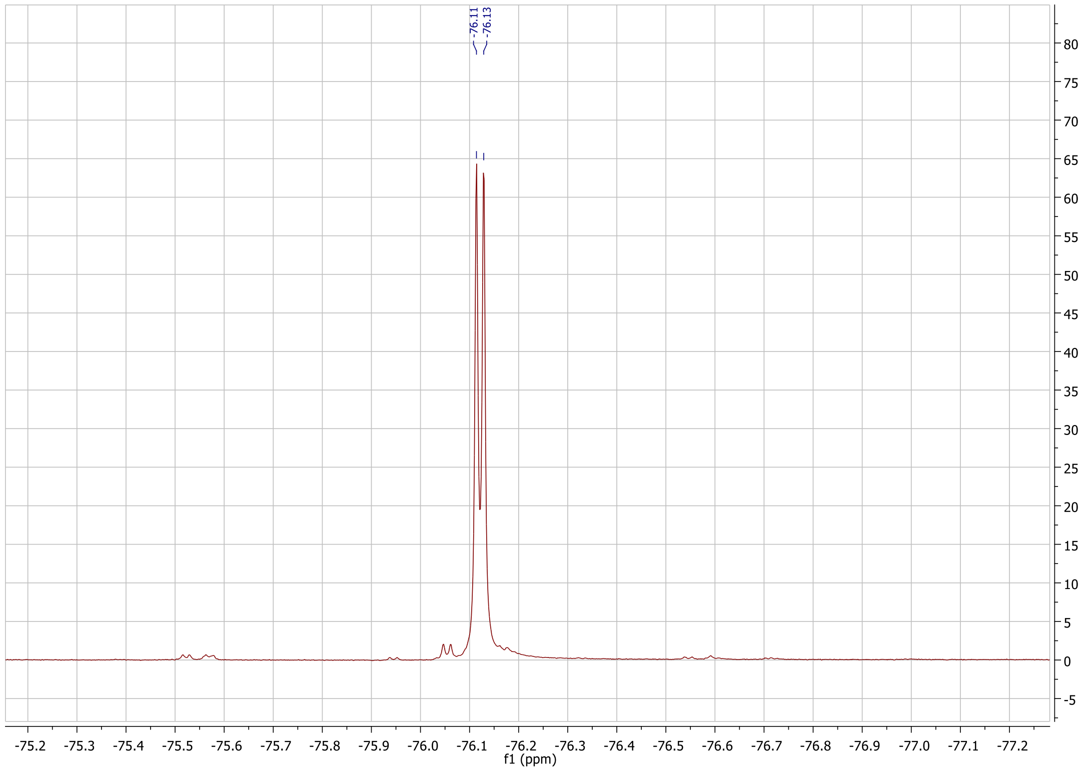

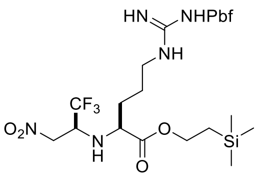

3e $^{13}C\{^{1}H\}$ NMR (125 MHz, $CDCl_3$)

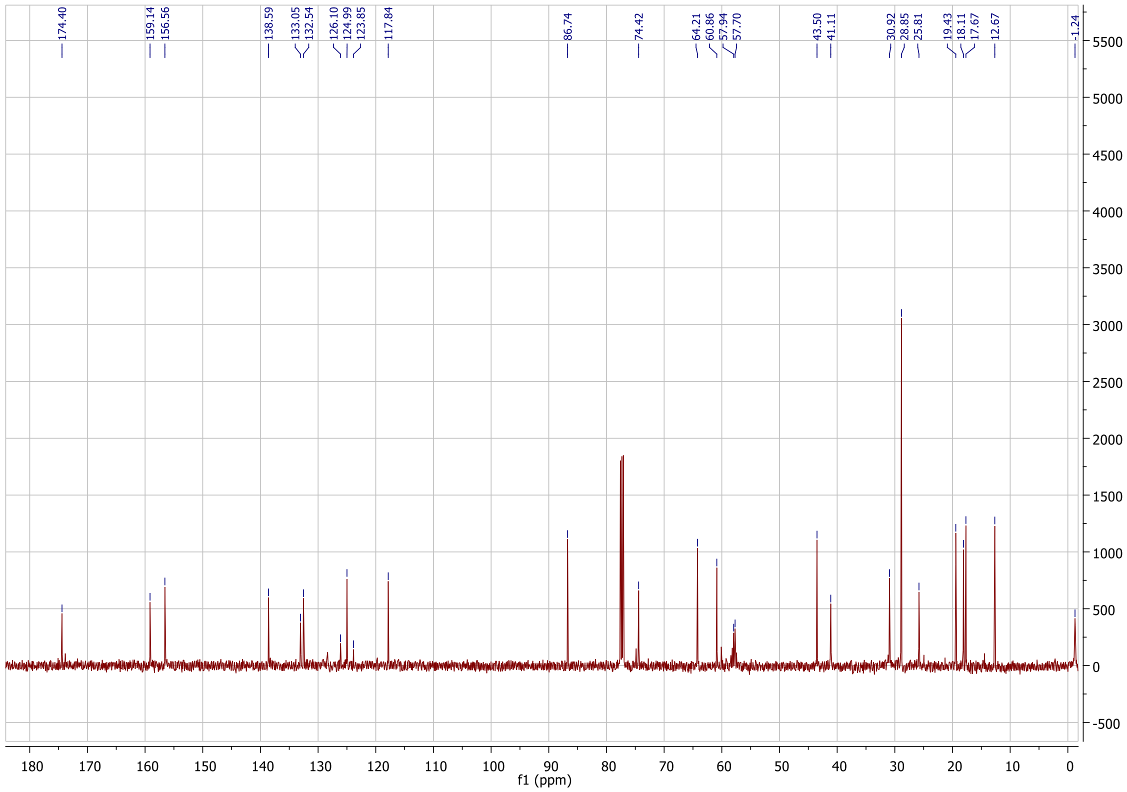

-L.G.S. - Laboratorio Grandi Strumenti - Display Report

Analysis Name	av cs03.d	Acquisition Date	03/12/19 15:43:44	Operator	Walter Panzeri
Sample Name		Method	Copy of _01tmix_posneg Im.MS	Instrument	esquire3000plus
Comment	1 mg/ml dil 1:100 MeOH Richiedente: Volonterio				

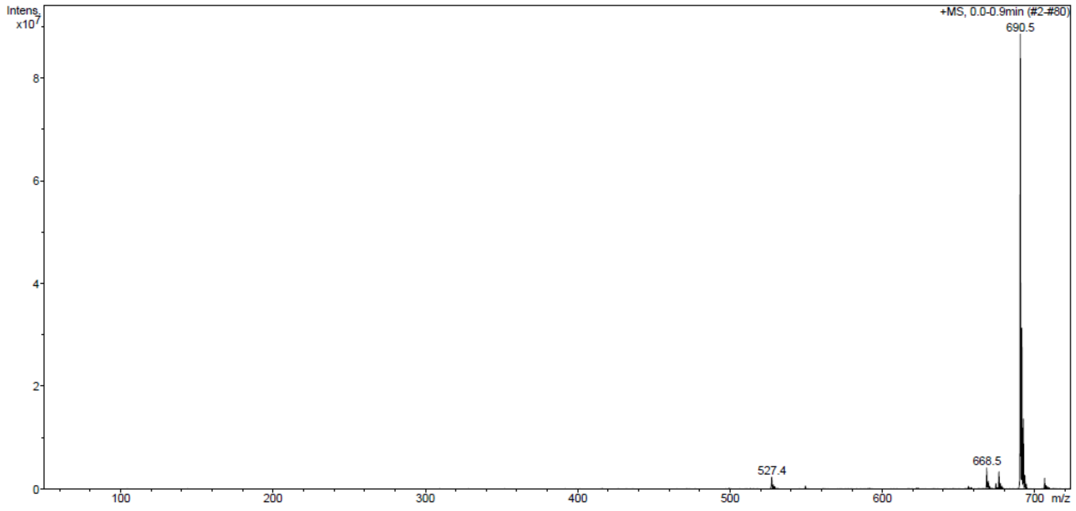

Bruker Daltonics DataAnalysis 3.1 printed: 03/12/19 15:45:00 Page 1 of 1

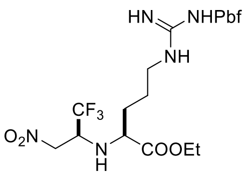

3f ^{1}H NMR (400 MHz, $CDCl_3$); ^{19}F NMR (376 MHz, $CDCl_3$)

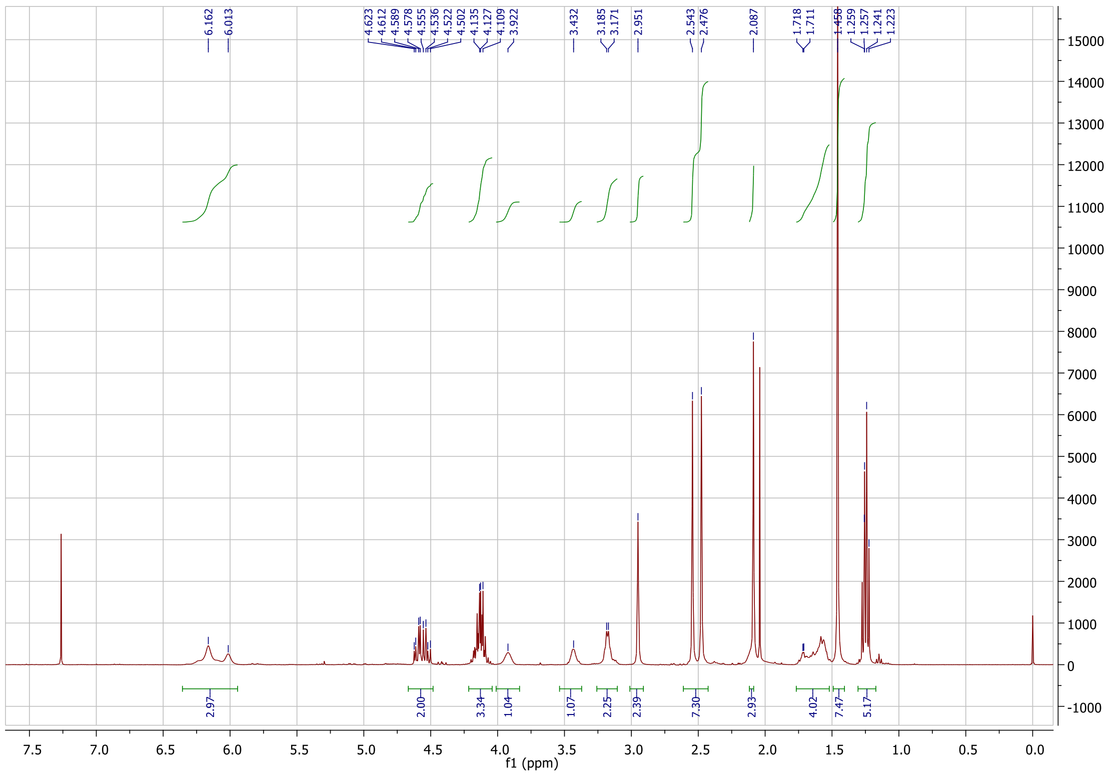

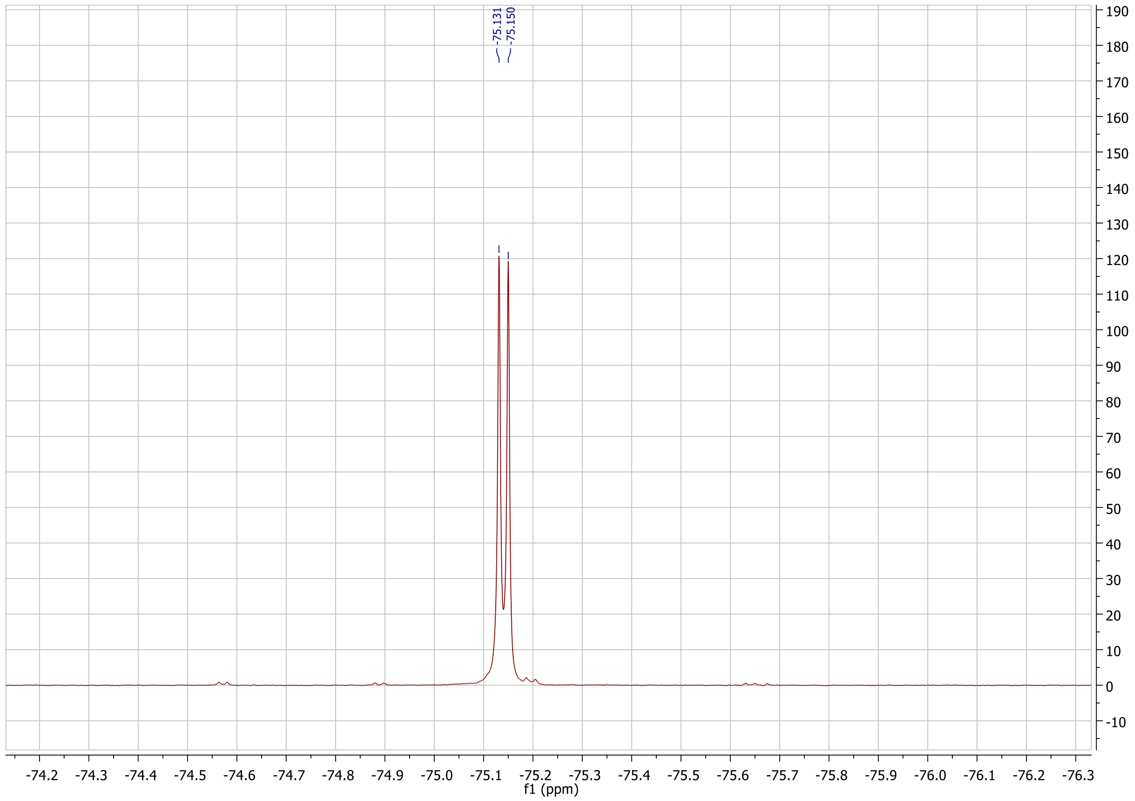

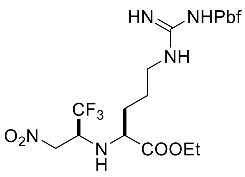

3f $^{13}C\{^{1}H\}$ NMR (101 MHz, $CDCl_3$)

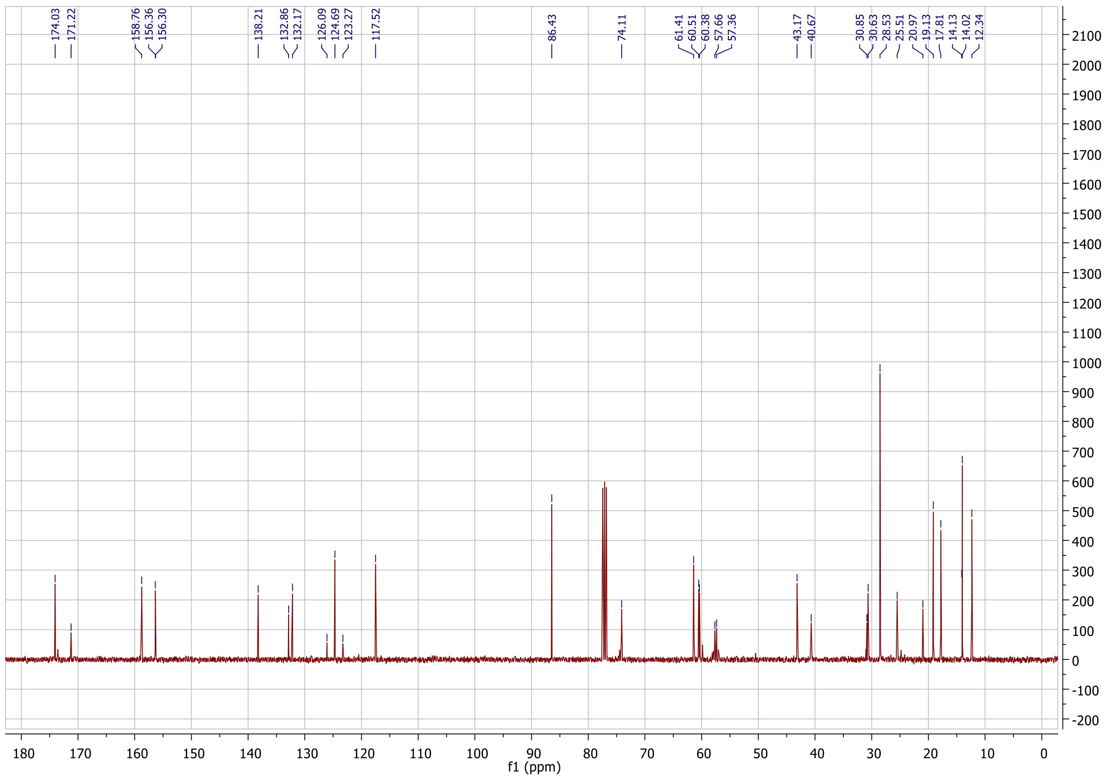

-L.G.S. - Laboratorio Grandi Strumenti - Display Report

Analysis Name	av cs23.d	Acquisition Date	11/20/19 12:06:35	Operator	Walter Panzeri
Sample Name		Method	Copy of _01tmix_posneg lm.MS	Instrument	esquire3000plus
Comment	1mg/ml dil 1:100 CH3CN Richiedente: Sgorbati				

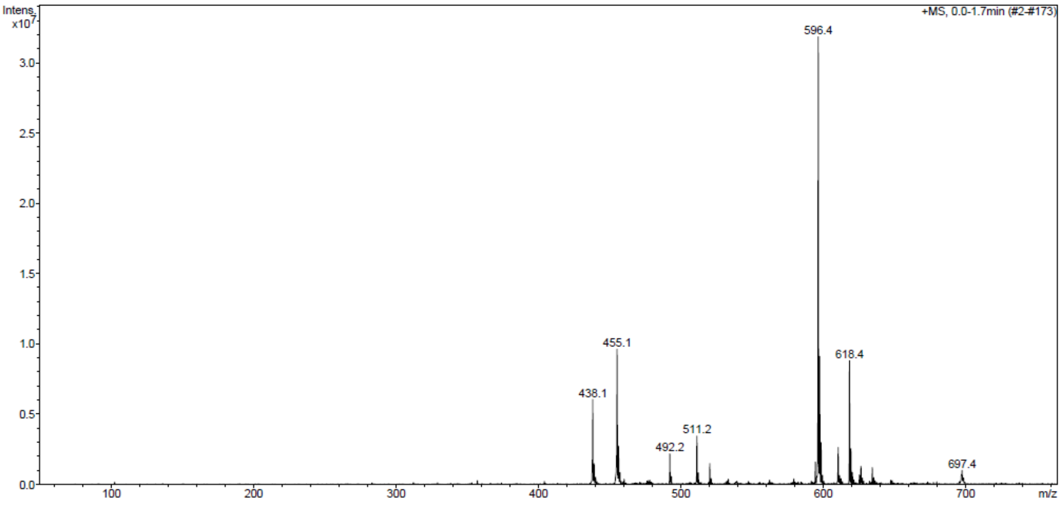

Bruker Daltonics DataAnalysis 3.1 printed: 11/20/19 12:08:41 Page 1 of 1

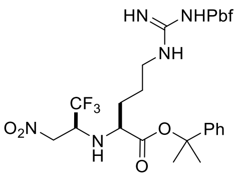

3g ^{1}H NMR (400 MHz, $CDCl_3$); ^{19}F NMR (376 MHz, $CDCl_3$)

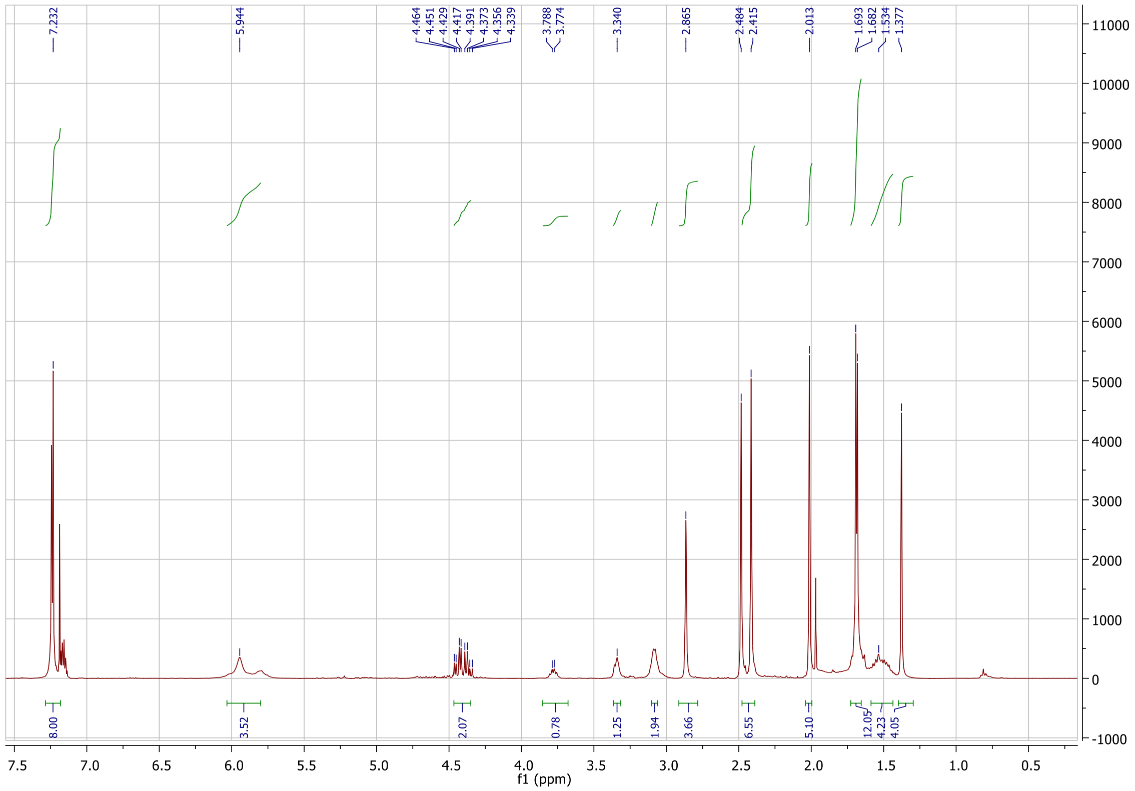

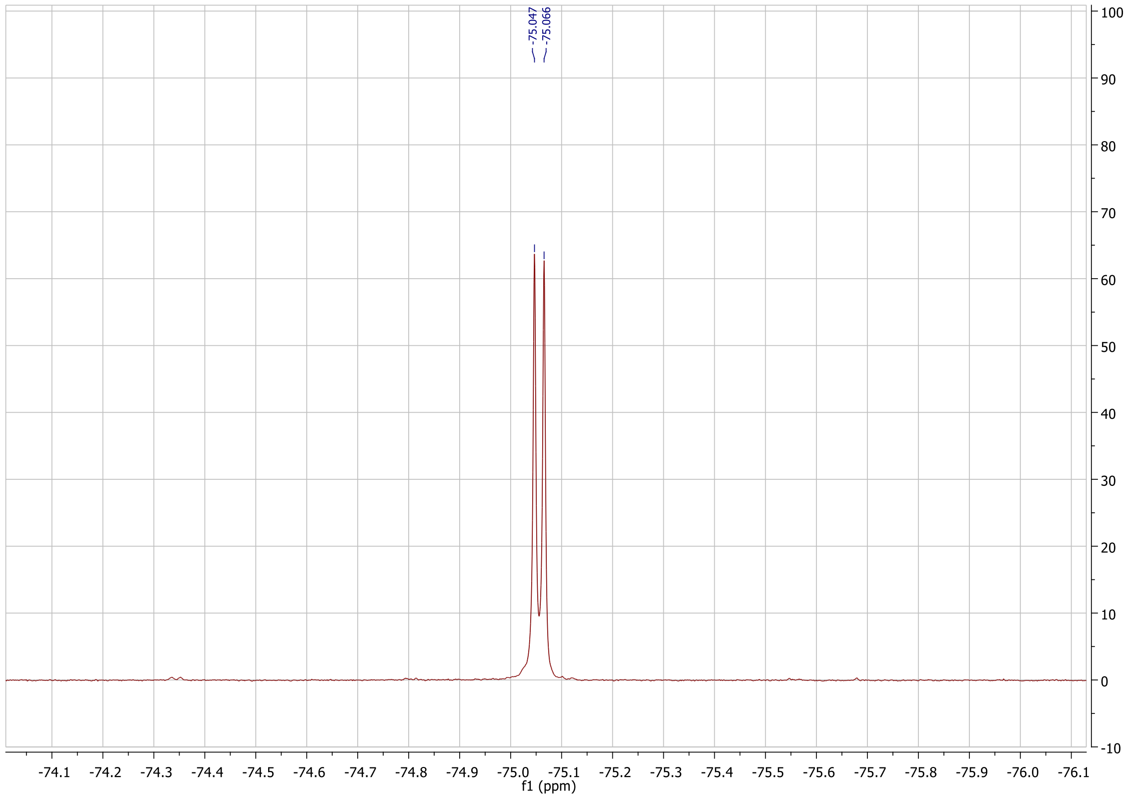

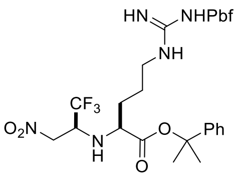

3g $^{13}C\{^1H\}$ NMR (101 MHz, $CDCl_3$)

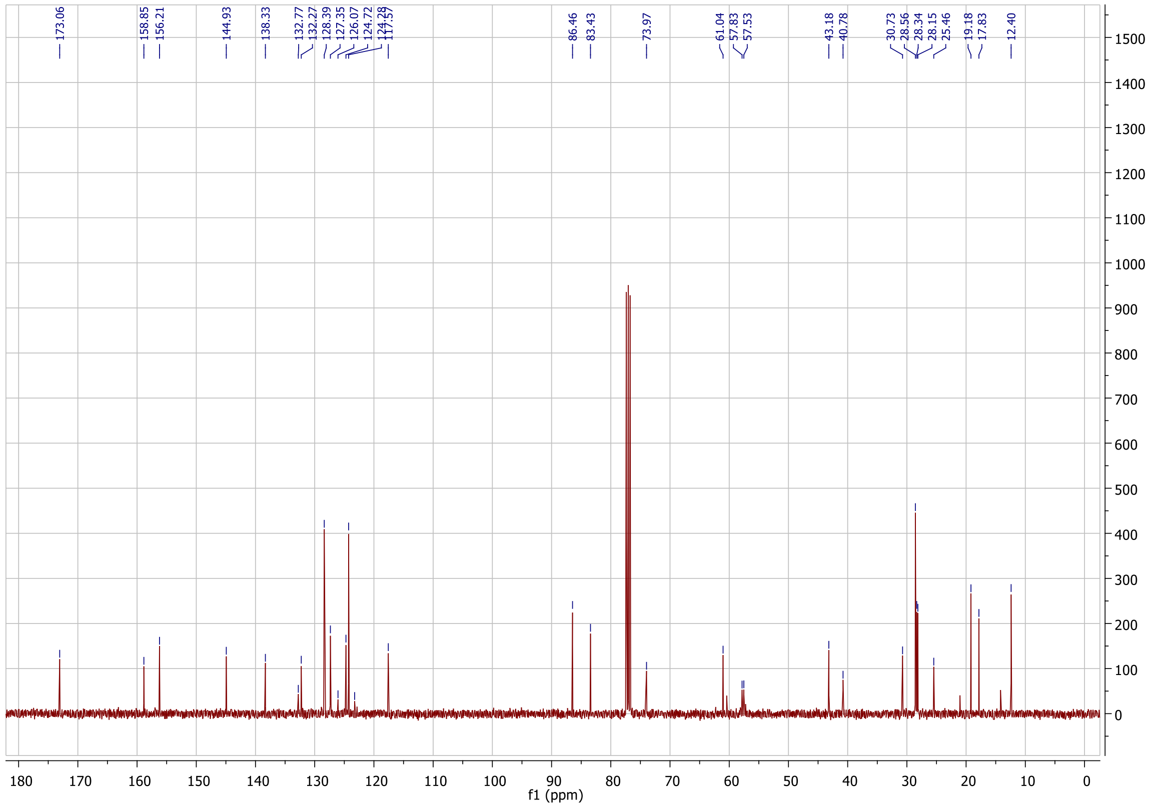

-L.G.S. - Laboratorio Grandi Strumenti - Display Report

Analysis Name	av cs80.d	Acquisition Date	10/09/19 11:31:27	Operator	Walter Panzeri
Sample Name		Method	Copy of _01tmix_posneg lm.MS	Instrument	esquire3000plus
Comment	1mg/ml dil 1:100 CH3CN<br>Richiedente: Sgorbati				

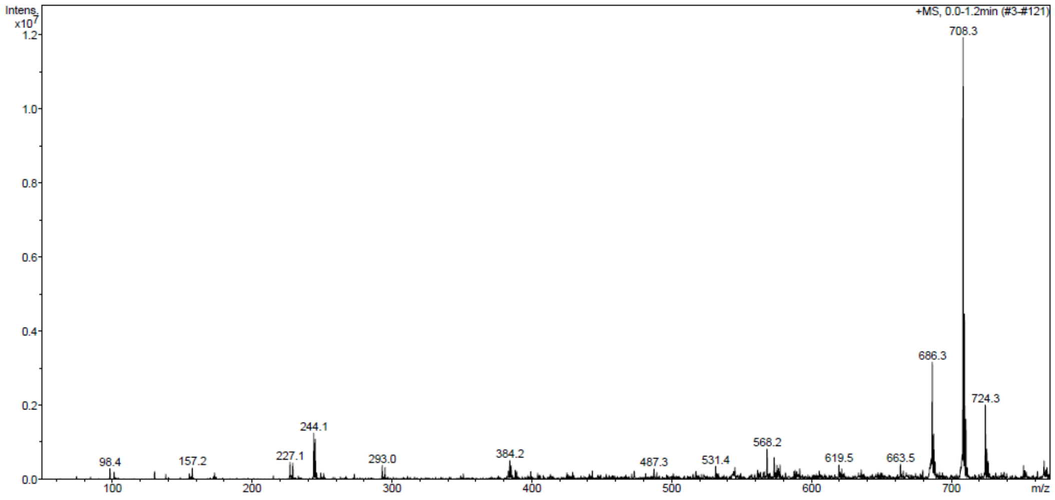

Bruker Daltonics DataAnalysis 3.1   printed: 10/09/19 11:32:56   Page 1 of 1

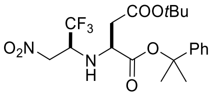

3h ^{1}H NMR (400 MHz, $CDCl_3$); ^{19}F NMR (376 MHz, $CDCl_3$)

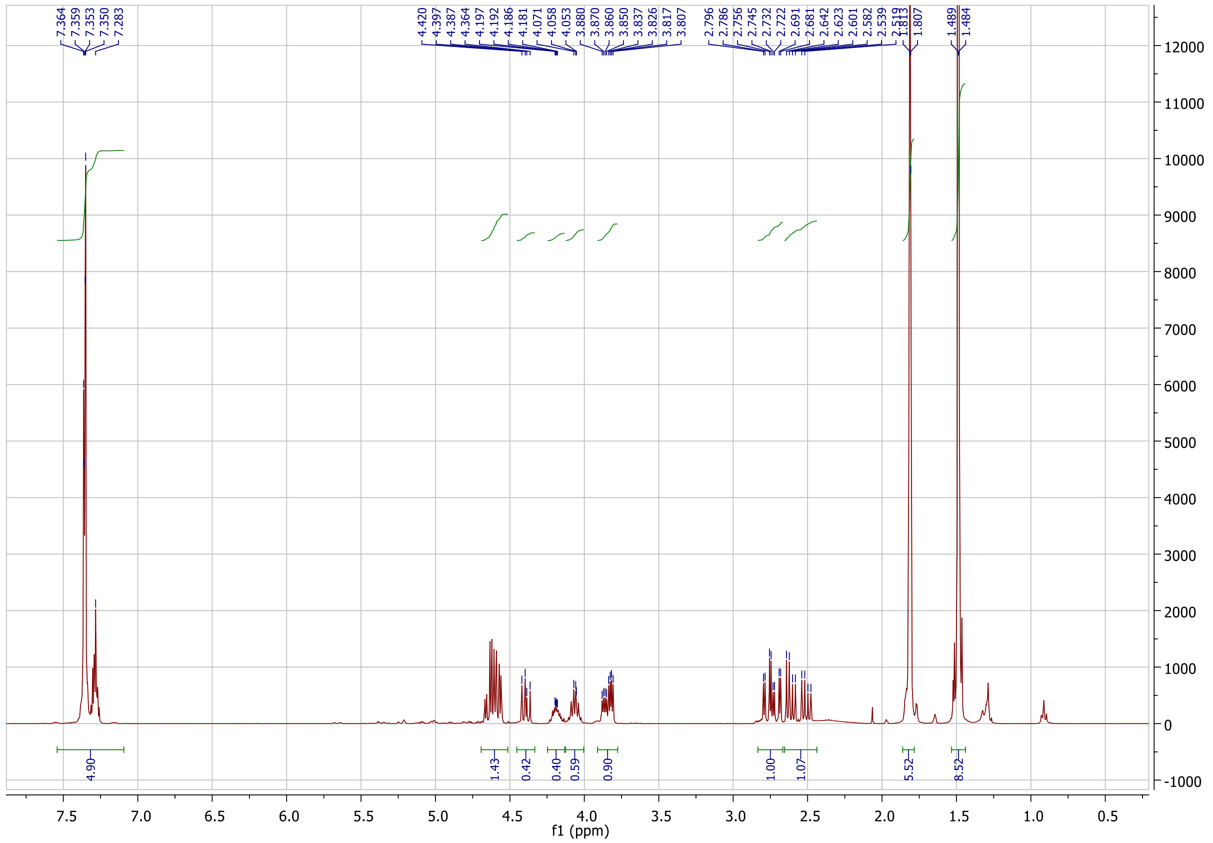

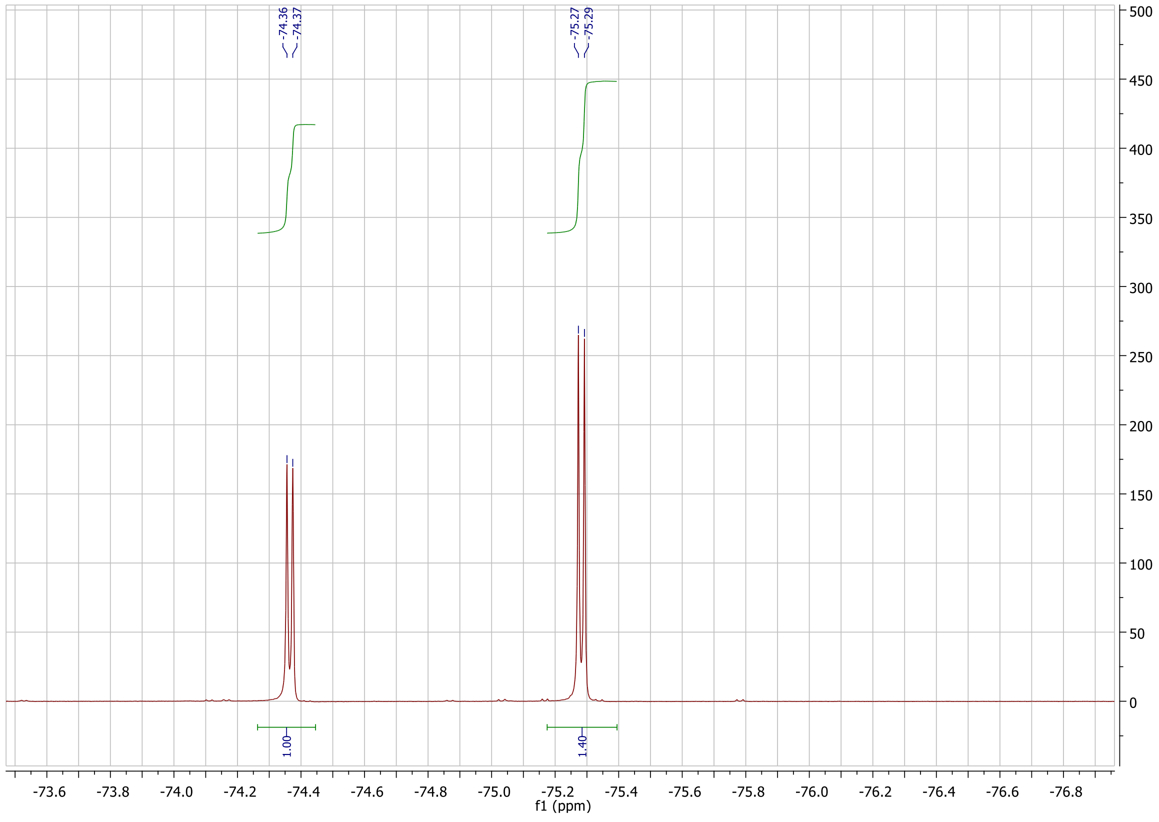

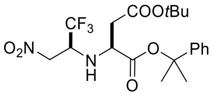

3h $^{13}C\{^{1}H\}$ NMR (101 MHz, $CDCl_3$)

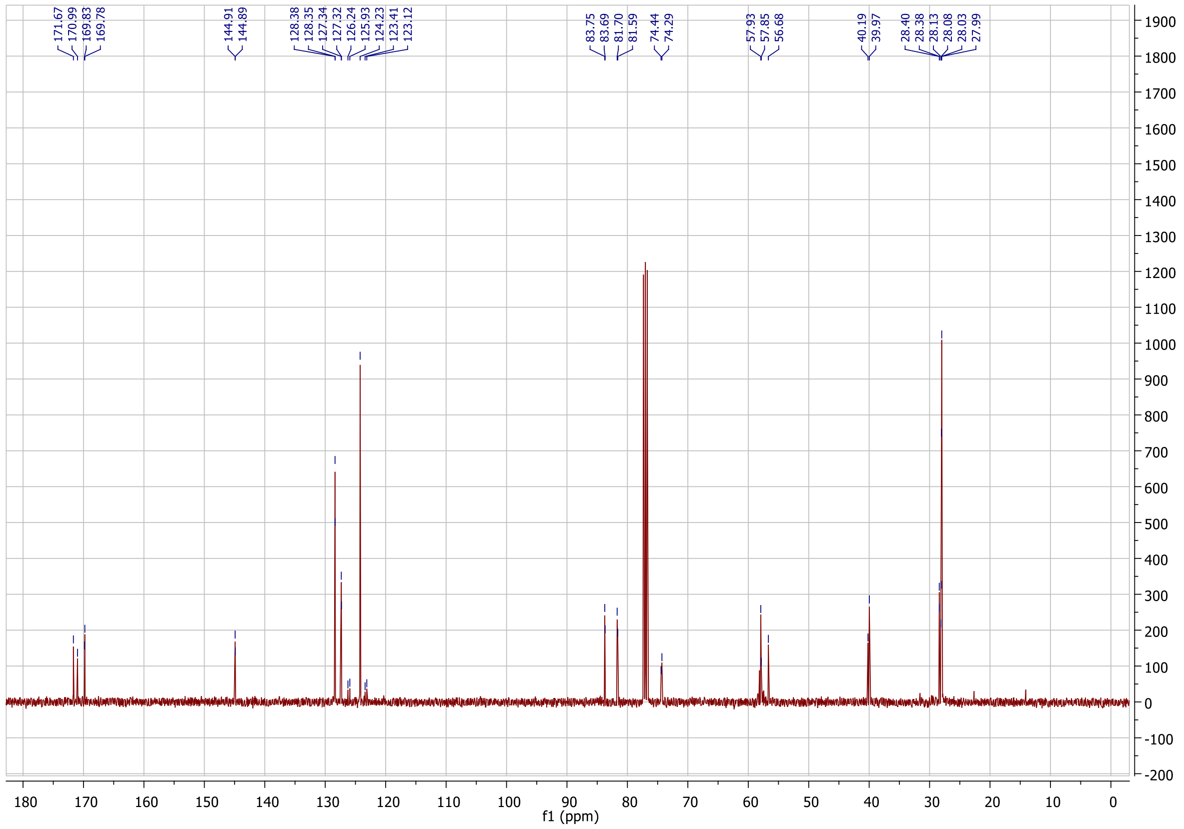

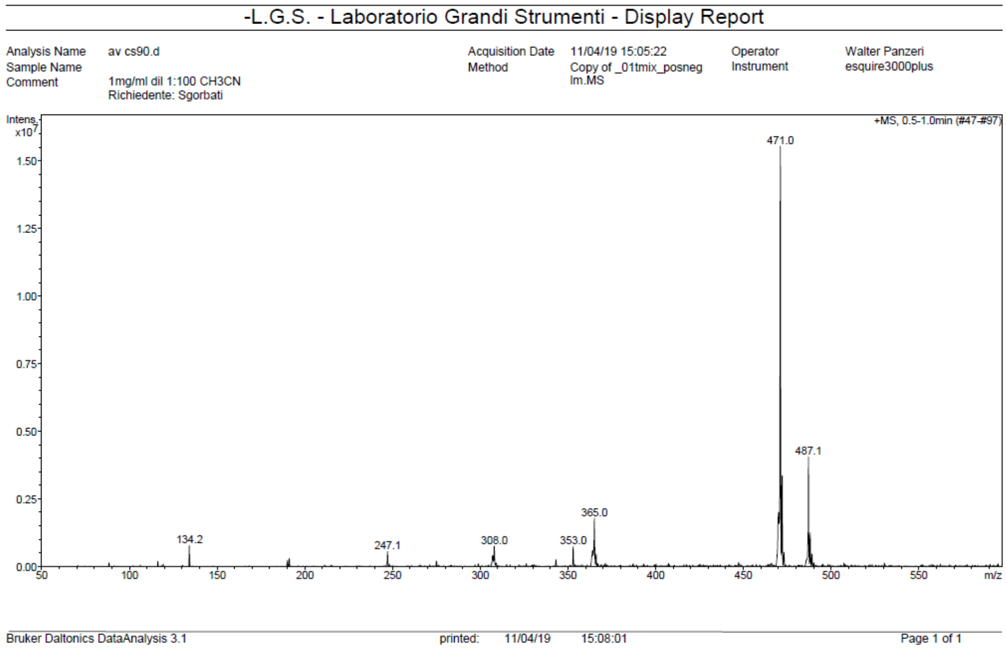

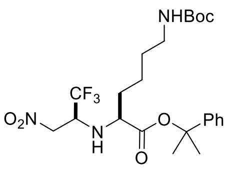

3i ^{1}H NMR (400 MHz, $CDCl_3$); ^{19}F NMR (376 MHz, $CDCl_3$)

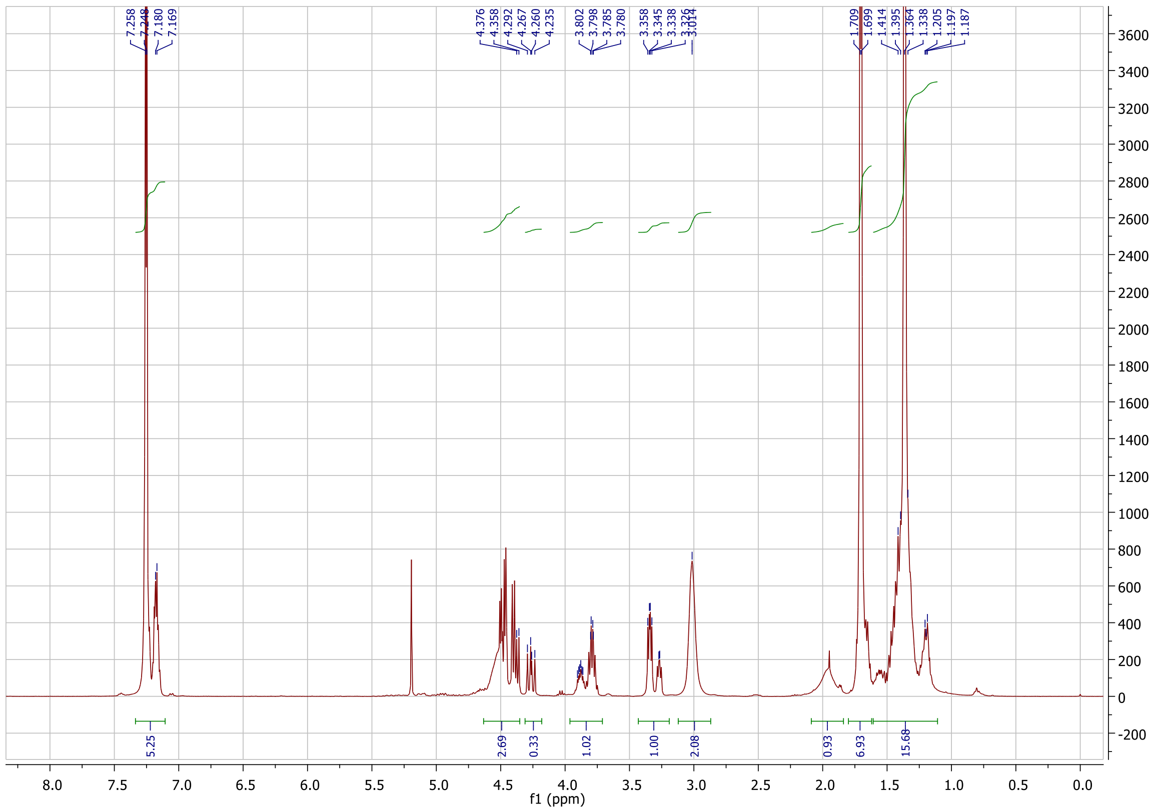

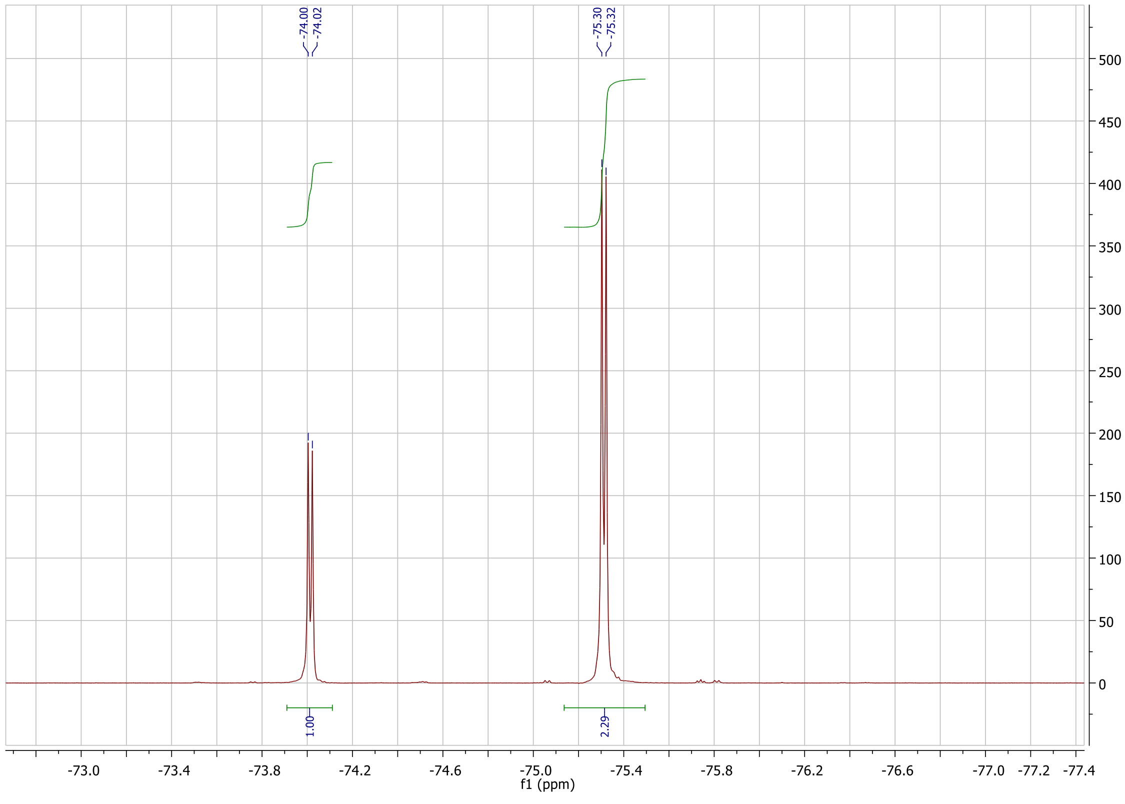

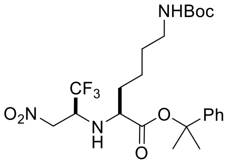

3i $^{13}C\{^{1}H\}$ NMR (101 MHz, $CDCl_3$)

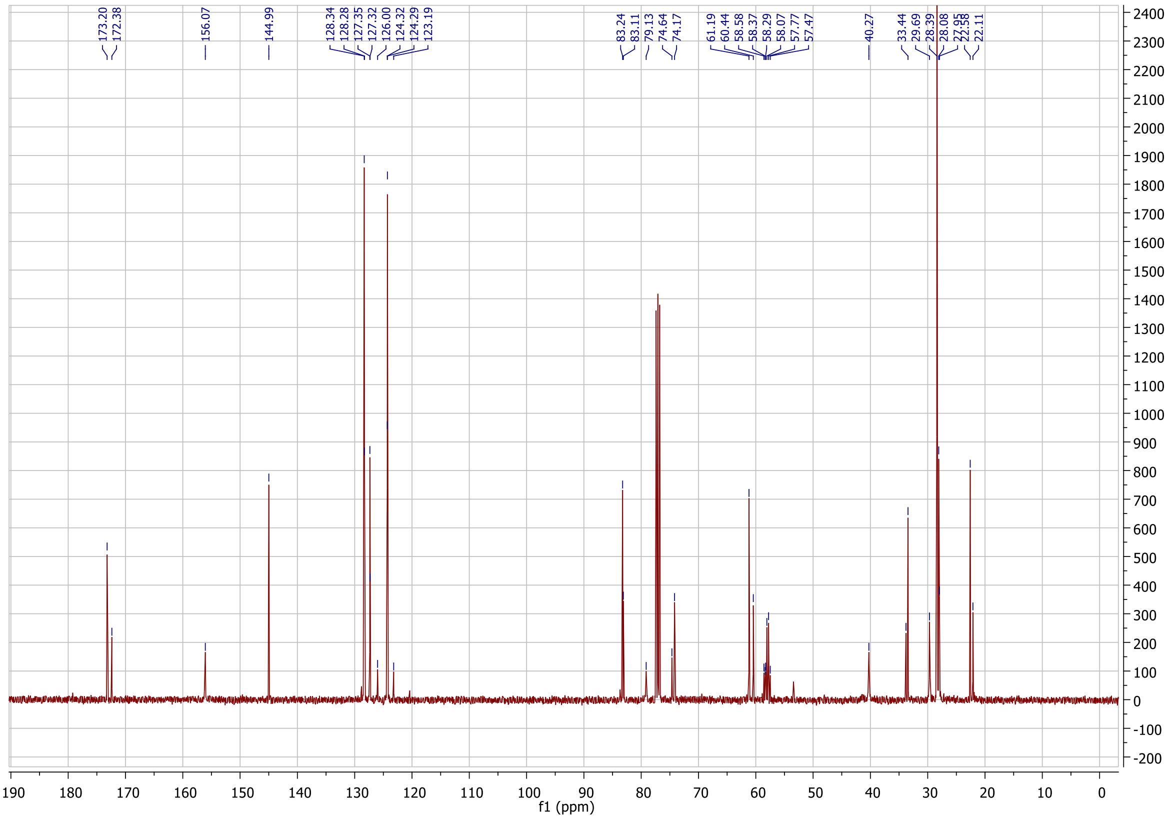

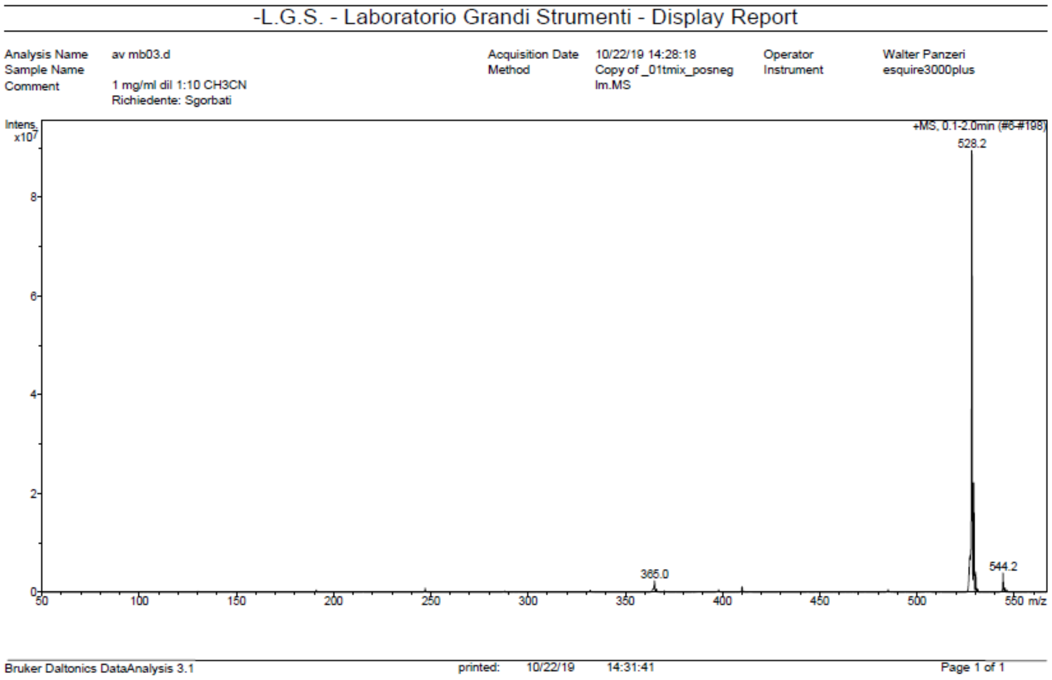

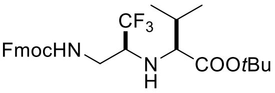

4a ^{1}H NMR (400 MHz, $CDCl_3$); ^{19}F NMR (376 MHz, $CDCl_3$)

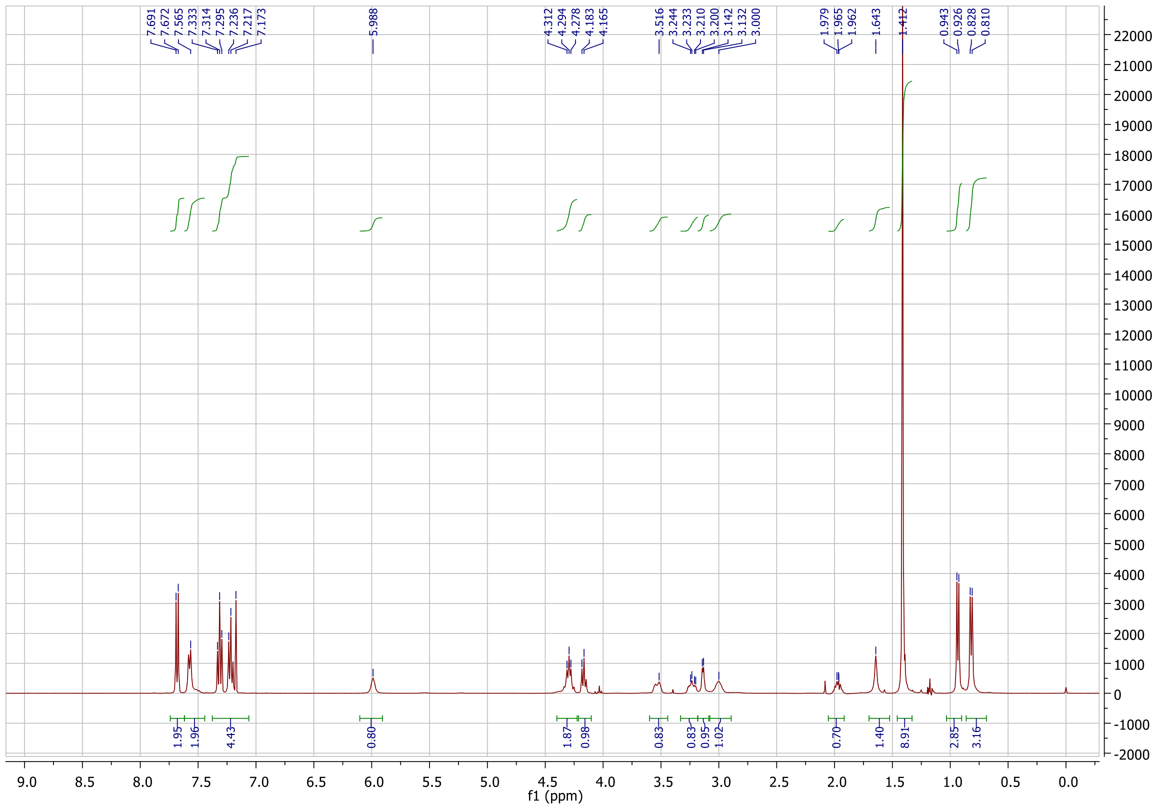

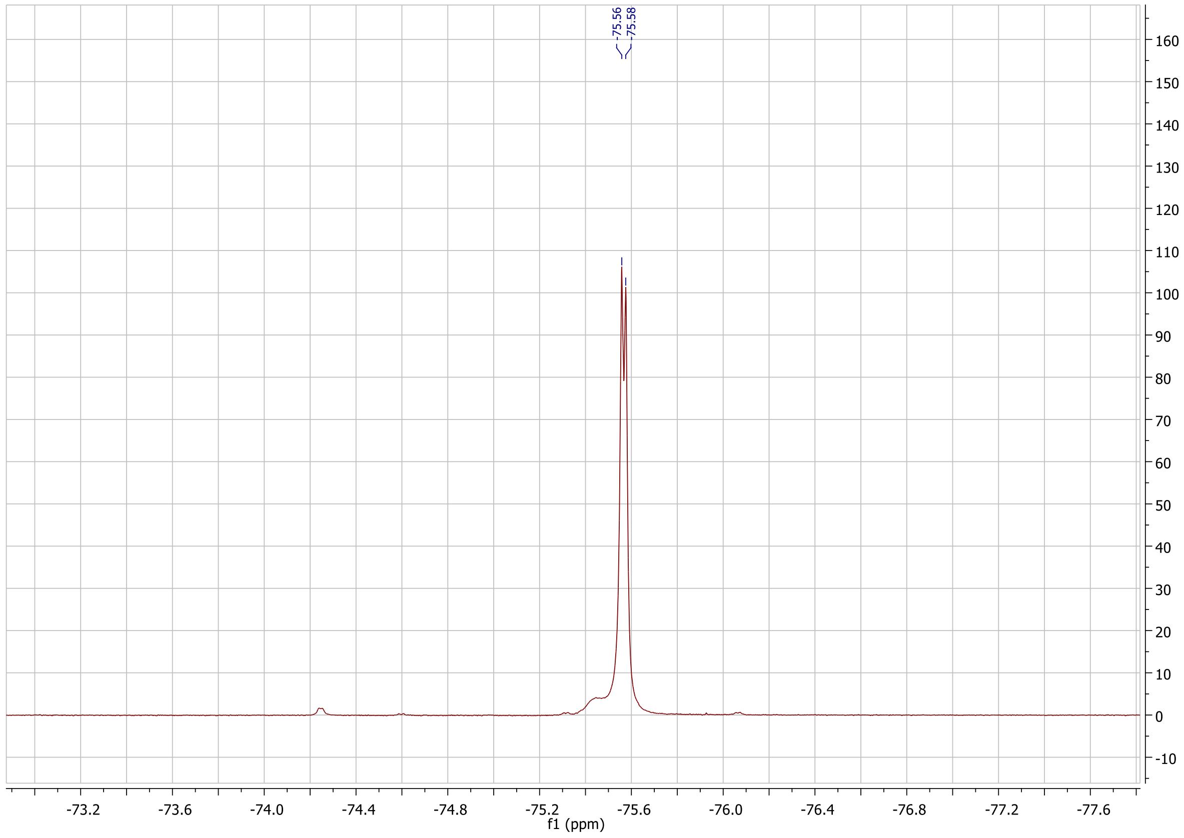

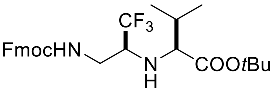

4a $^{13}C\{^{1}H\}$ NMR (101 MHz, $CDCl_3$)

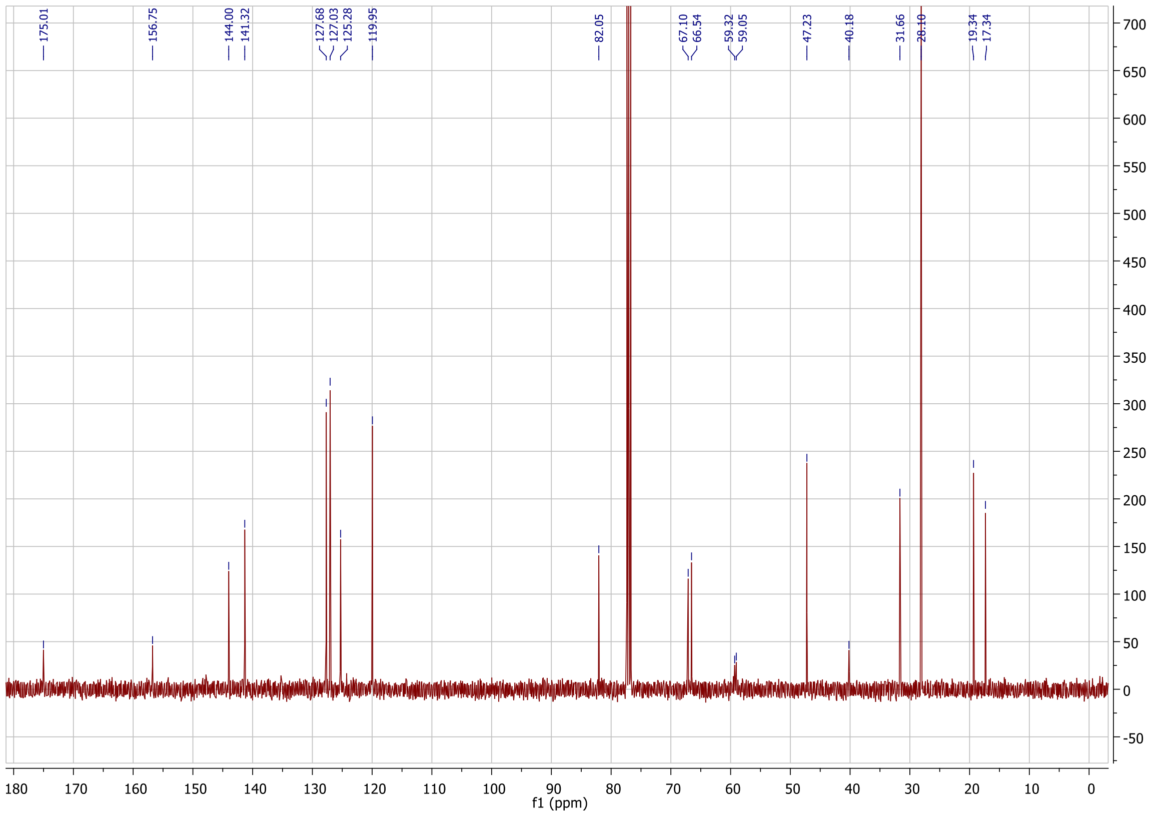

-L.G.S. - Laboratorio Grandi Strumenti - Display Report

Analysis Name	av cs_37.d	Acquisition Date	05/30/19 14:26:58	Operator	Walter Panzeri
Sample Name		Method	Copy of _01tmix_posneg lm.MS	Instrument	esquire3000plus
Comment	1mg/ml dil 1:100 MeOH<br>Richiedente: Volonterio				

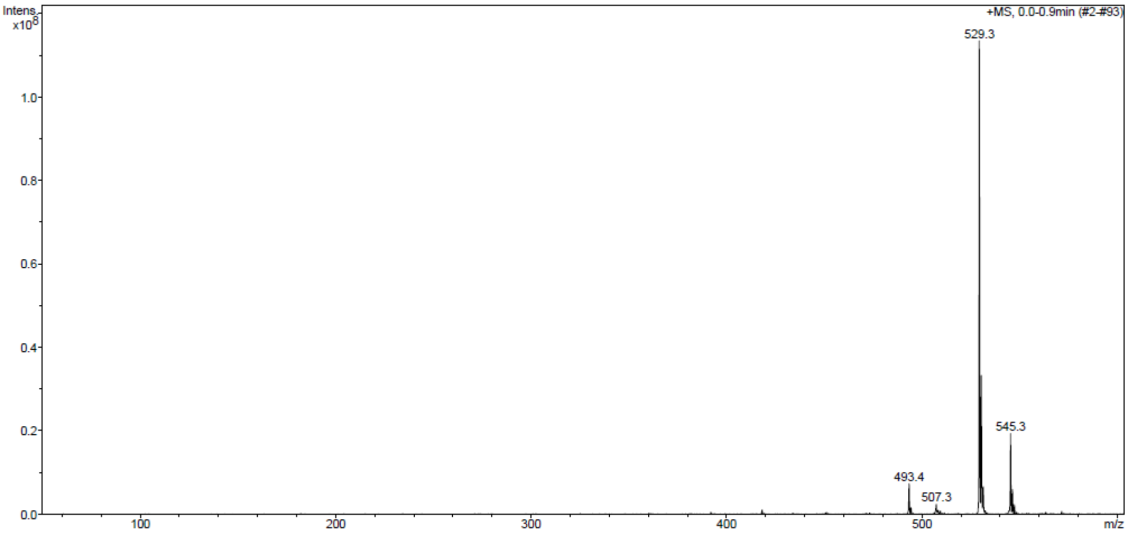

Bruker Daltonics DataAnalysis 3.1   printed: 05/30/19 15:39:03   Page 1 of 1

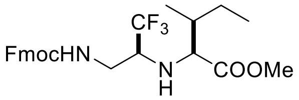

4b ^{1}H NMR (400 MHz, CD_3OD); ^{19}F NMR (376 MHz, CD_3OD)

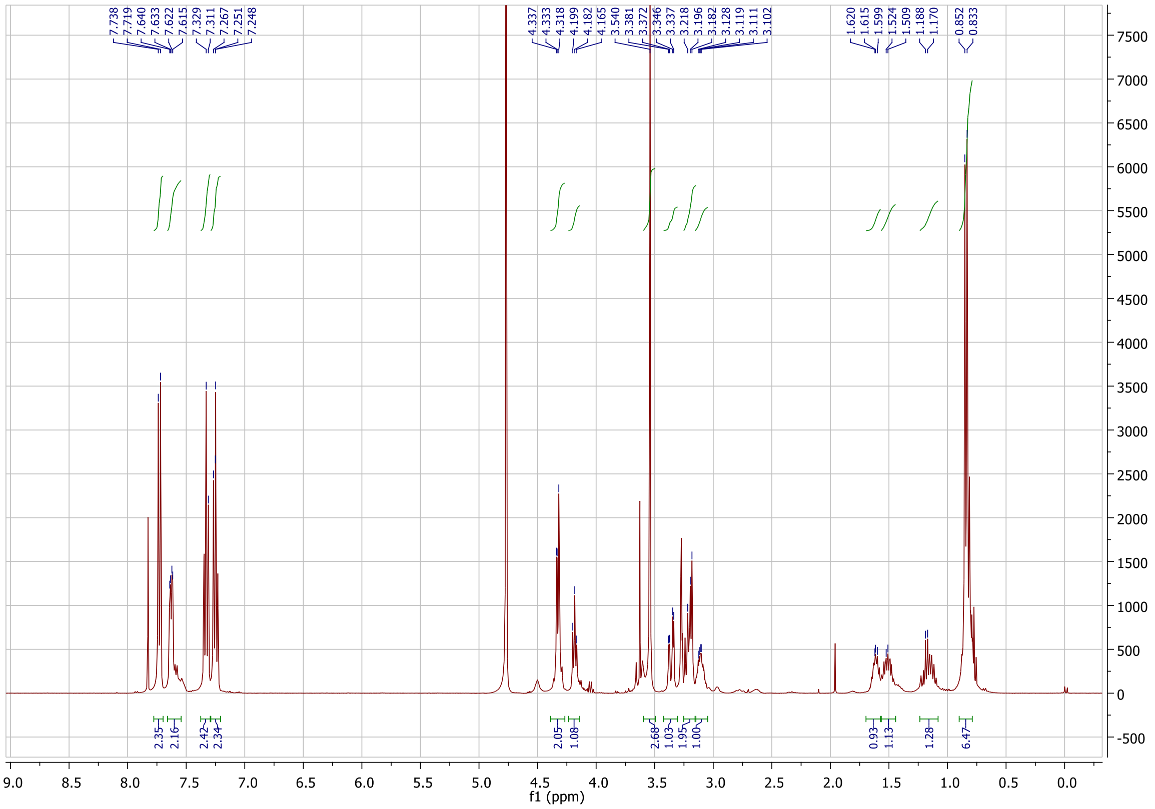

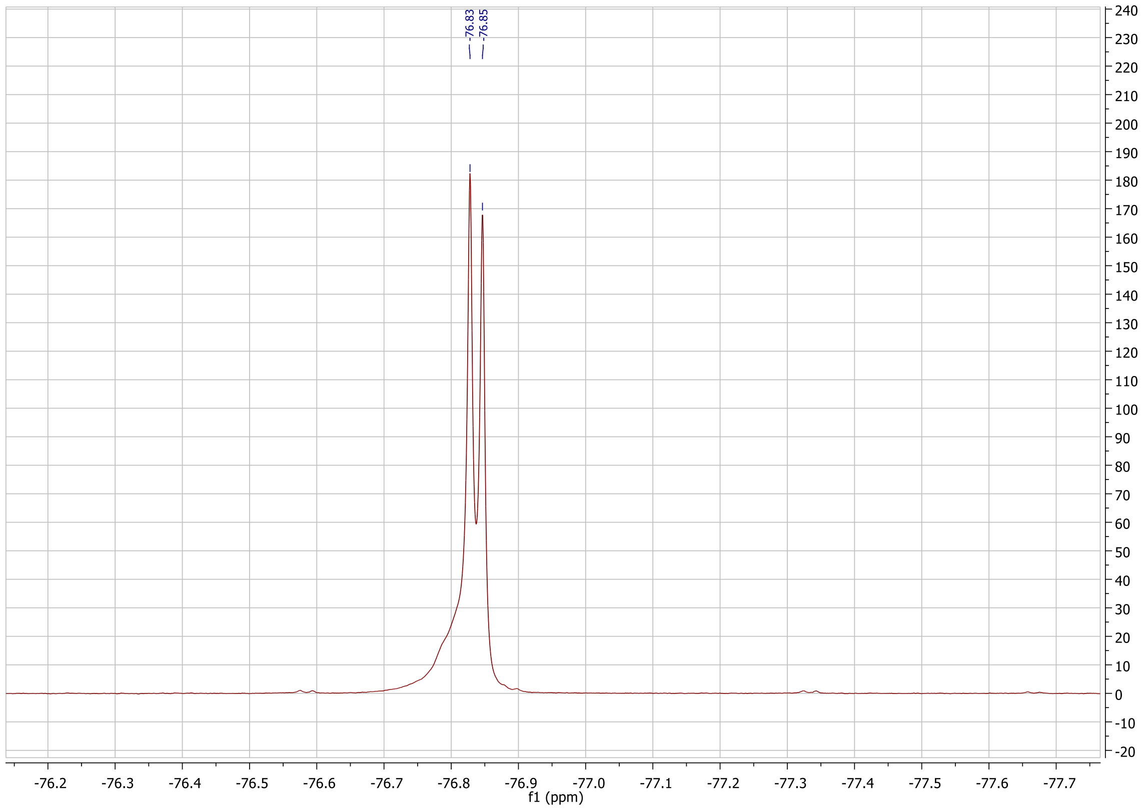

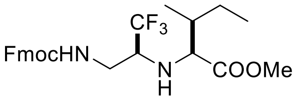

4b $^{13}C\{^{1}H\}$ NMR (101 MHz, CD_3OD)

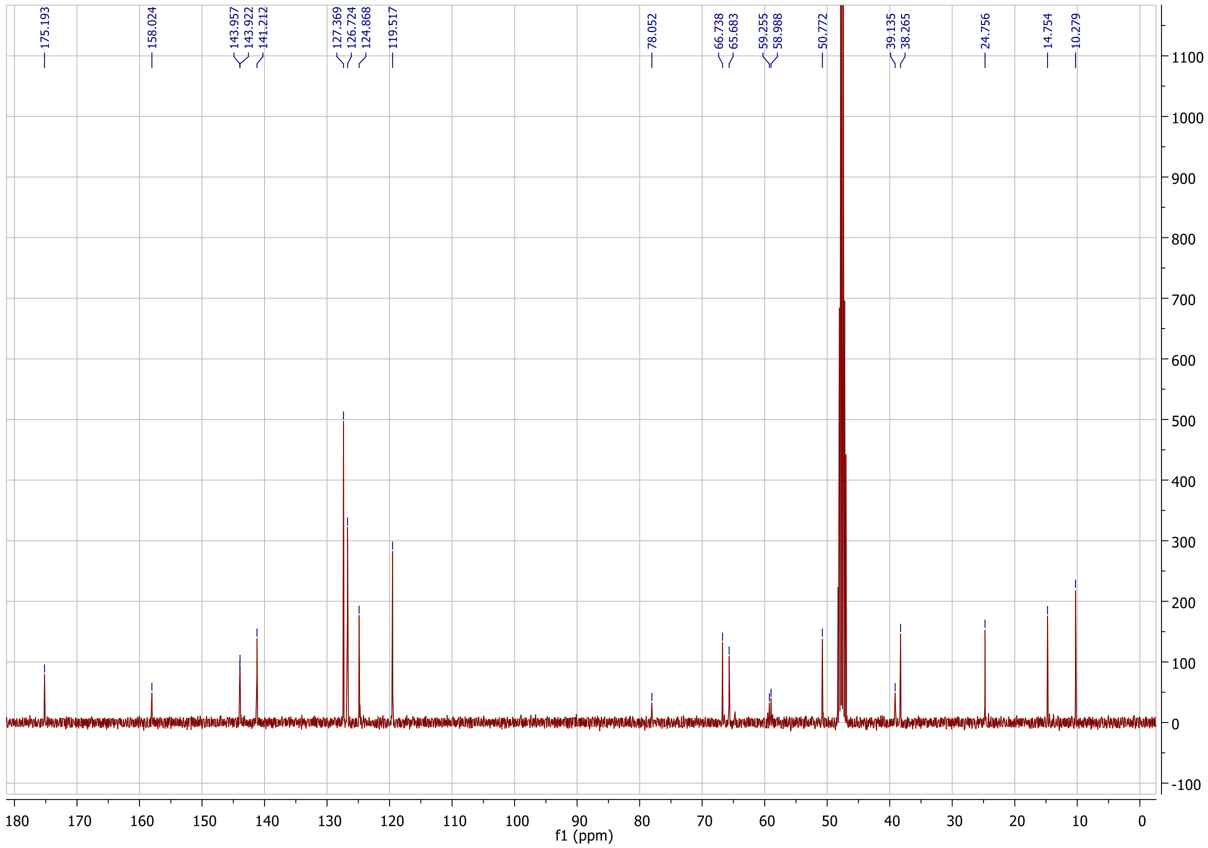

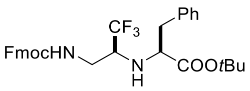

4c ^{1}H NMR (400 MHz, $CDCl_3$); ^{19}F NMR (376 MHz, $CDCl_3$)

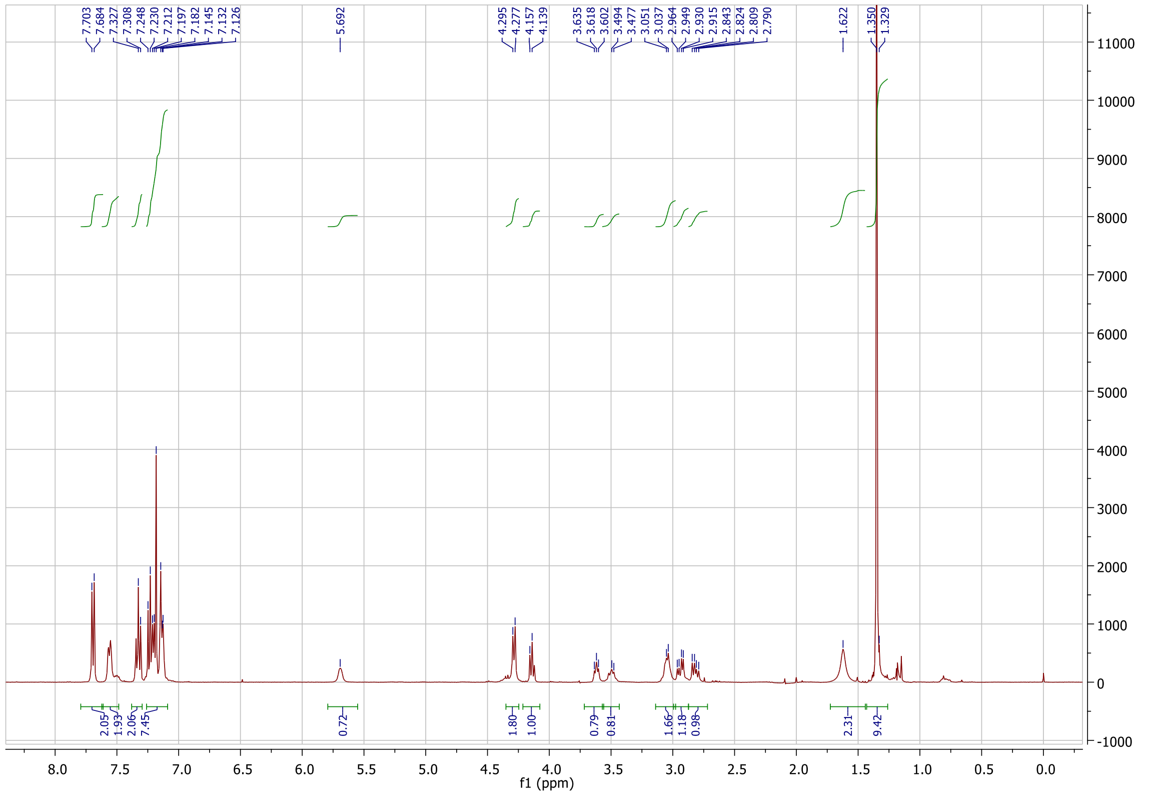

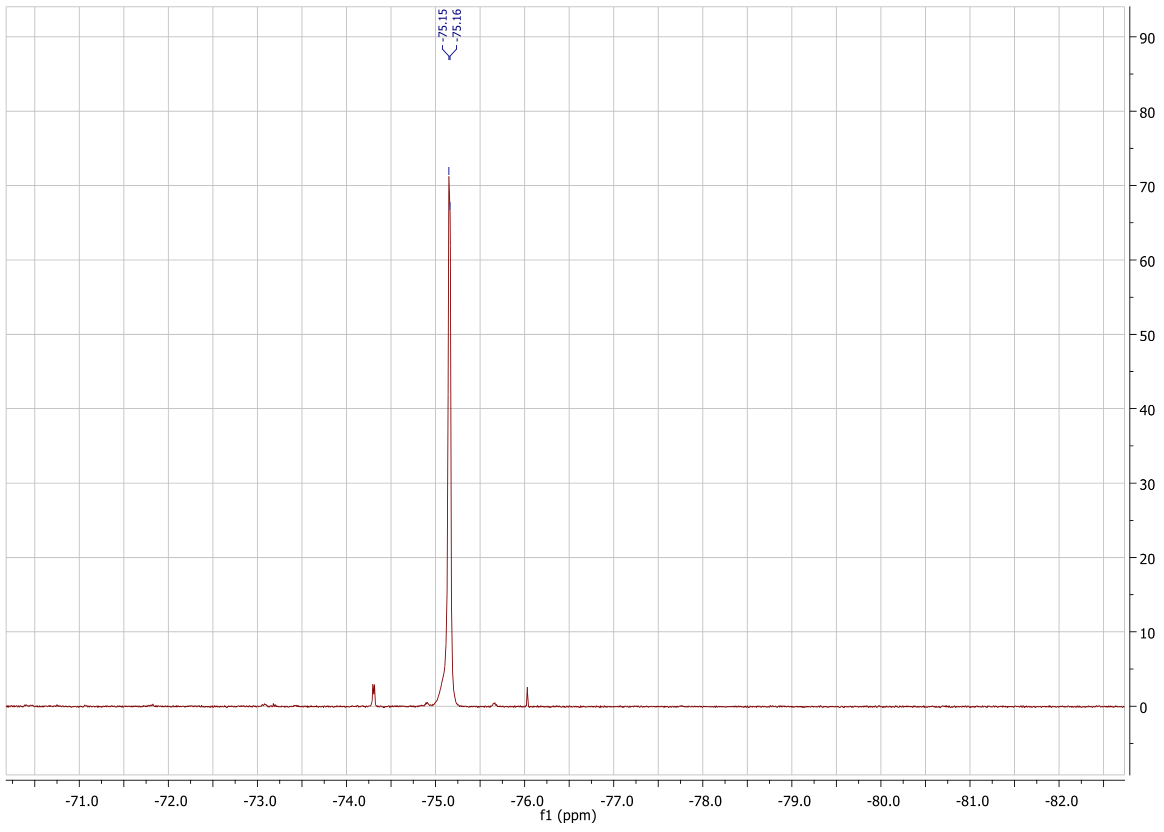

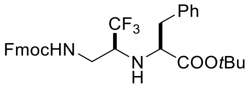

4c $^{13}C\{^1H\}$ NMR (101 MHz, $CDCl_3$)

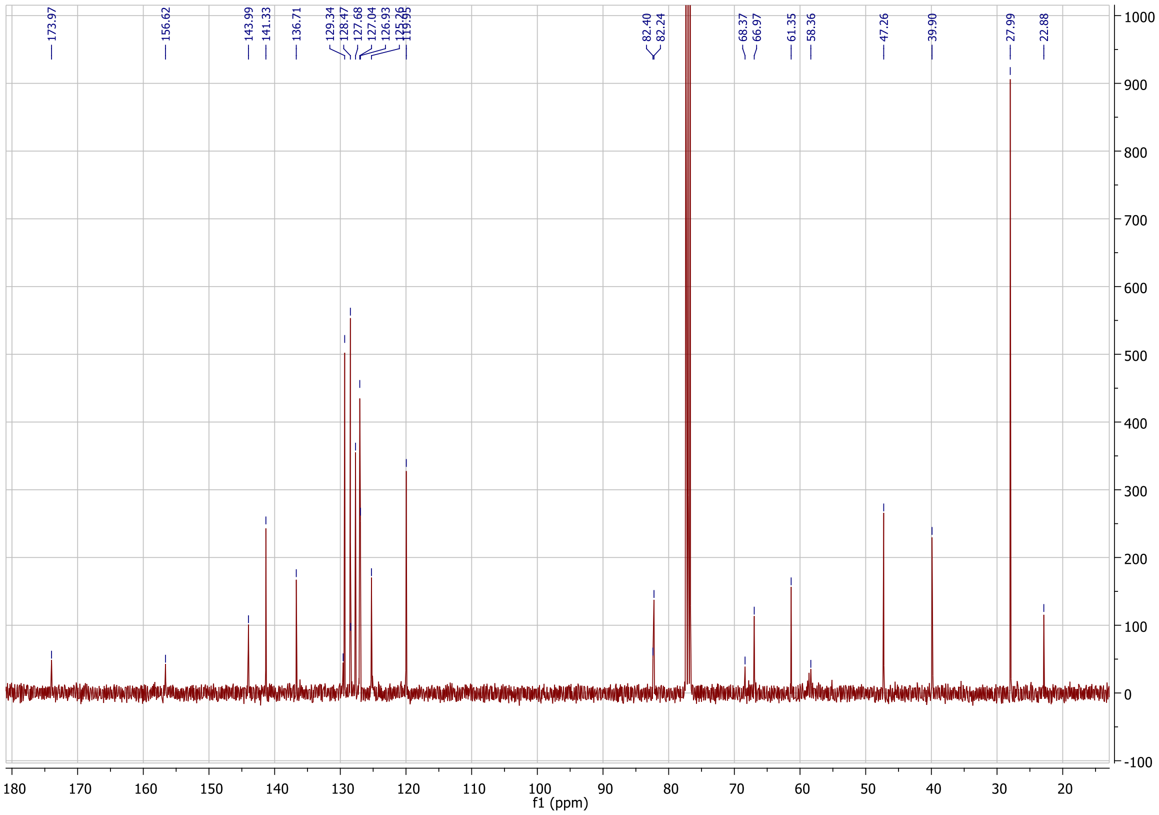

-L.G.S. - Laboratorio Grandi Strumenti - Display Report

Analysis Name	av cs45b.d	Acquisition Date	06/14/19 11:16:03	Operator	Walter Panzeri
Sample Name		Method	Copy of _01tmix_posneg lm.MS	Instrument	esquire3000plus
Comment	1mg/ml dil 1:100 MeOH Richiedente: Sgorbati				

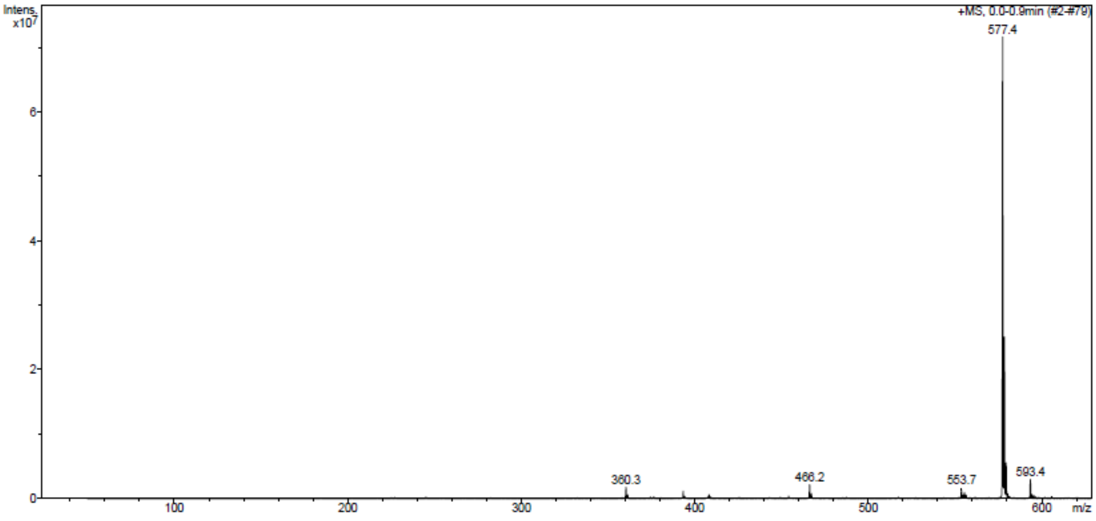

Bruker Daltonics DataAnalysis 3.1 printed: 06/14/19 11:17:45 Page 1 of 1

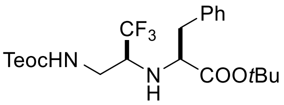

4d ^{1}H NMR (400 MHz, $CDCl_3$); ^{19}F NMR (376 MHz, $CDCl_3$)

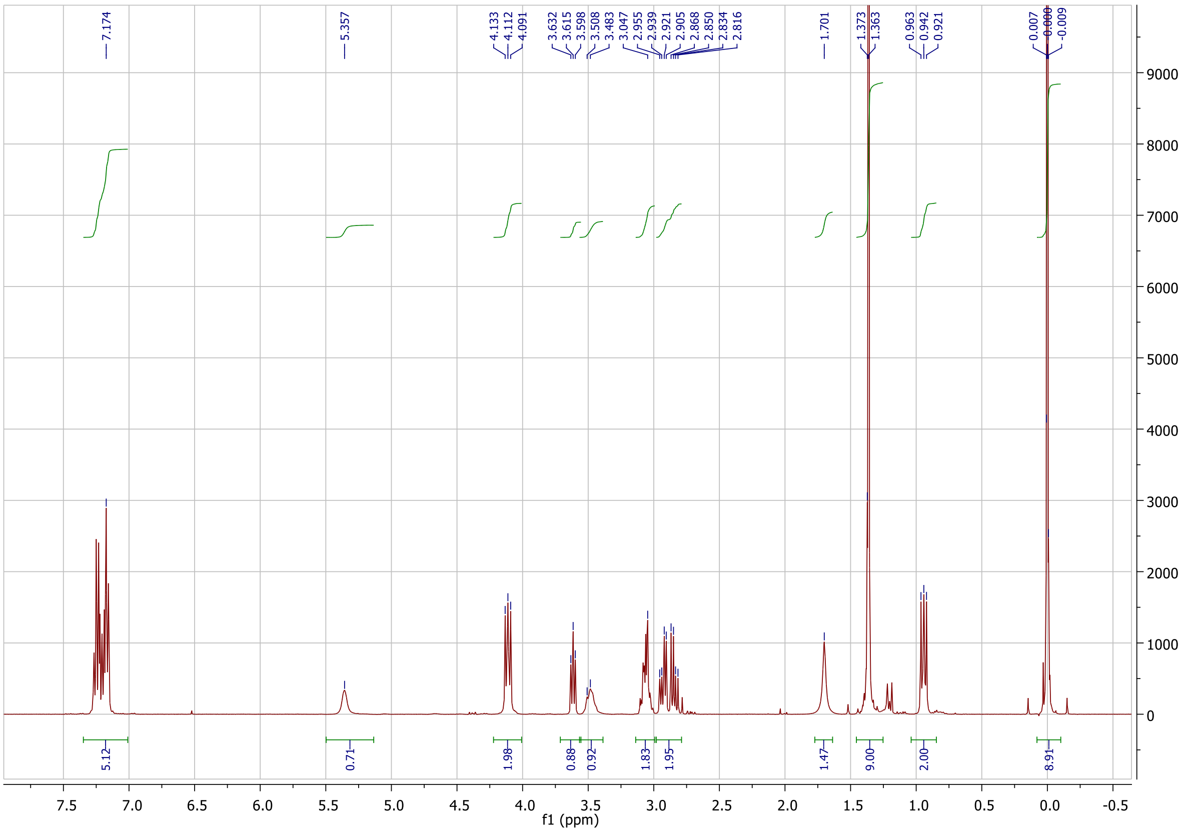

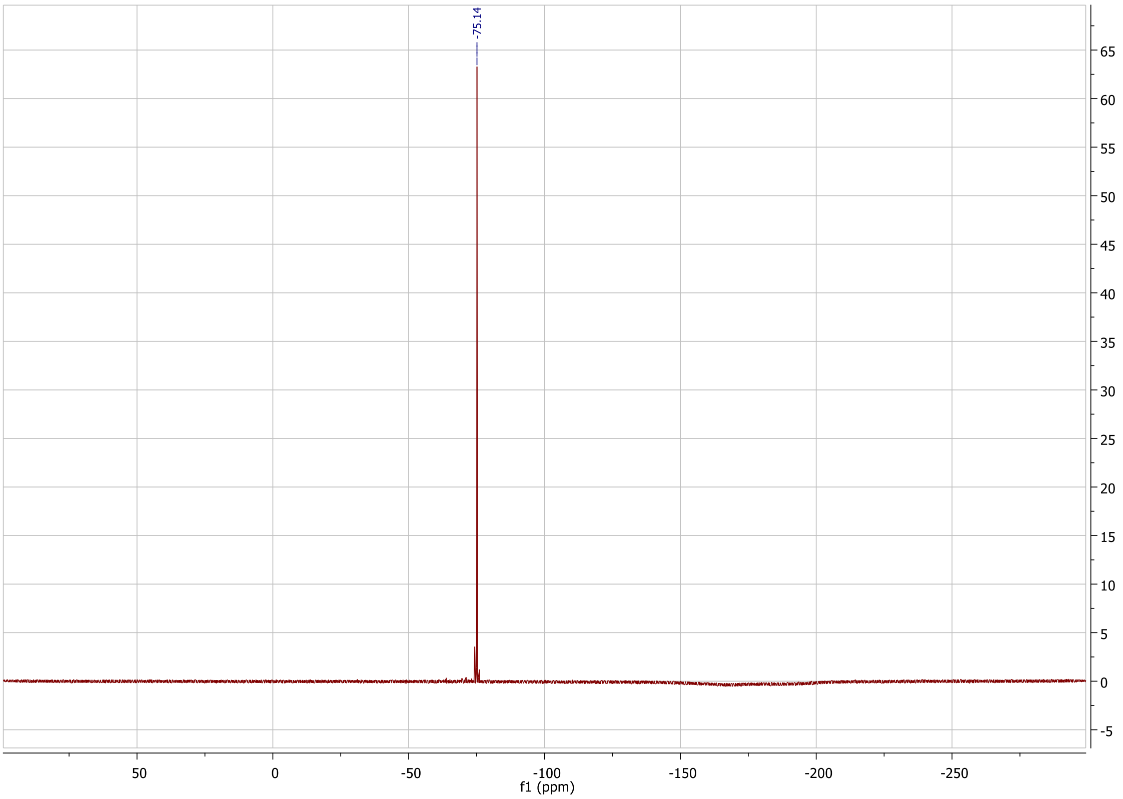

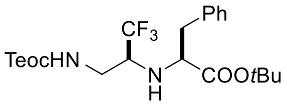

4d $^{13}C\{^{1}H\}$ NMR (101 MHz, $CDCl_3$)

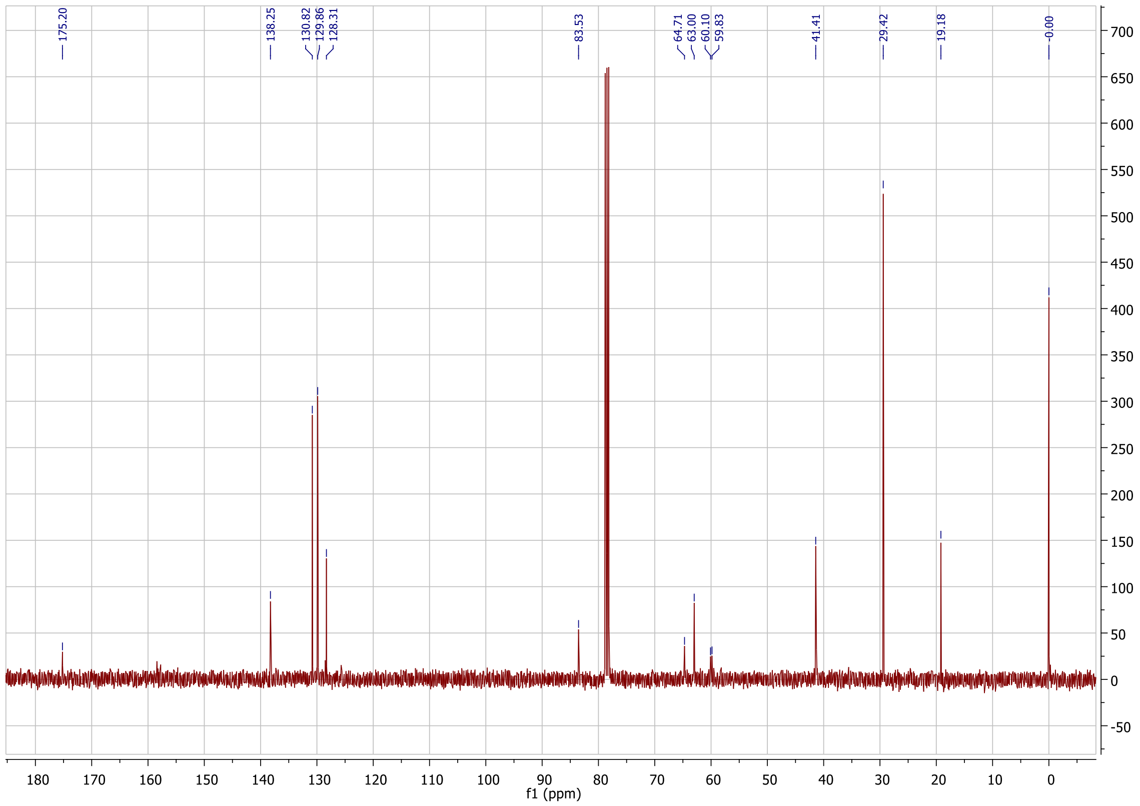

-L.G.S. - Laboratorio Grandi Strumenti - Display Report

Analysis Name	av cs65.d	Acquisition Date	11/06/19 14:39:42	Operator	Walter Panzeri
Sample Name		Method	Copy of _01tmix_posneg lm.MS	Instrument	esquire3000plus
Comment	1mg/ml dil 1:100 CH3CN Richiedente: Sgorbati				

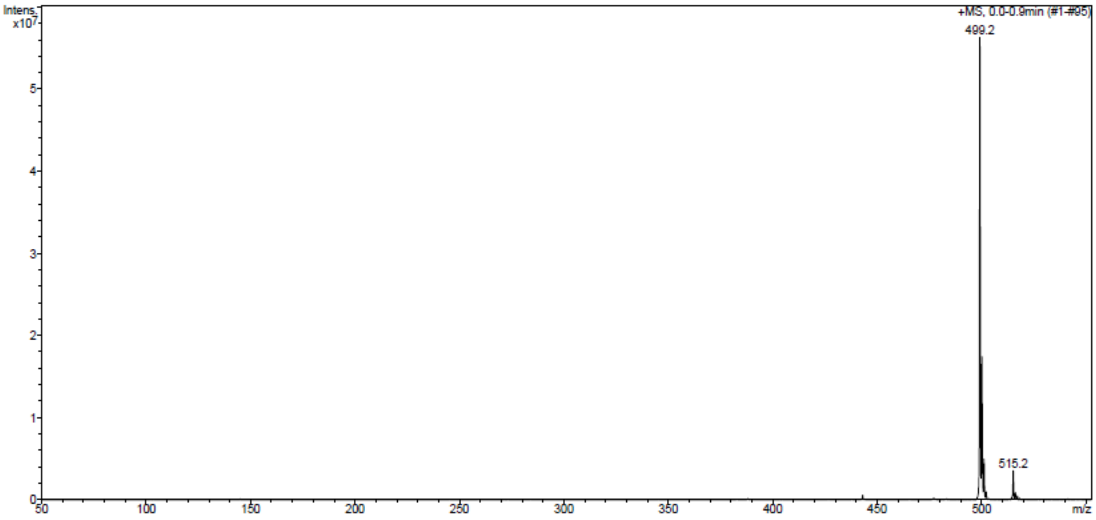

Bruker Daltonics DataAnalysis 3.1 printed: 11/06/19 14:40:55 Page 1 of 1

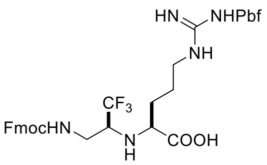

4e ^{1}H NMR (400 MHz, CD_3OD); ^{19}F NMR (376 MHz, CD_3OD)

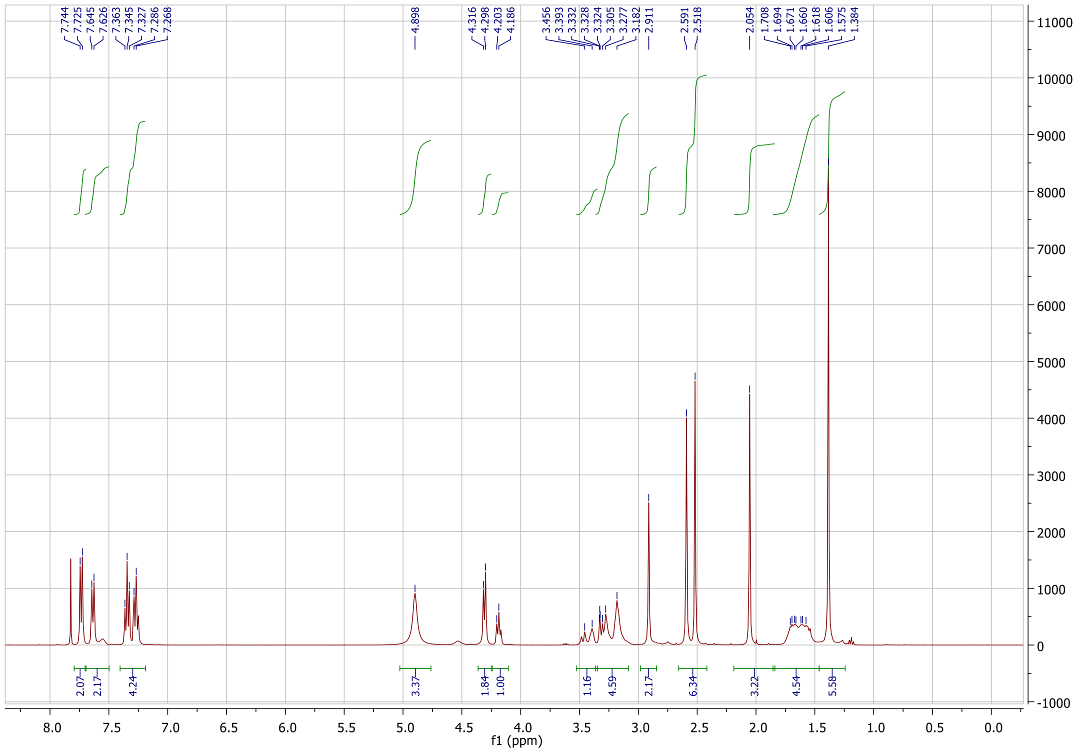

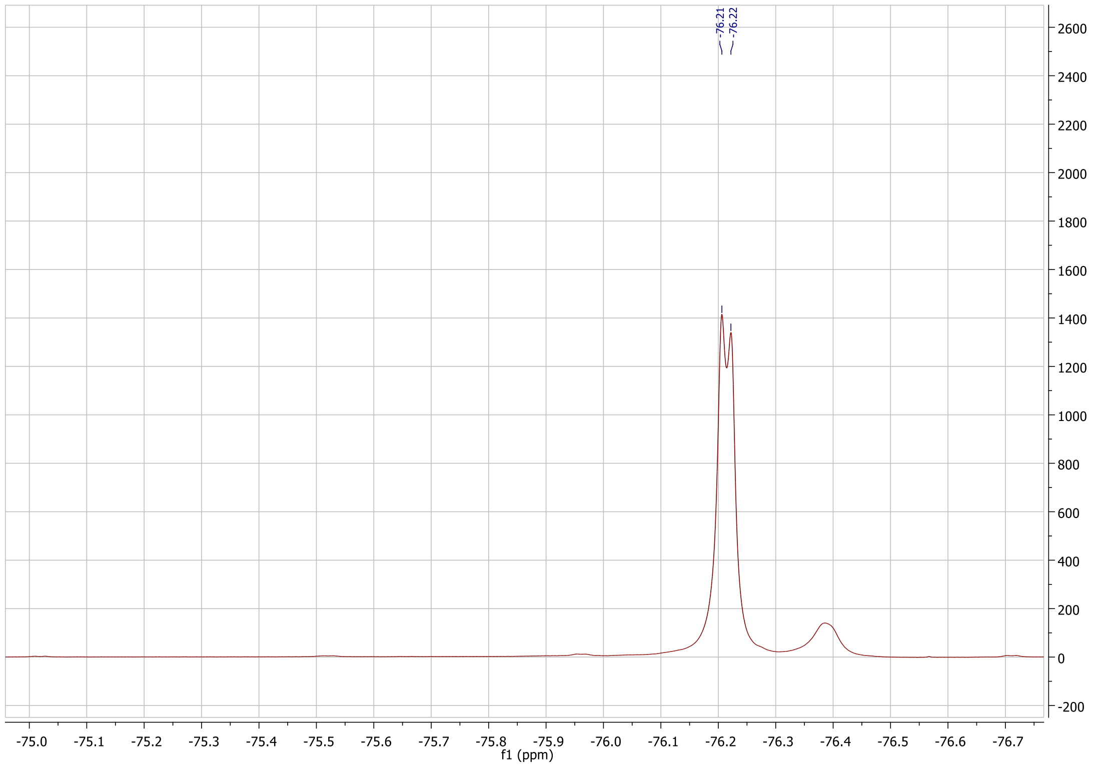

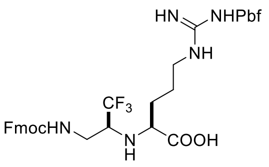

4e $^{13}C\{^{1}H\}$ NMR (101 MHz, CD_3OD)

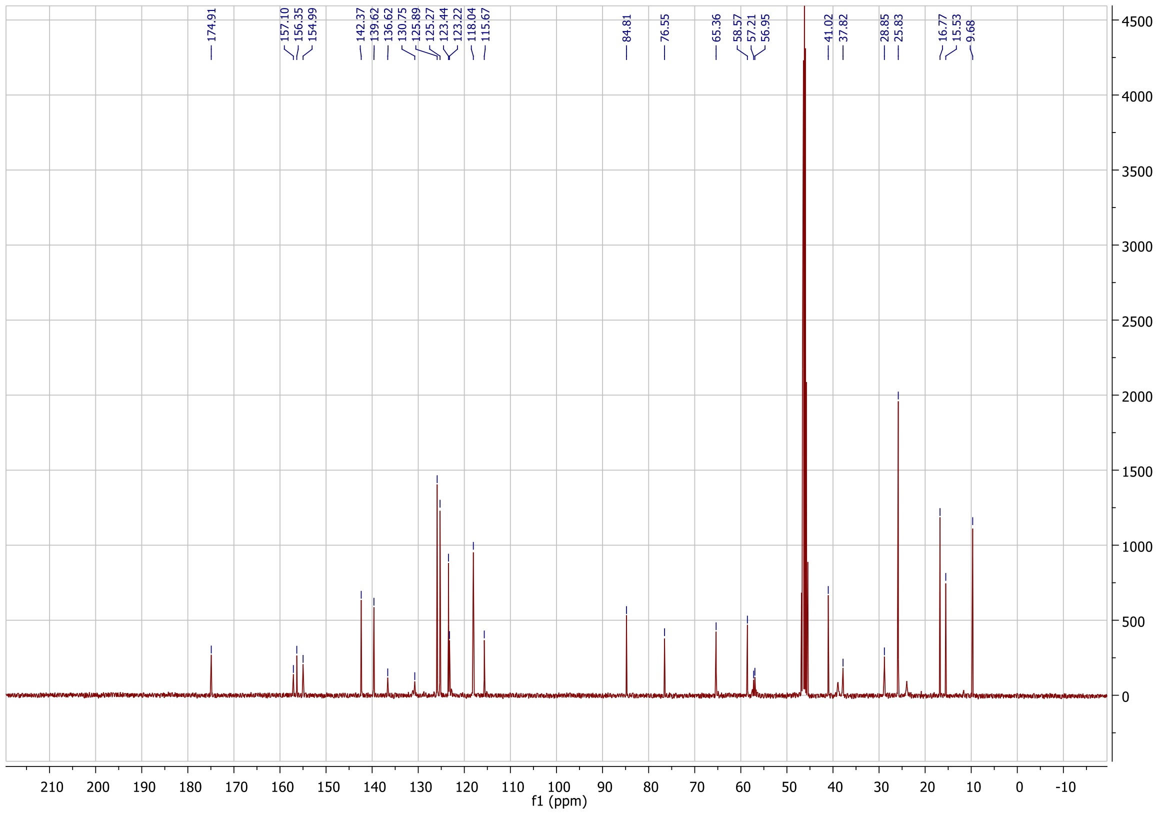

-L.G.S. - Laboratorio Grandi Strumenti - Display Report

Analysis Name	av cs82.d	Acquisition Date	10/18/19 10:16:46	Operator	Walter Panzeri
Sample Name		Method	Copy of _01tmix_posneg lm.MS	Instrument	esquire3000plus
Comment	1mg/ml dil 1:100 CH3CN Richiedente: Sgorbati				

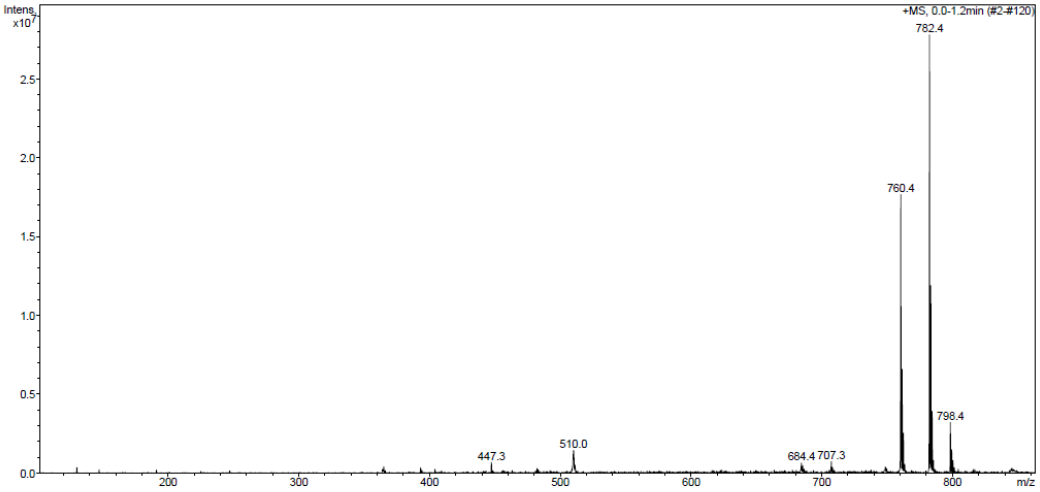

Bruker Daltonics DataAnalysis 3.1 printed: 10/18/19 10:18:20 Page 1 of 1

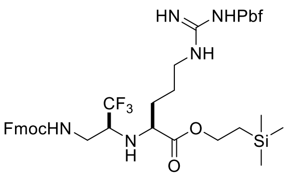

4f ^{1}H NMR (400 MHz, CD_3OD); ^{19}F NMR (376 MHz, CD_3OD)

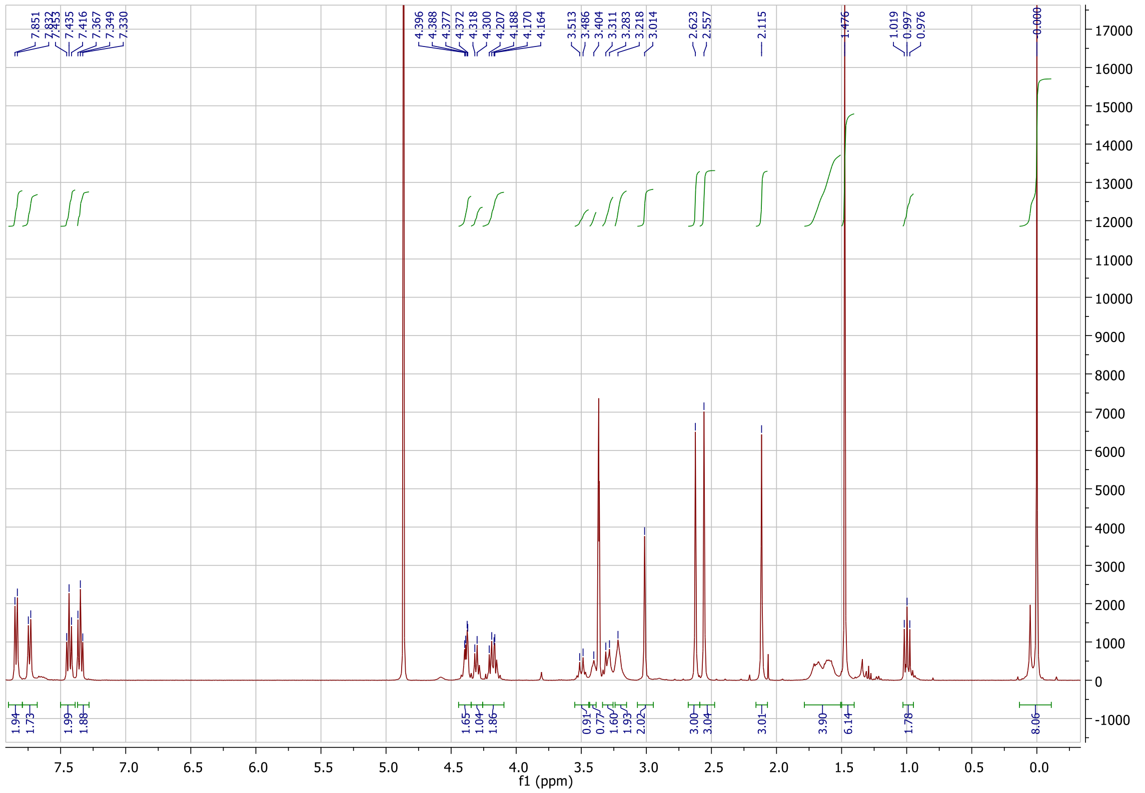

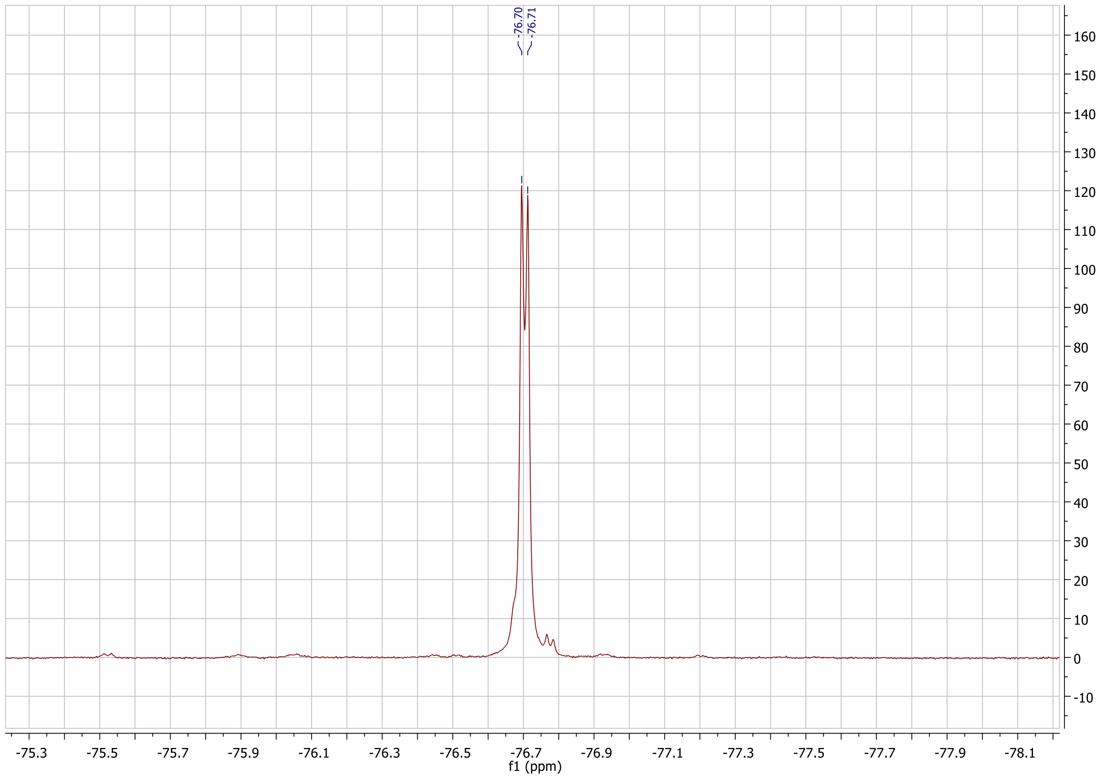

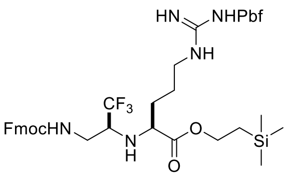

4f $^{13}C\{^{1}H\}$ NMR (101 MHz, CD_3OD)

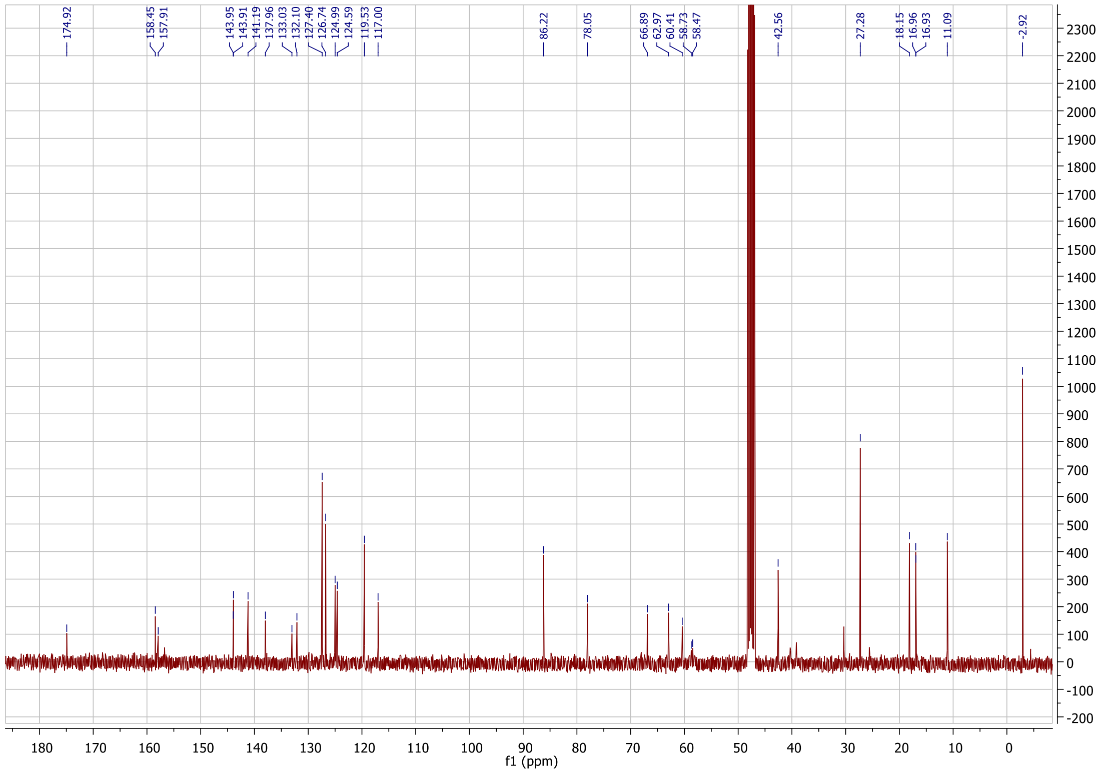

-L.G.S. - Laboratorio Grandi Strumenti - Display Report

Analysis Name	av cs07b.d	Acquisition Date	03/20/19 14:58:35	Operator	Walter Panzeri
Sample Name		Method	Copy of _01tmix_posneg lm.MS	Instrument	esquire3000plus
Comment	1 mg/ml dil 1:100 MeOH Richiedente: Volonterio				

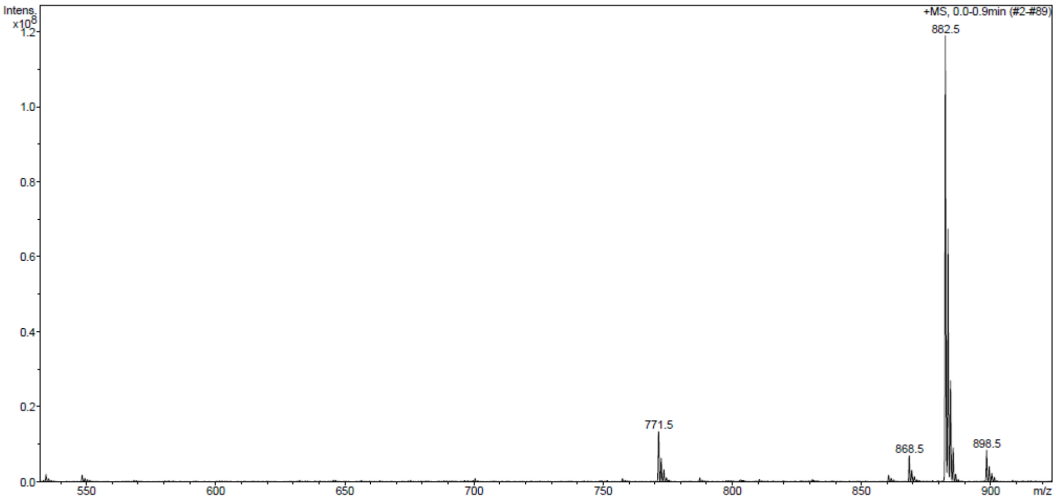

Bruker Daltonics DataAnalysis 3.1 printed: 03/20/19 15:02:28 Page 1 of 1

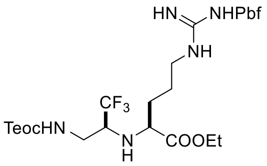

4g ^{1}H NMR (400 MHz, $CDCl_3$); ^{19}F NMR (376 MHz, $CDCl_3$)

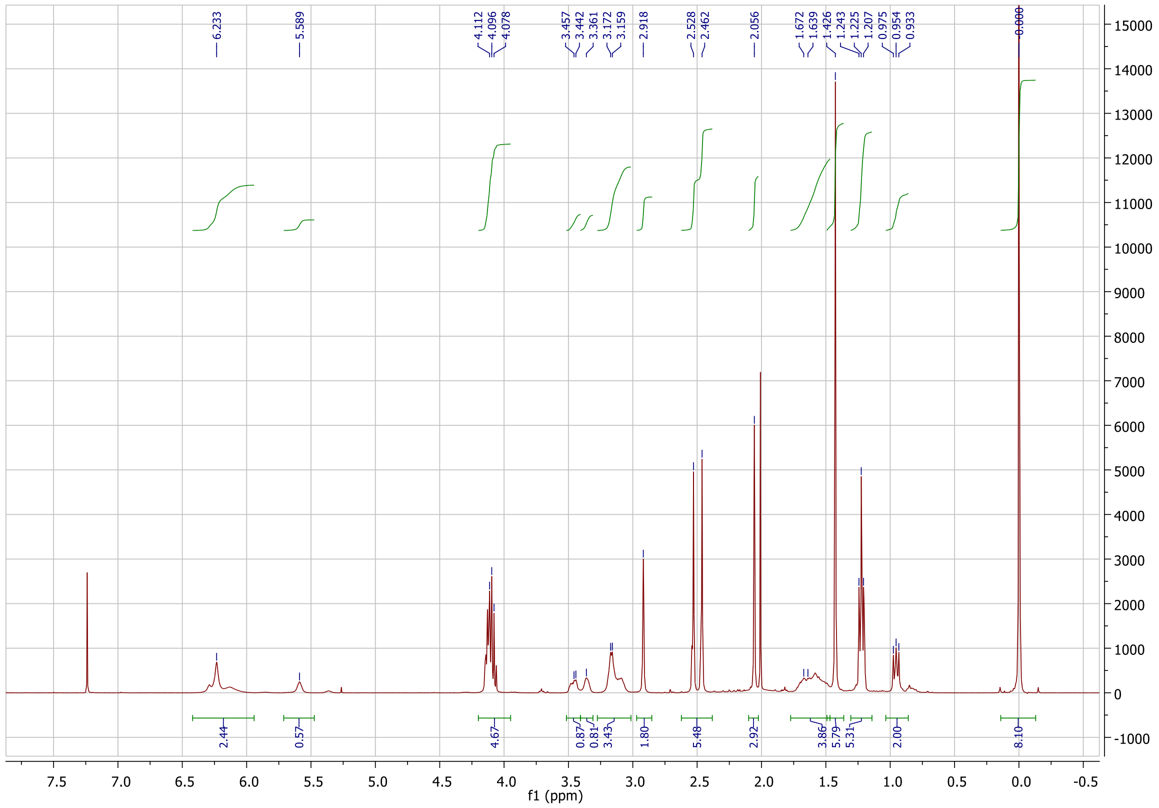

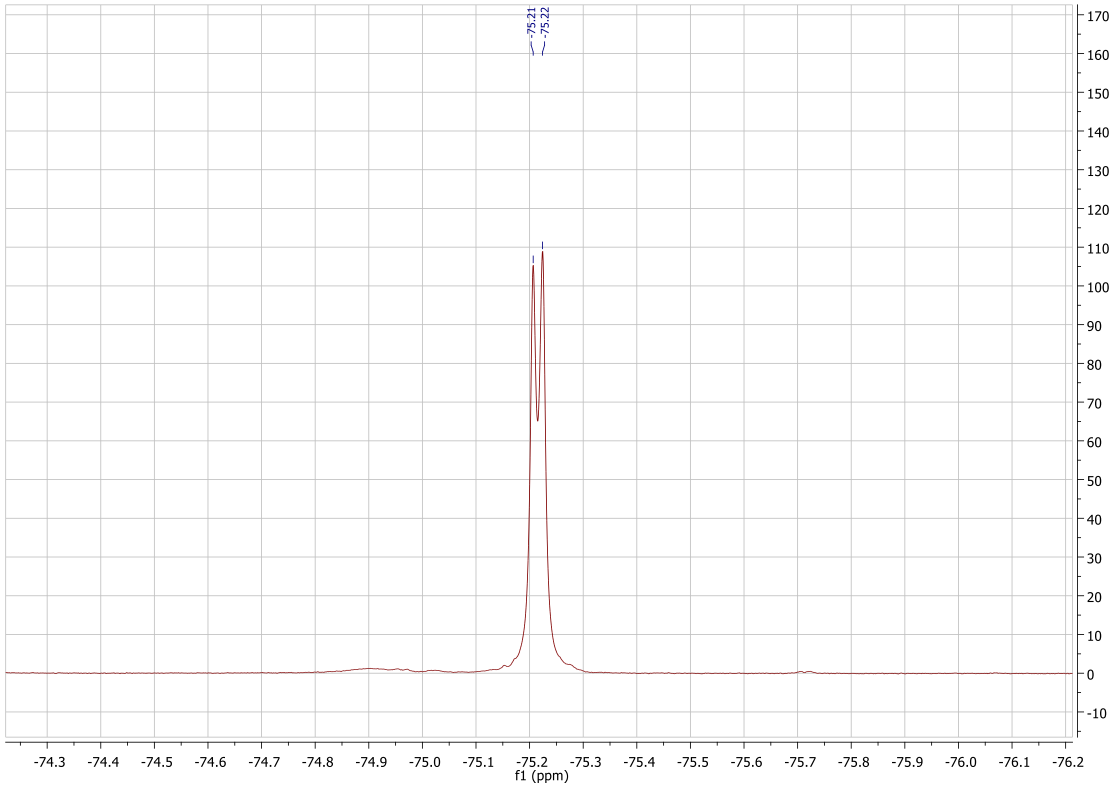

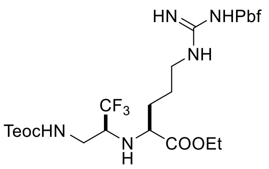

4g $^{13}C\{^{1}H\}$ NMR (101 MHz, $CDCl_3$)

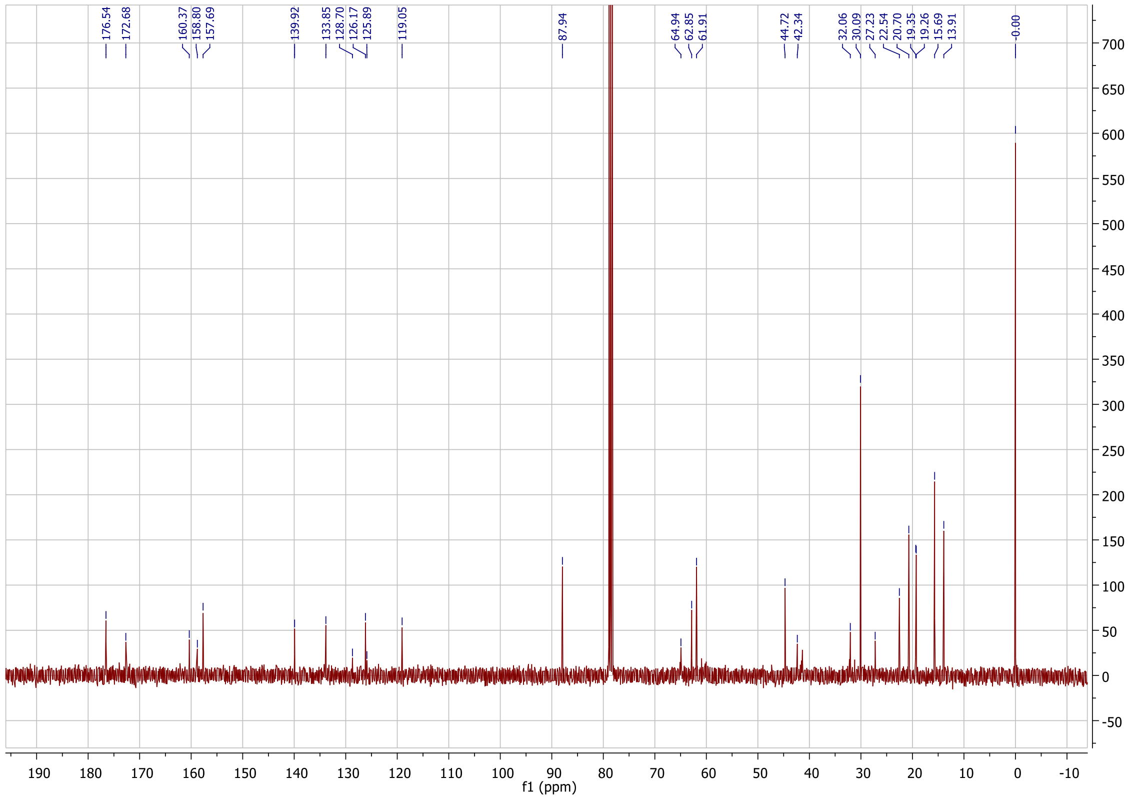

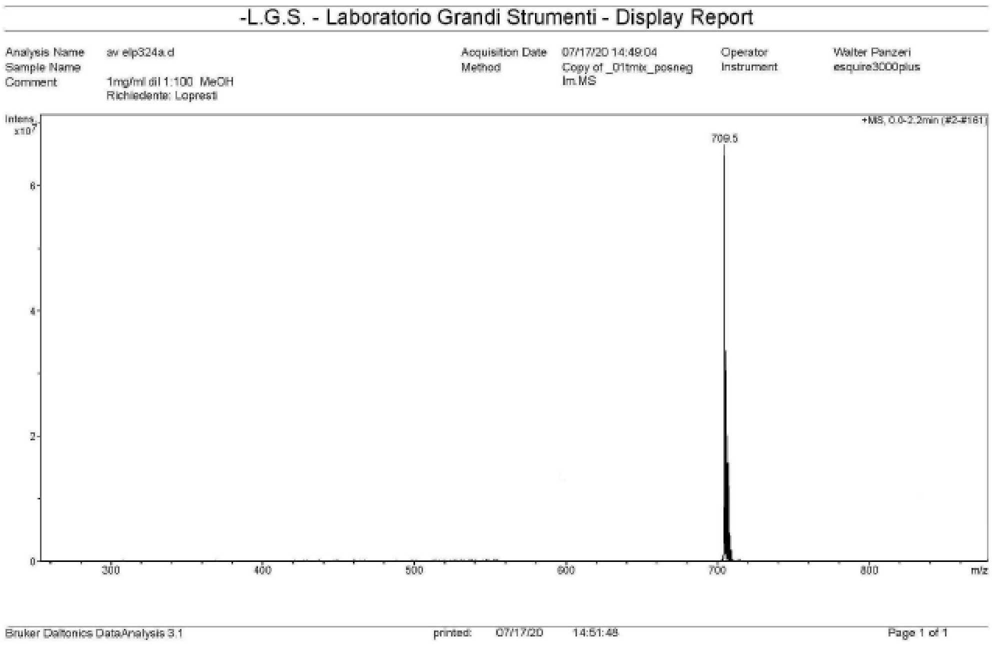

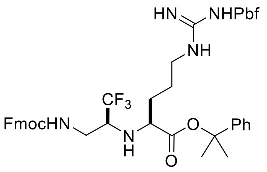

4h ^{1}H NMR (400 MHz, CD_3OD); ^{19}F NMR (376 MHz, CD_3OD)

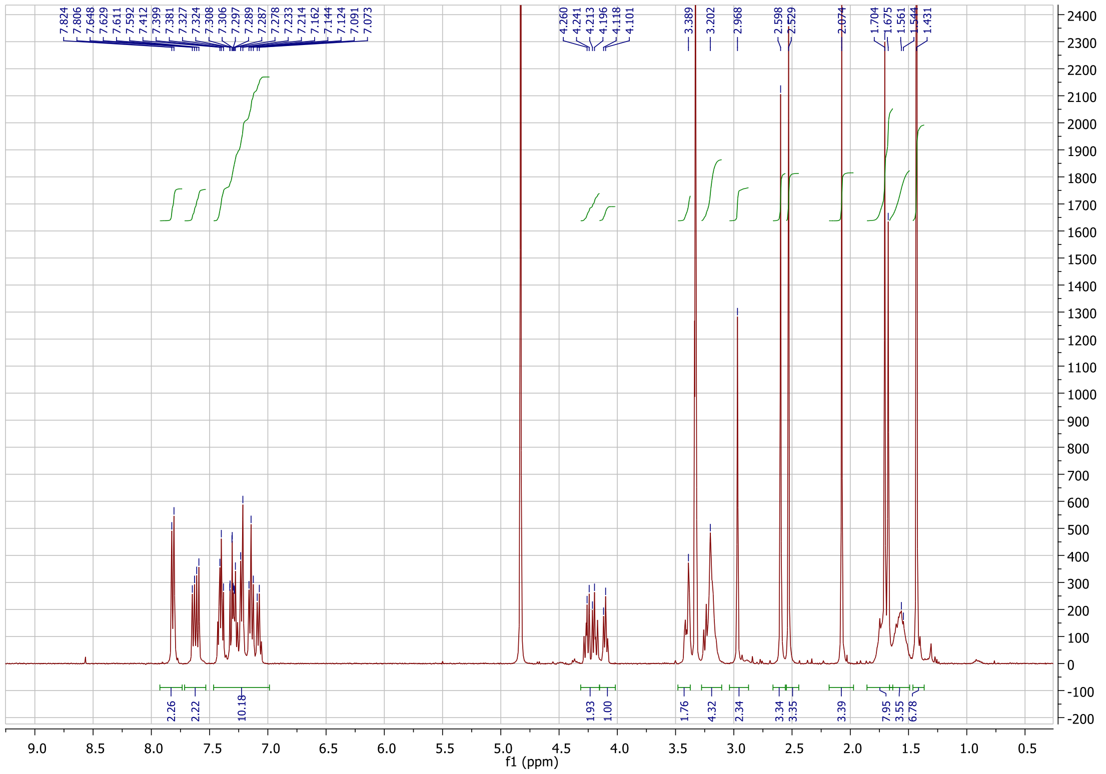

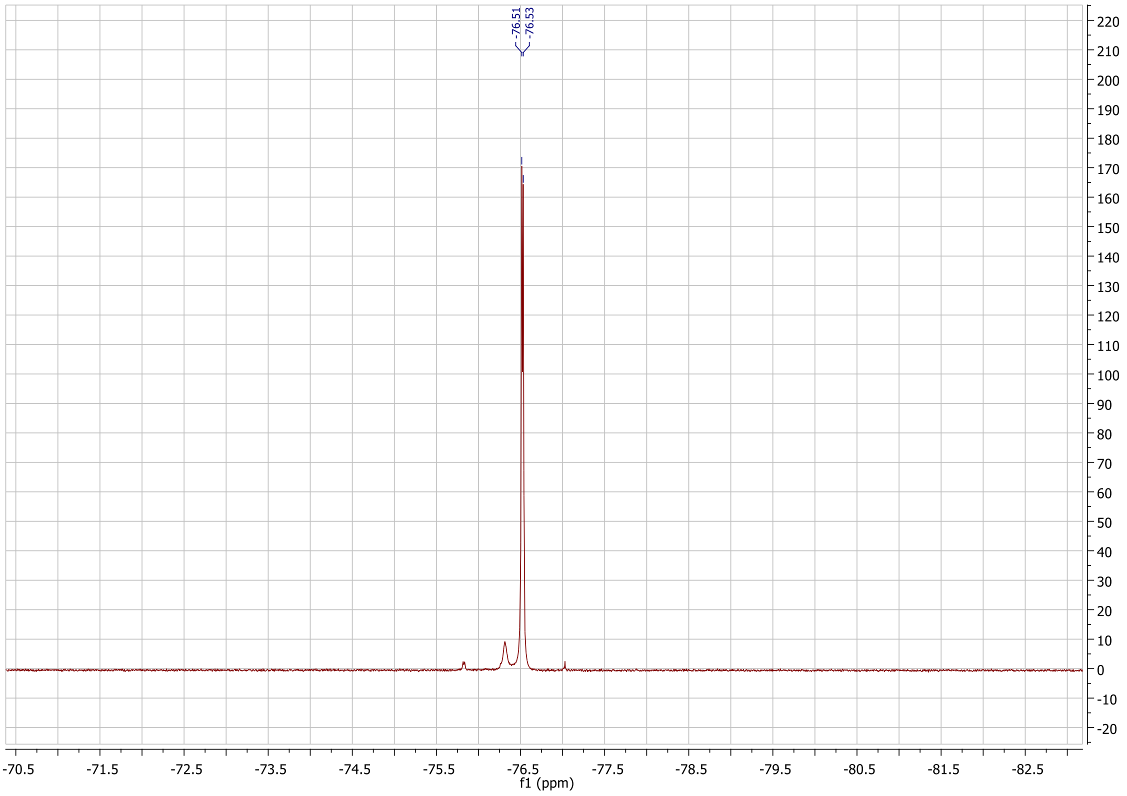

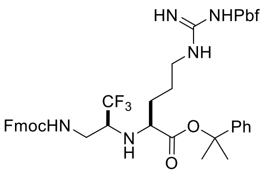

4h $^{13}C\{^{1}H\}$ NMR (101 MHz, $CDCl_3$)

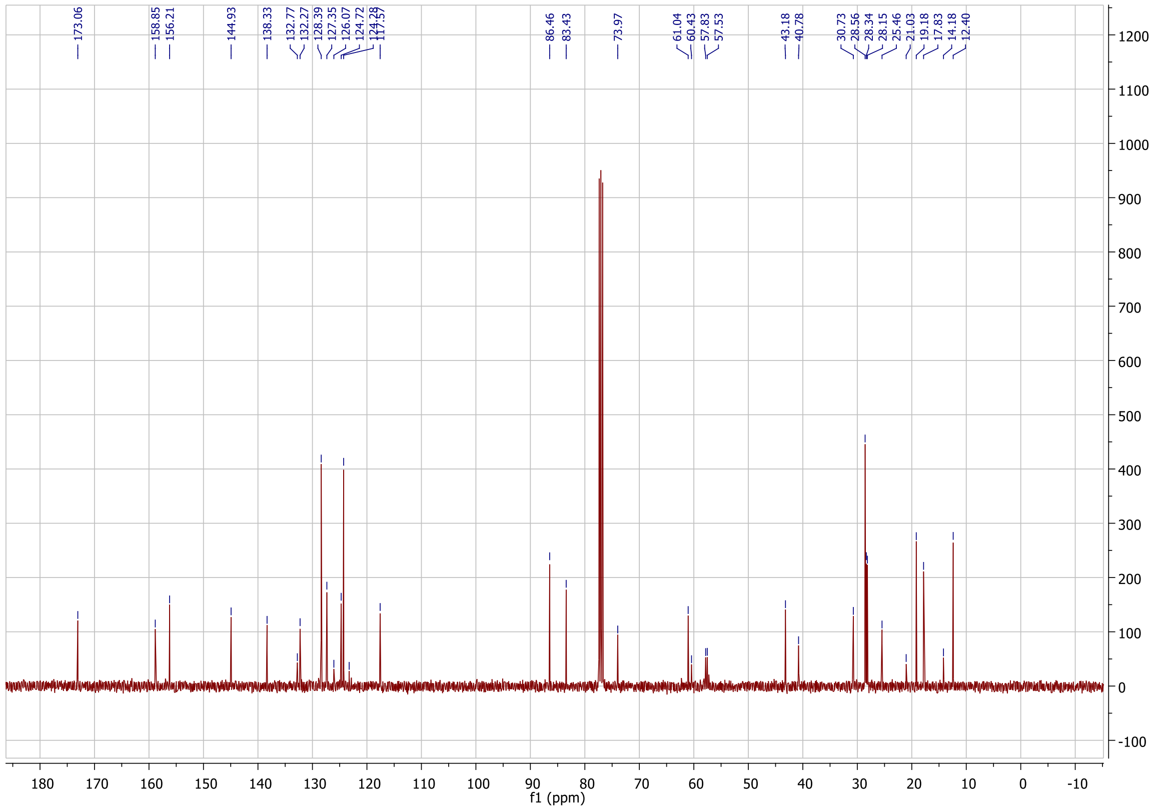

-L.G.S. - Laboratorio Grandi Strumenti - Display Report

Analysis Name	av cs81_2.d	Acquisition Date	10/16/19 11:47:17	Operator	Walter Panzeri
Sample Name		Method	Copy of _01tmix_posneg lm.MS	Instrument	esquire3000plus
Comment	1mg/ml dil 1:100 CH3CN Richiedente: Sgorbati				

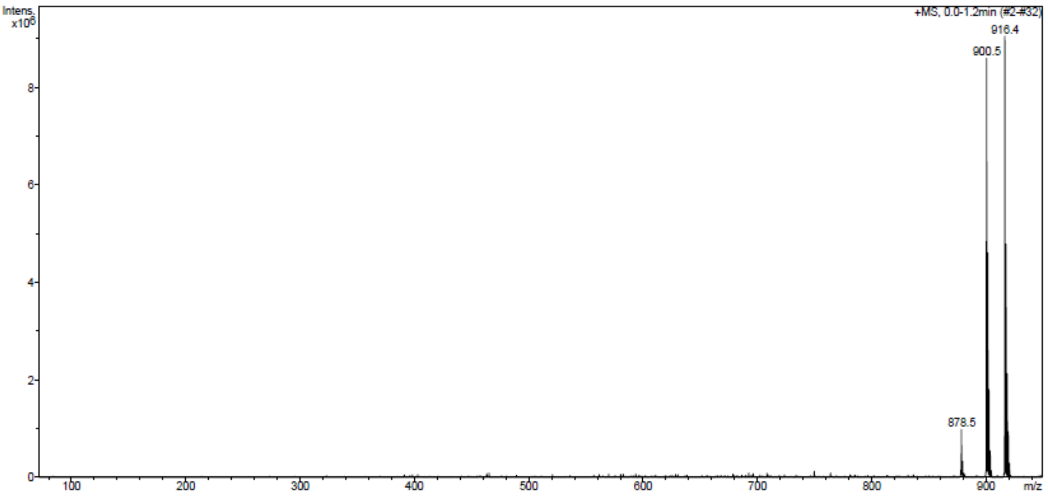

Bruker Daltonics DataAnalysis 3.1 printed: 10/16/19 11:49:22 Page 1 of 1

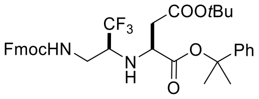

4i ^{1}H NMR (400 MHz, CD_3OD); ^{19}F NMR (376 MHz, CD_3OD)

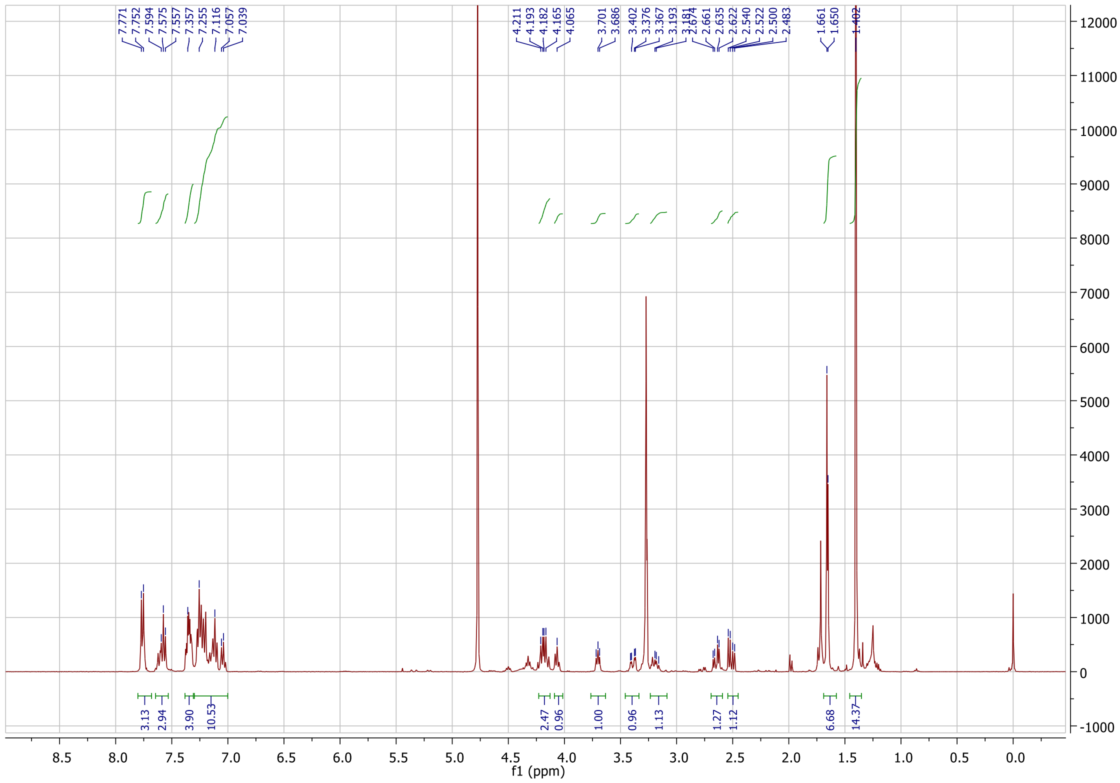

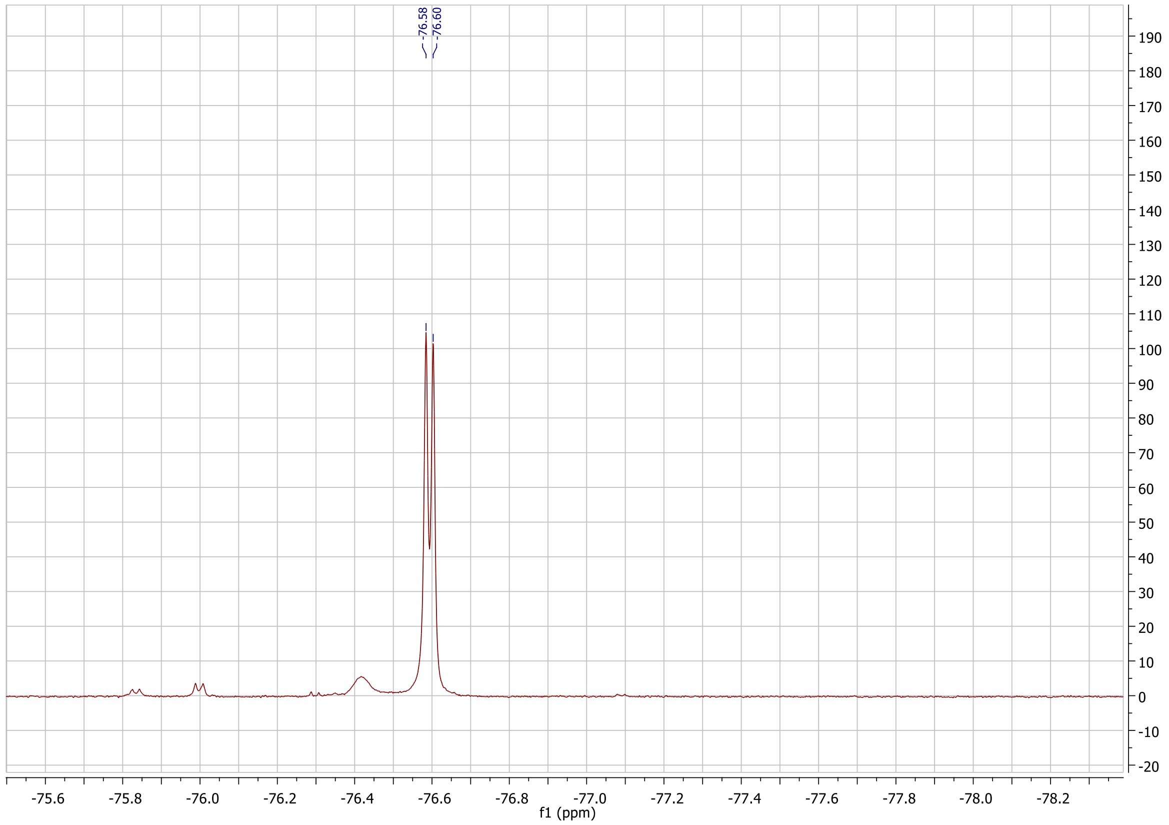

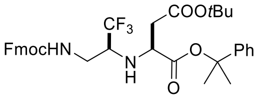

4i $^{13}C\{^{1}H\}$ NMR (101 MHz, CD_3OD)

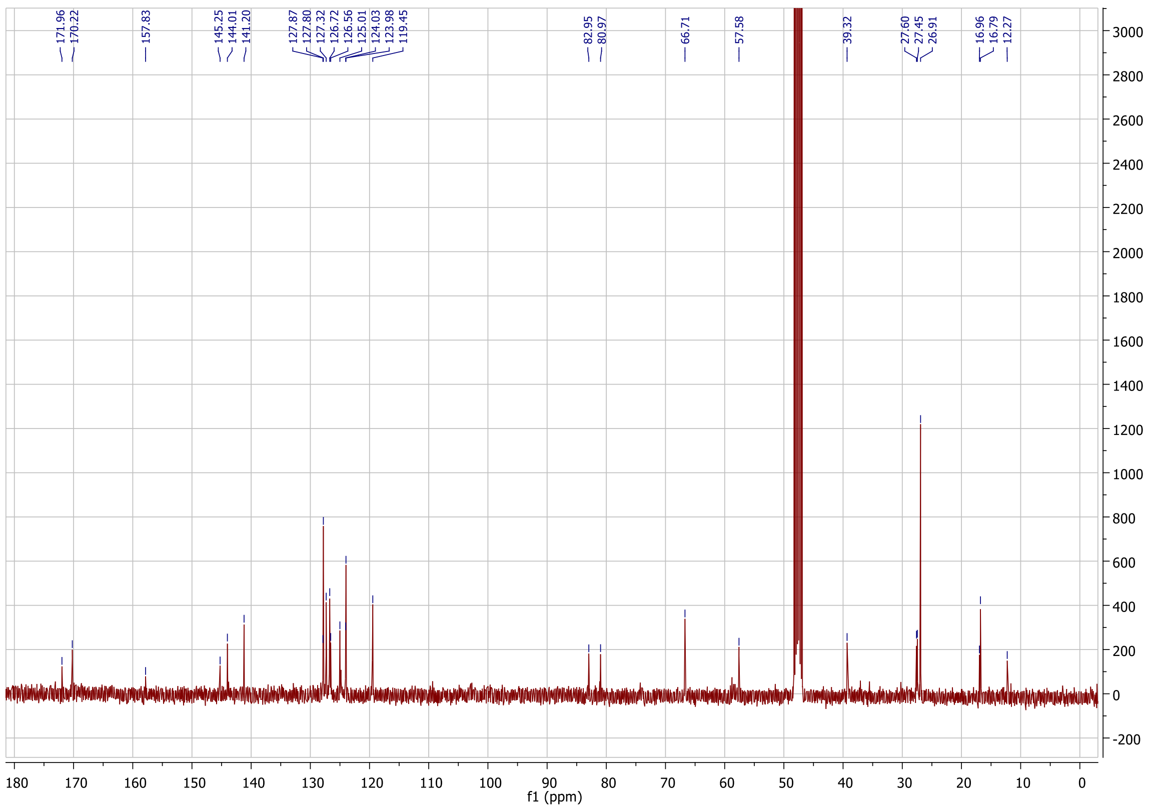

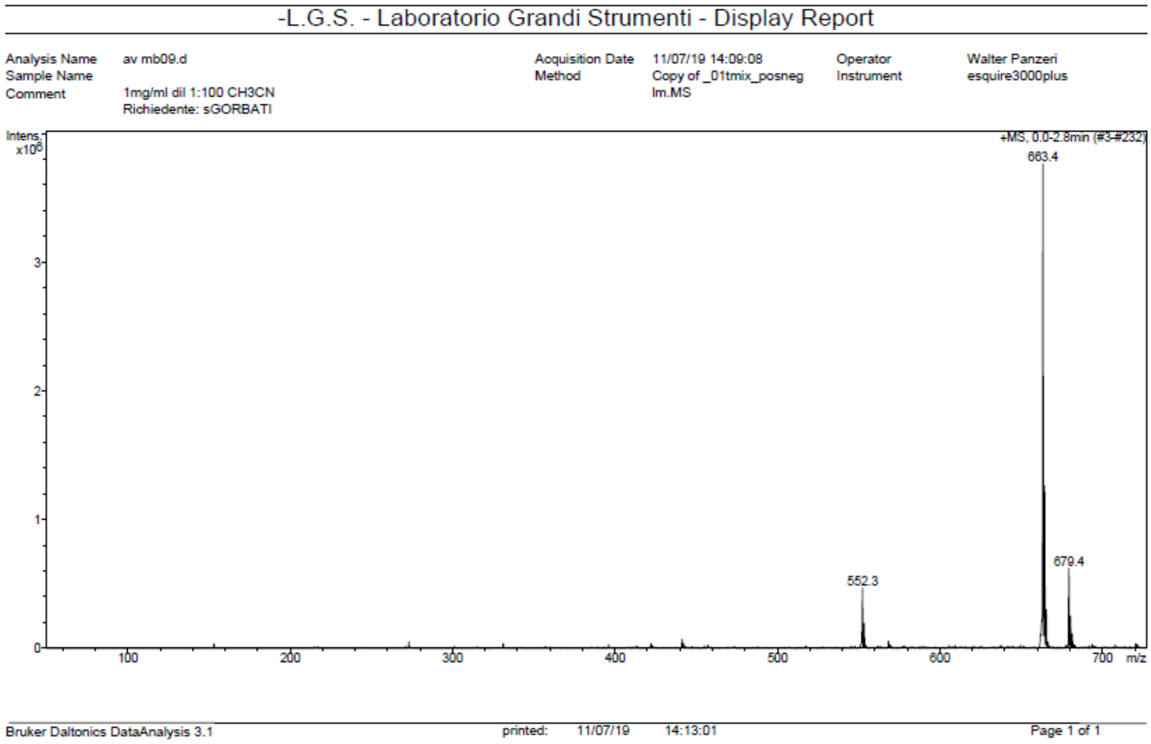

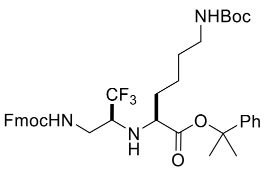

4j ^{1}H NMR (400 MHz, CD_3OD); ^{19}F NMR (376 MHz, CD_3OD)

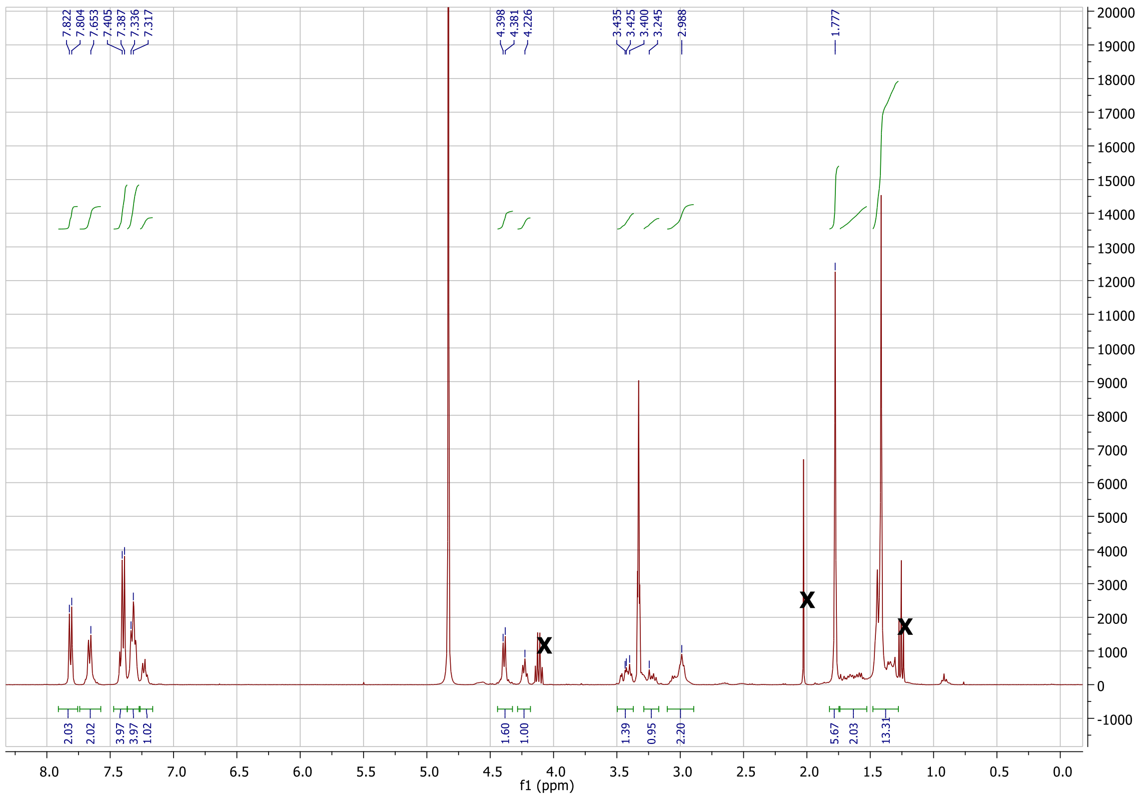

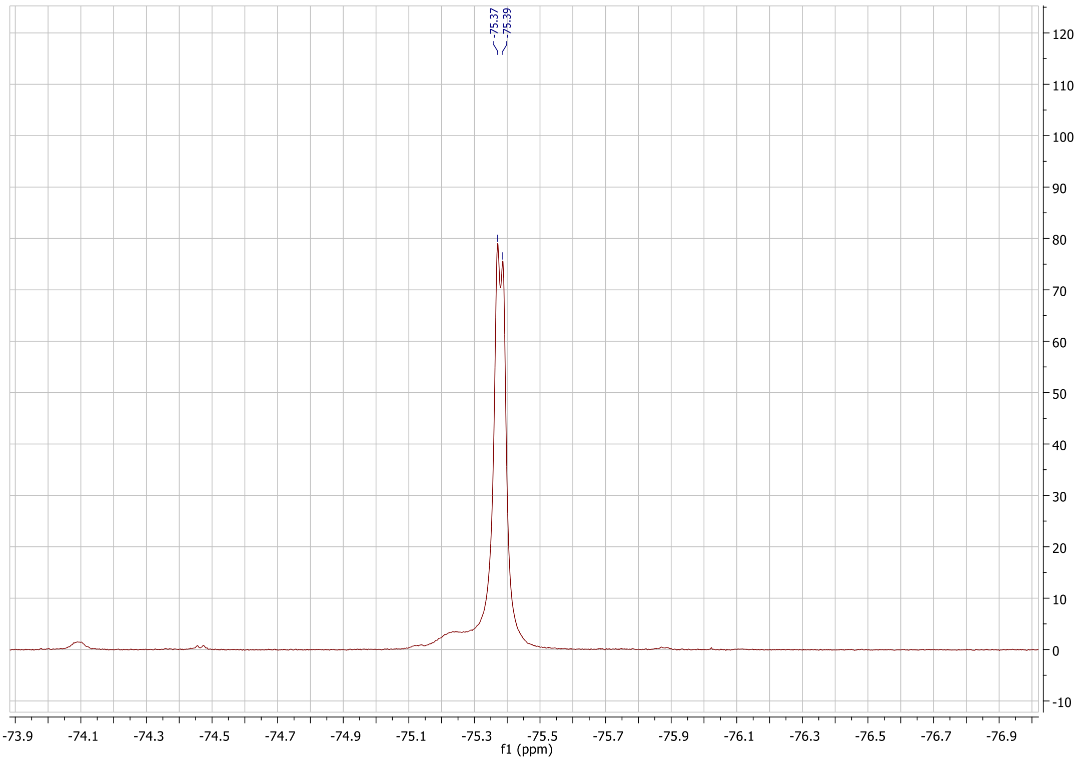

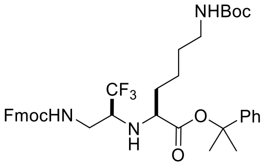

4j $^{13}C\{^{1}H\}$ NMR (101 MHz, $CDCl_3$)

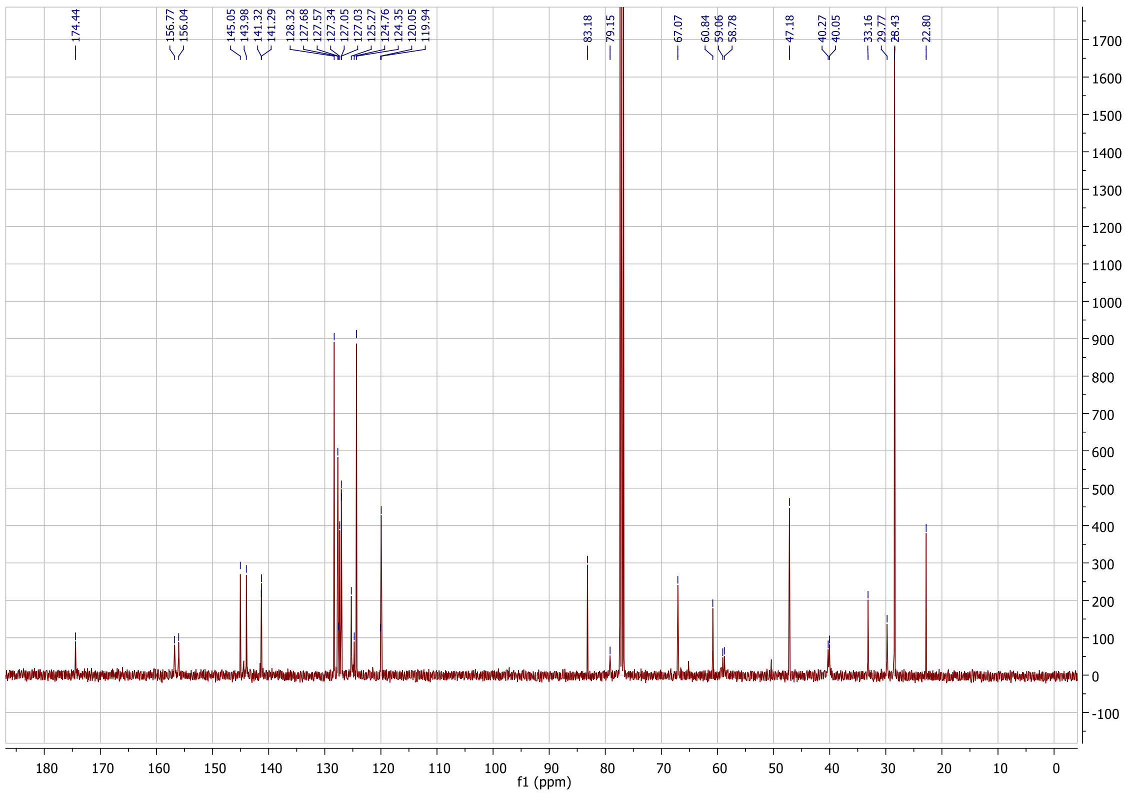

-L.G.S. - Laboratorio Grandi Strumenti - Display Report

Analysis Name	av mb04.d	Acquisition Date	10/24/19 13:58:37	Operator	Walter Panzeri
Sample Name		Method	Copy of _01tmix_posneg lm.MS	Instrument	esquire3000plus
Comment	1 mg/ml dil 1:100 CH3CN Richiedente: Sgorbati				

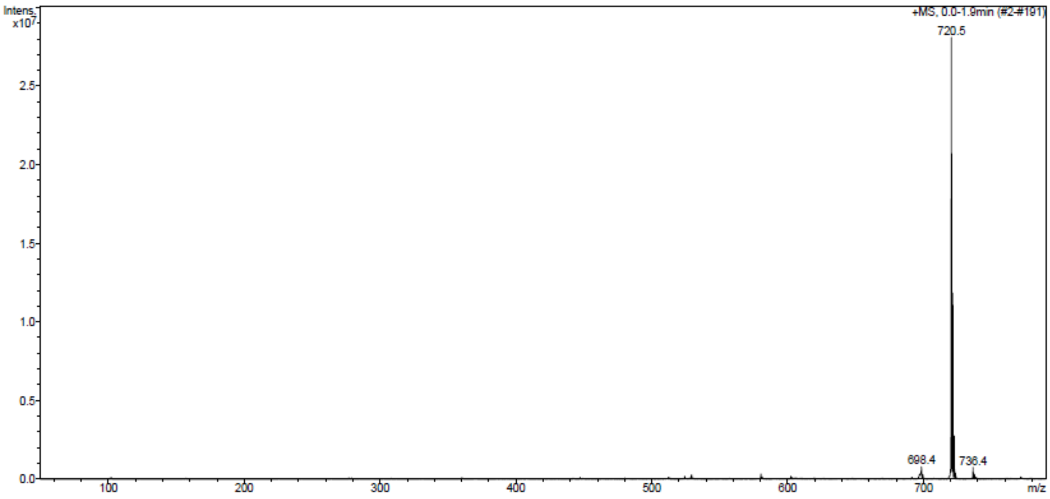

Bruker Daltonics DataAnalysis 3.1 printed: 10/24/19 14:03:15 Page 1 of 1

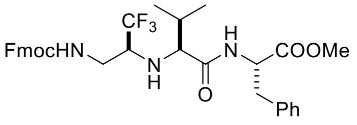

5a ^{1}H NMR (400 MHz, CD_3OD); ^{19}F NMR (376 MHz, CD_3OD)

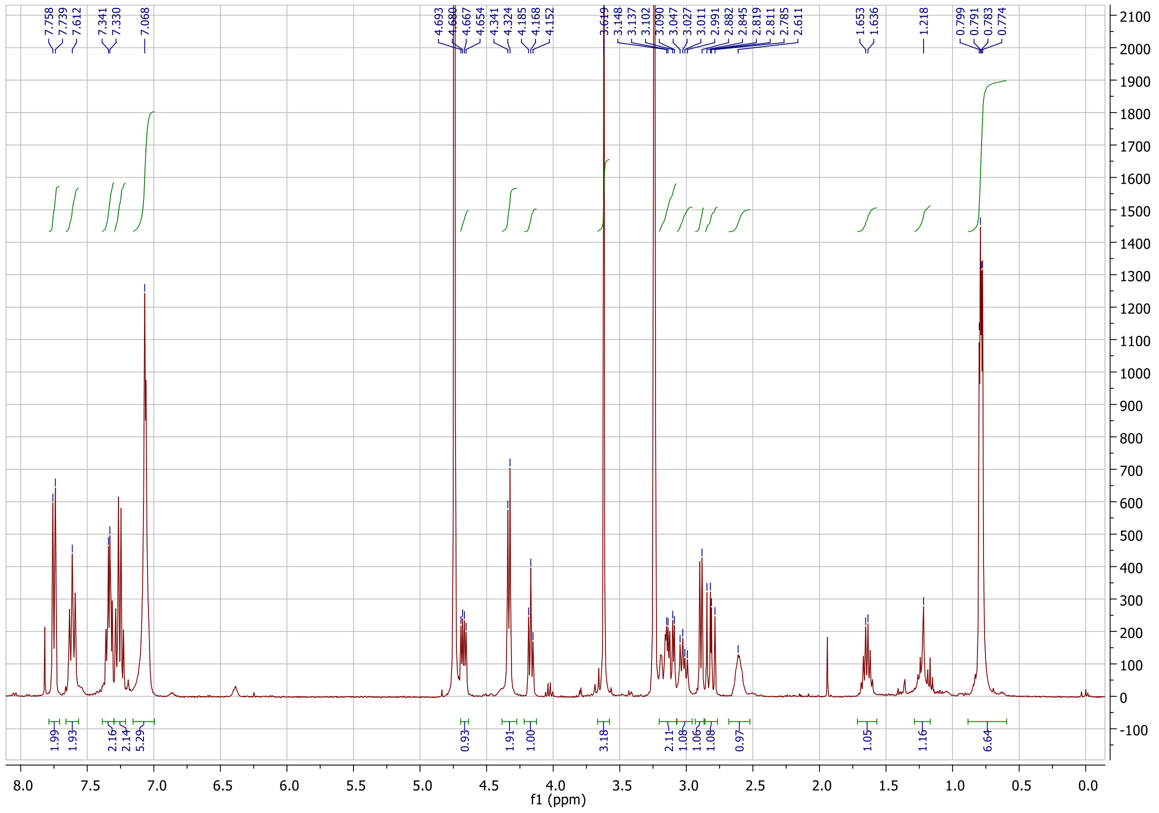

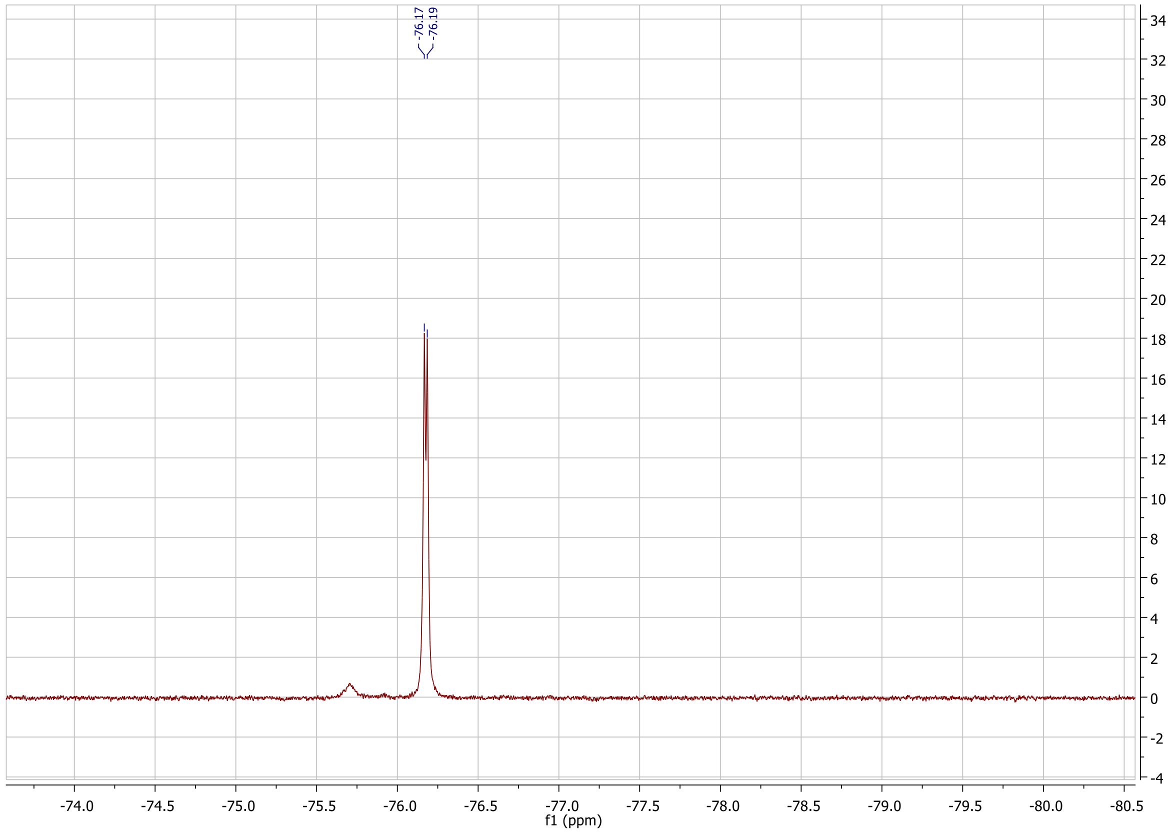

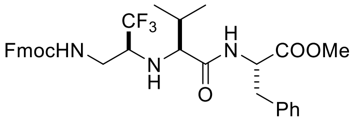

5a $^{13}C\{^{1}H\}$ NMR (101 MHz, $CDCl_3$)

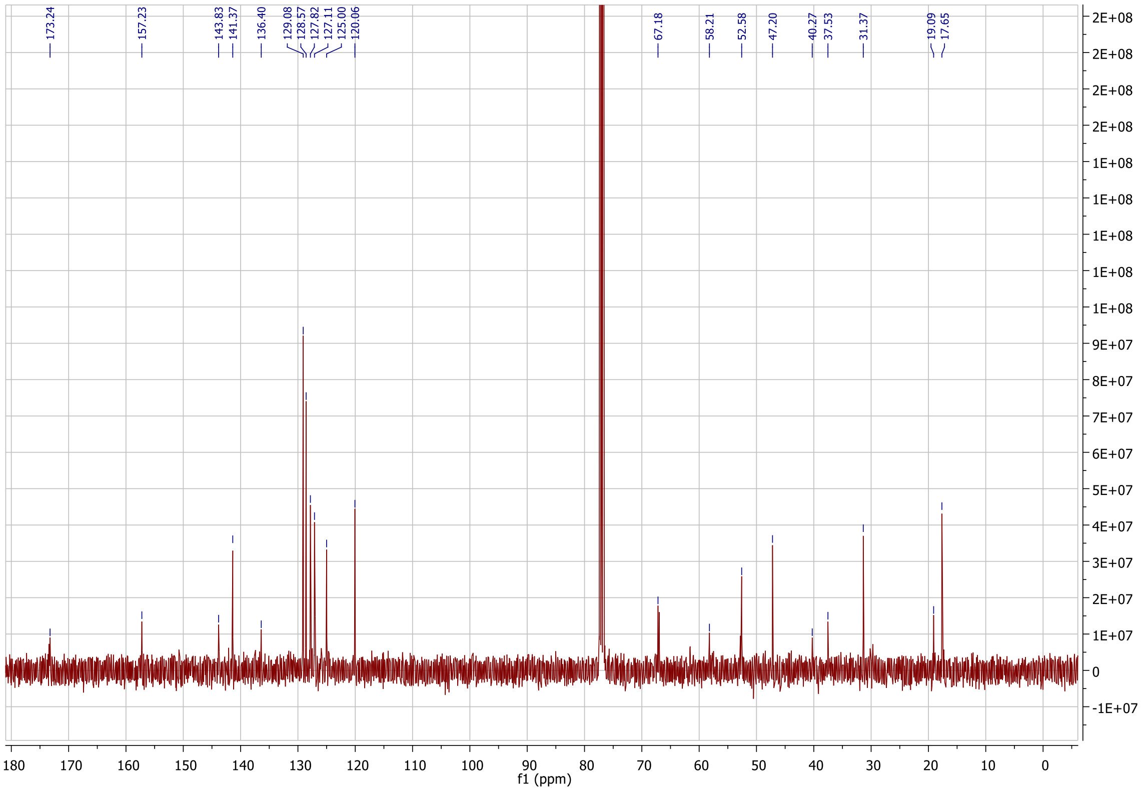

-L.G.S. - Laboratorio Grandi Strumenti - Display Report

Analysis Name	av cs_41.d	Acquisition Date	06/06/19 15:31:47	Operator	Walter Panzeri
Sample Name		Method	Copy of _01tmix_posneg lm.MS	Instrument	esquire3000plus
Comment	1mg/ml dil 1:100 MeOH Richiedente: Sgorbati				

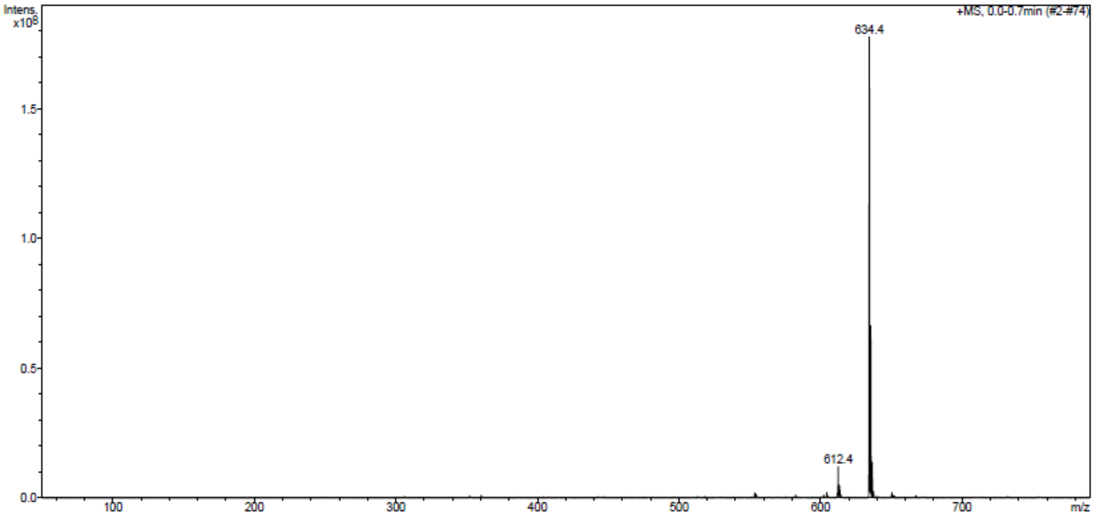

Bruker Daltonics DataAnalysis 3.1 printed: 06/06/19 15:35:56 Page 1 of 1

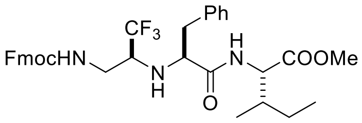

5b ^{1}H NMR (400 MHz, $CDCl_3$); ^{19}F NMR (376 MHz, $CDCl_3$)

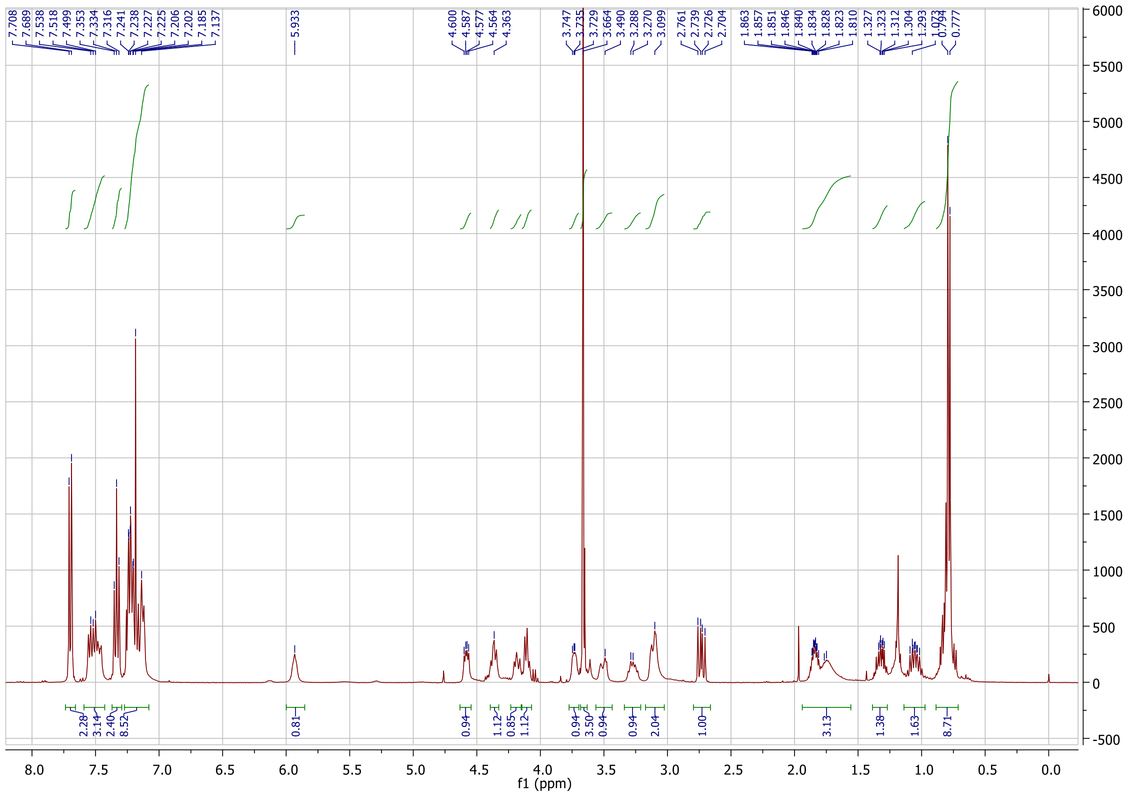

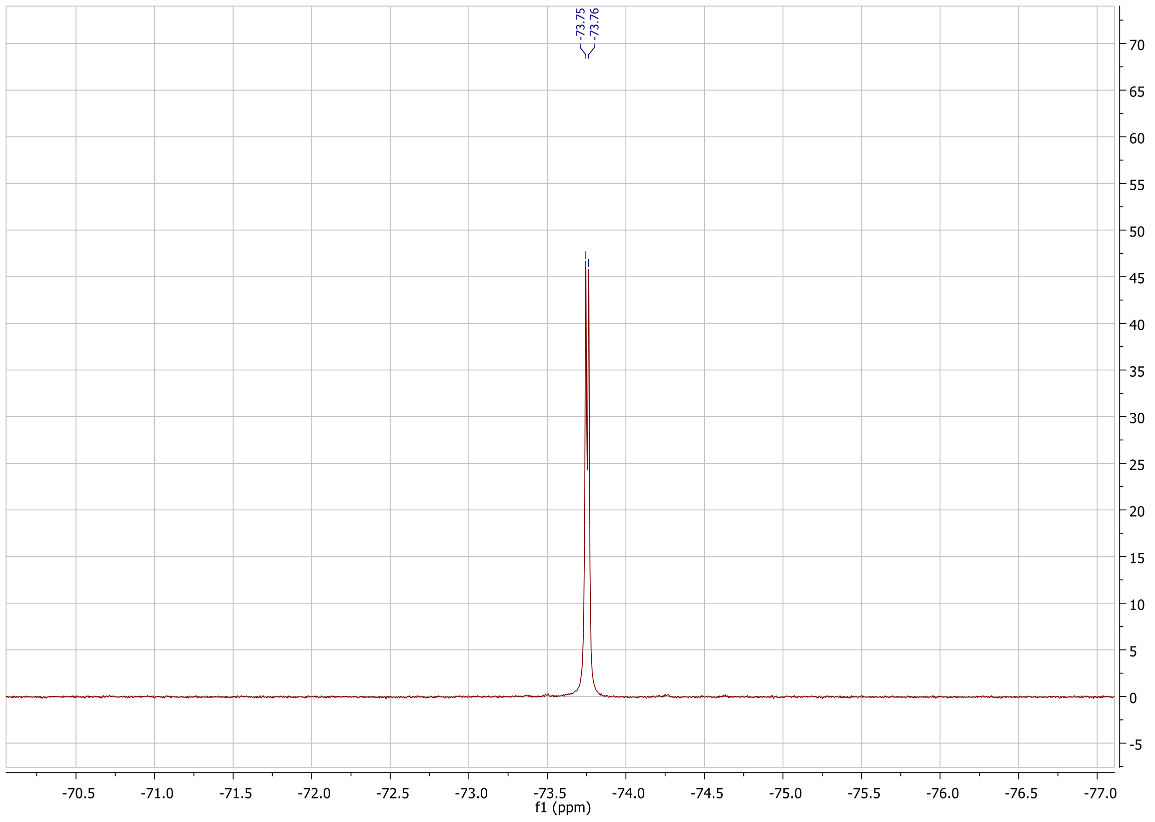

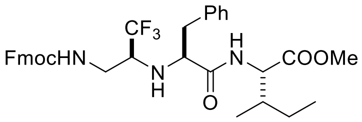

5b $^{13}C\{^{1}H\}$ NMR (101 MHz, $CDCl_3$)

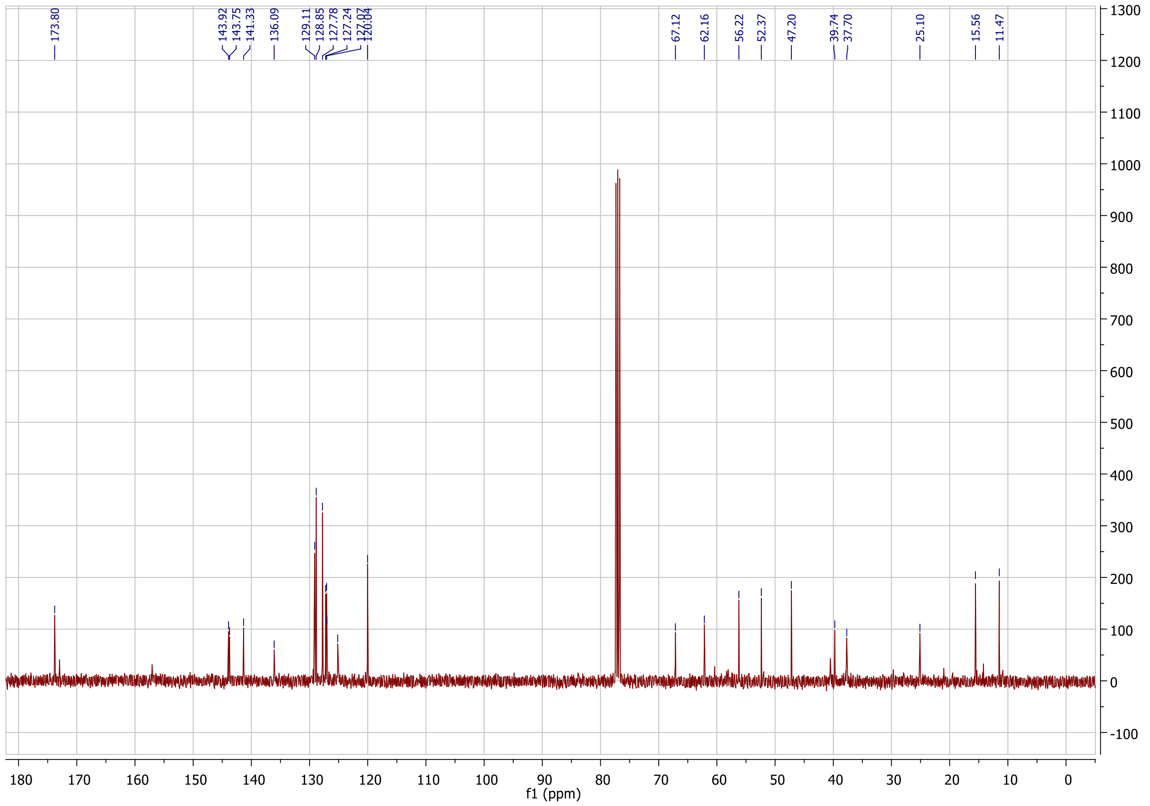

-L.G.S. - Laboratorio Grandi Strumenti - Display Report

Analysis Name	av cs51.d	Acquisition Date	11/07/19 11:43:09	Operator	Walter Panzeri
Sample Name		Method	Copy of _01tmix_posneg lm.MS	Instrument	esquire3000plus
Comment	1mg/ml DMSO dil 1:100 CH3CN<br>Richiedente: Sgorbati				

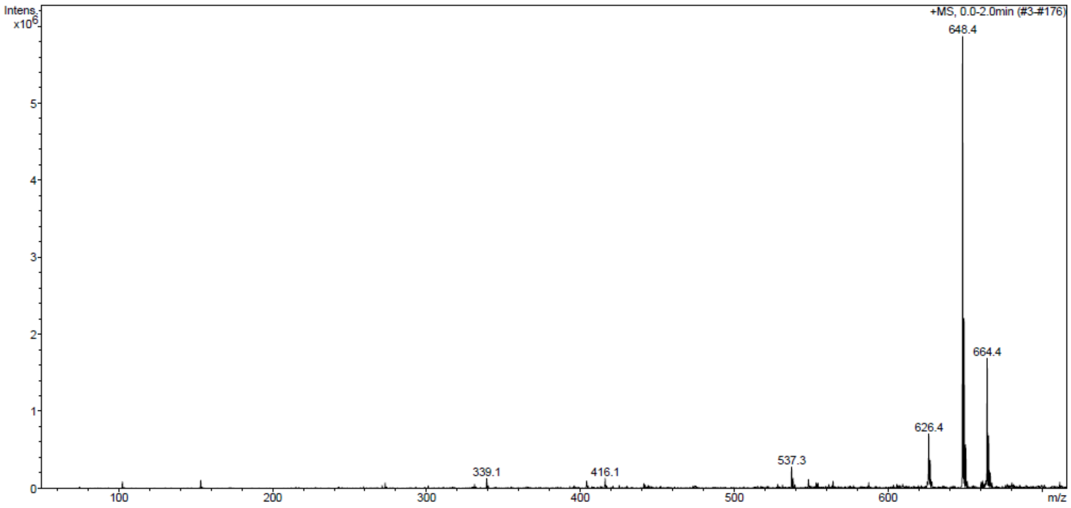

Bruker Daltonics DataAnalysis 3.1 printed: 11/07/19 11:46:38 Page 1 of 1

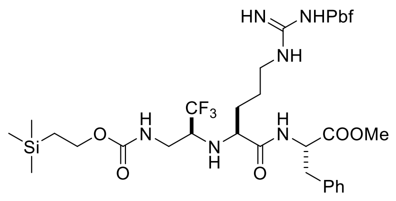

5c ^{1}H NMR (400 MHz, CD_3OD); ^{19}F NMR (376 MHz, CD_3OD)

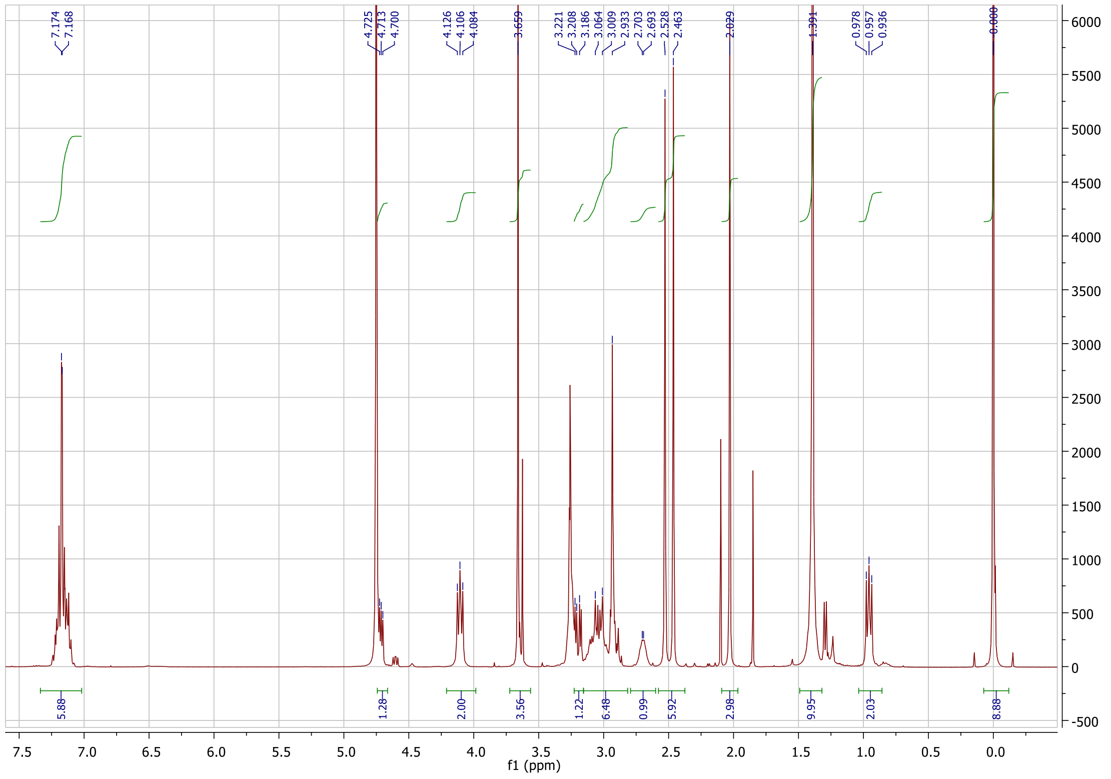

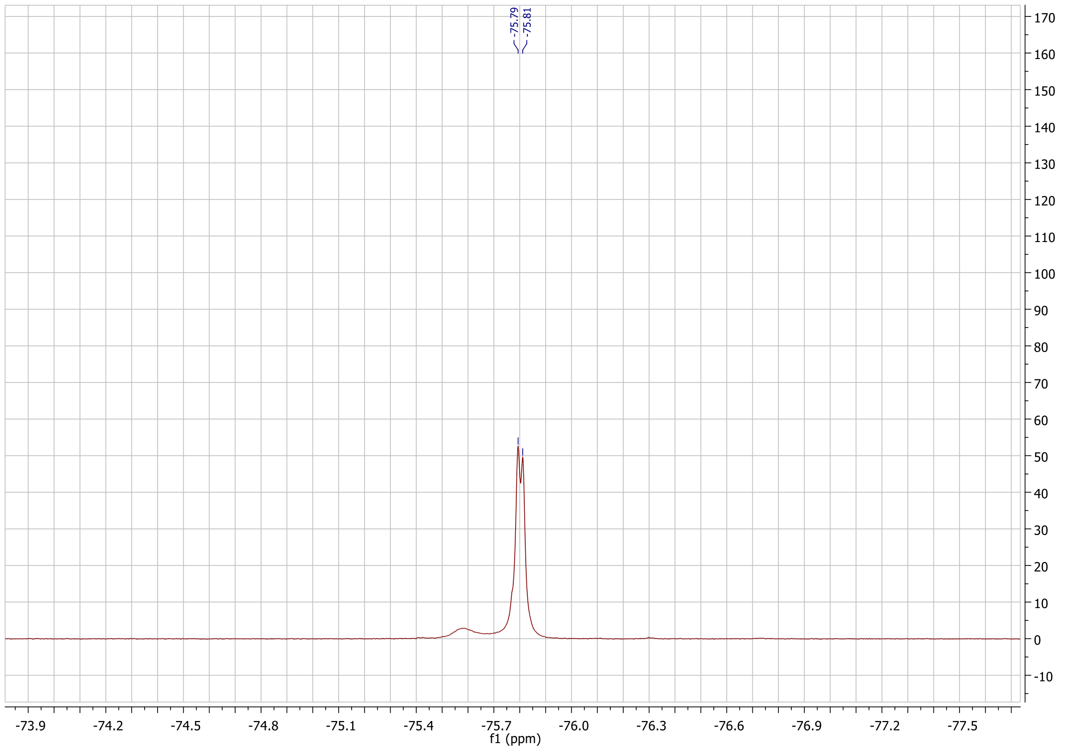

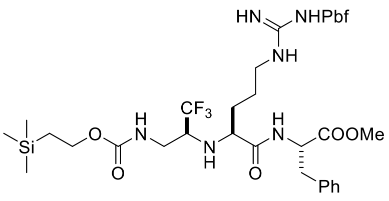

5c $^{13}C\{^{1}H\}$ NMR (101 MHz, CD_3OD)

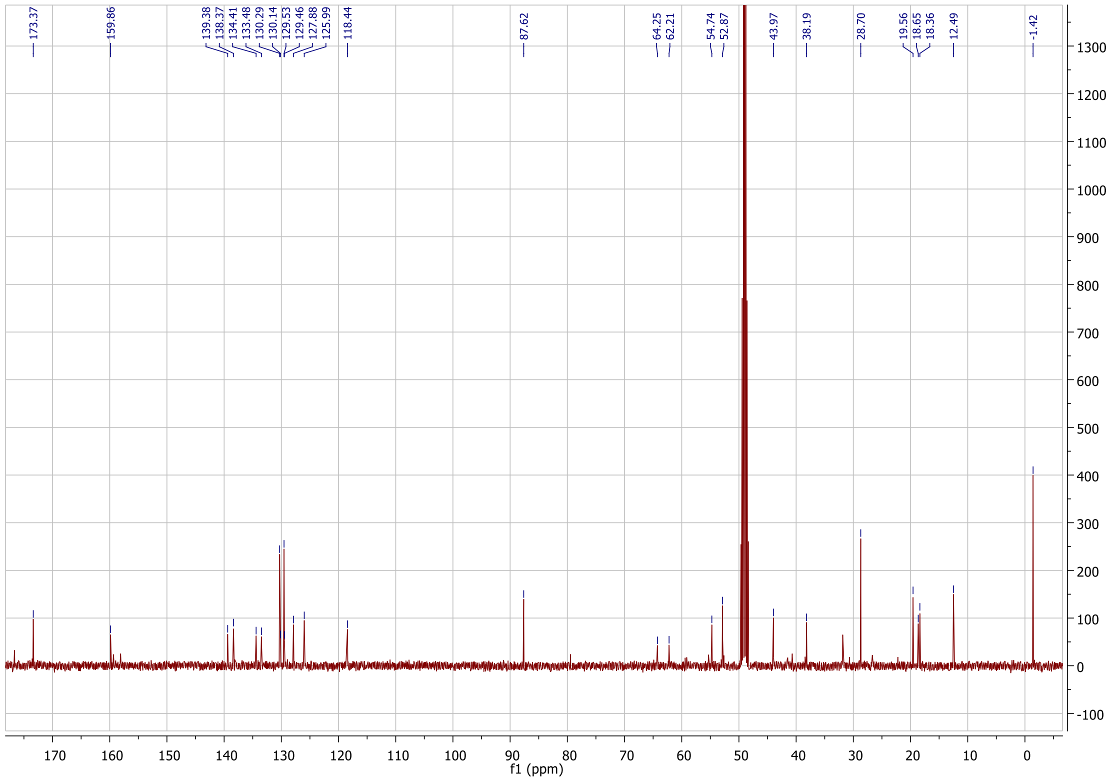

-L.G.S. - Laboratorio Grandi Strumenti - Display Report

Analysis Name av cs40b.d
Sample Name
Comment 1mg/ml dil 1:100 MeOH
Richiedente: Sgorbati

Acquisition Date 06/03/19 11:51:48
Method Copy of _01tmix_posneg
lm.MS

Operator Walter Panzeri
Instrument esquire3000plus

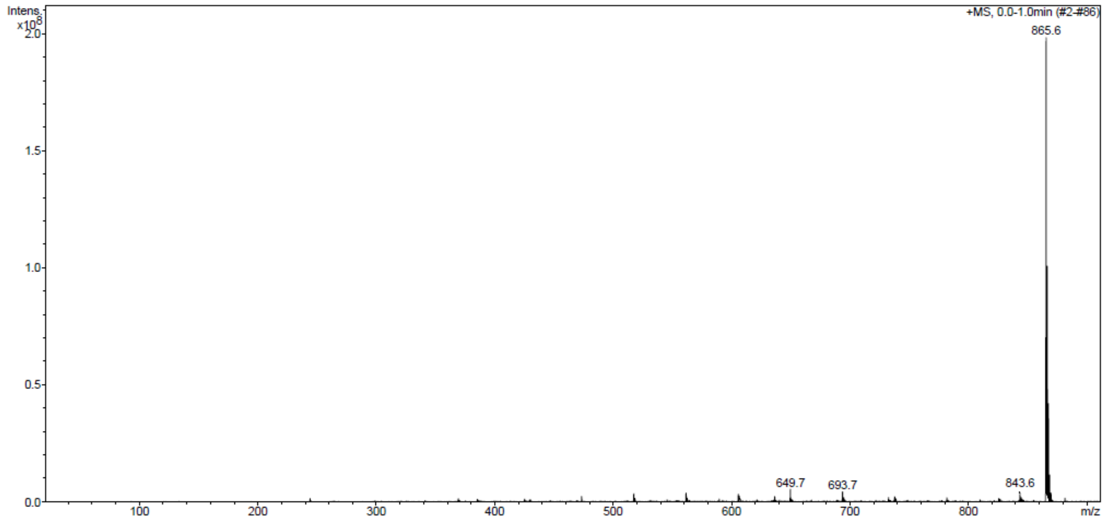

Bruker Daltonics DataAnalysis 3.1 printed: 06/03/19 11:53:52 Page 1 of 1

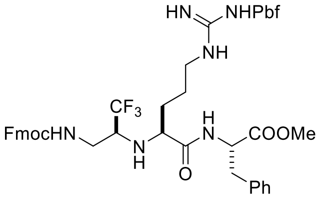

5d ^{1}H NMR (400 MHz, $CDCl_3$); ^{19}F NMR (376 MHz, $CDCl_3$)

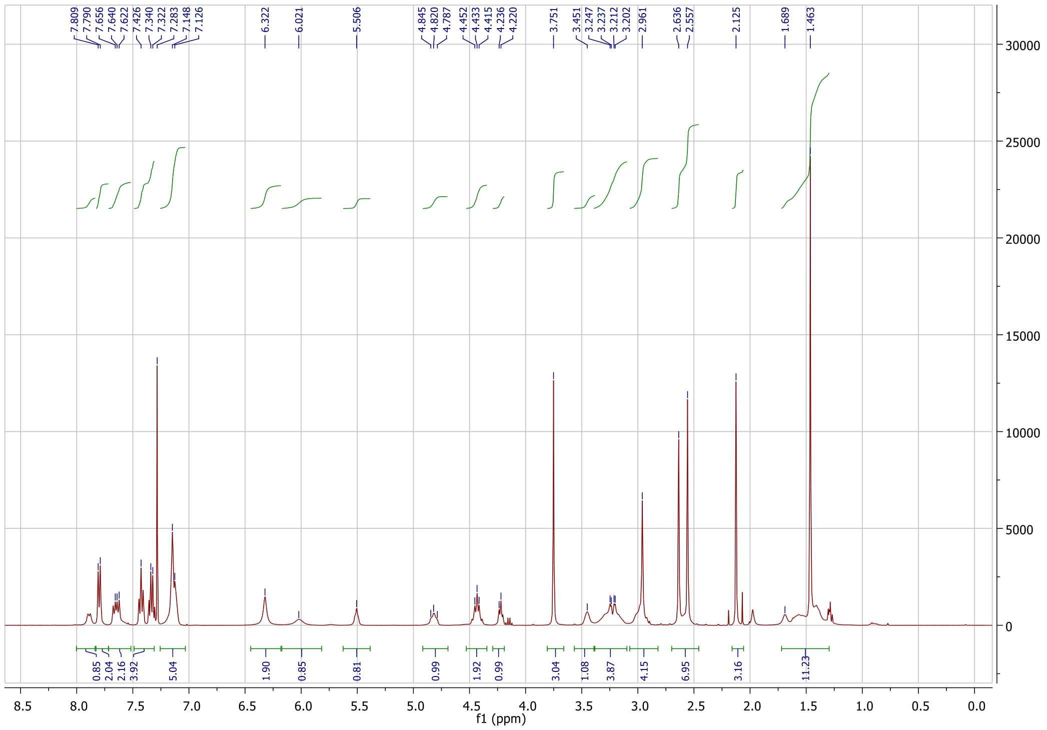

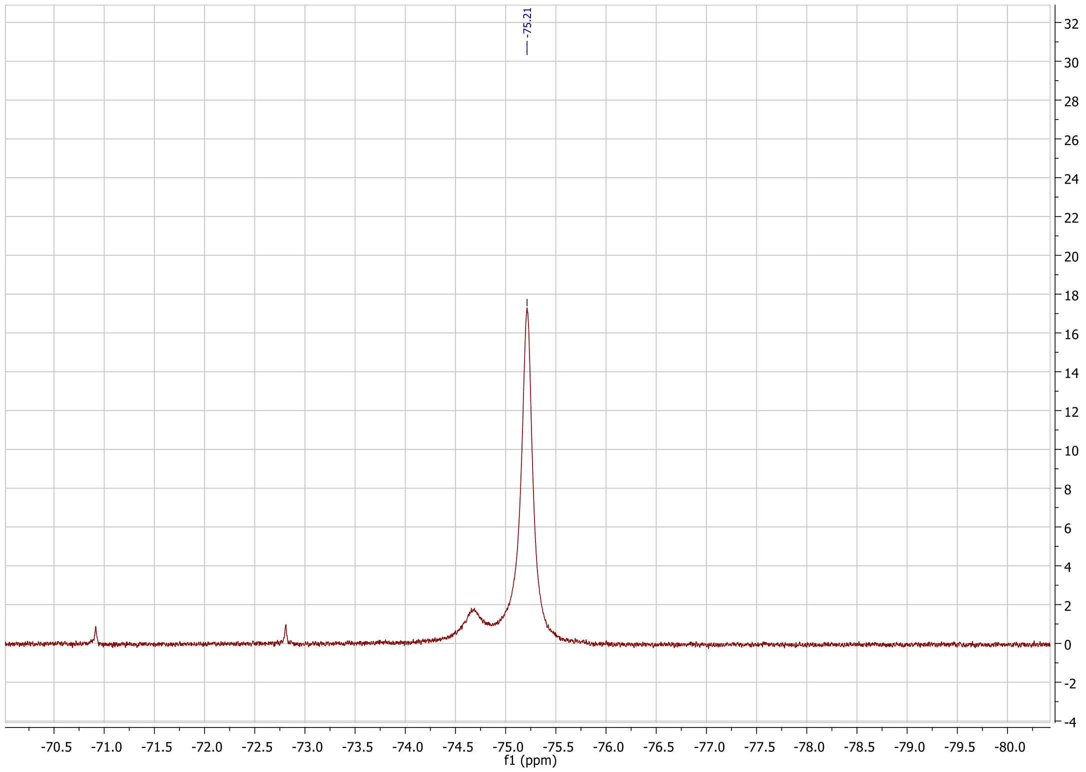

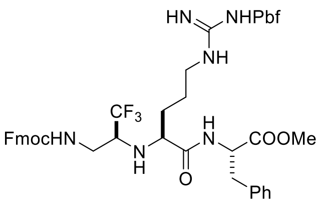

5d $^{13}C\{^{1}H\}$ NMR (101 MHz, $CDCl_3$)

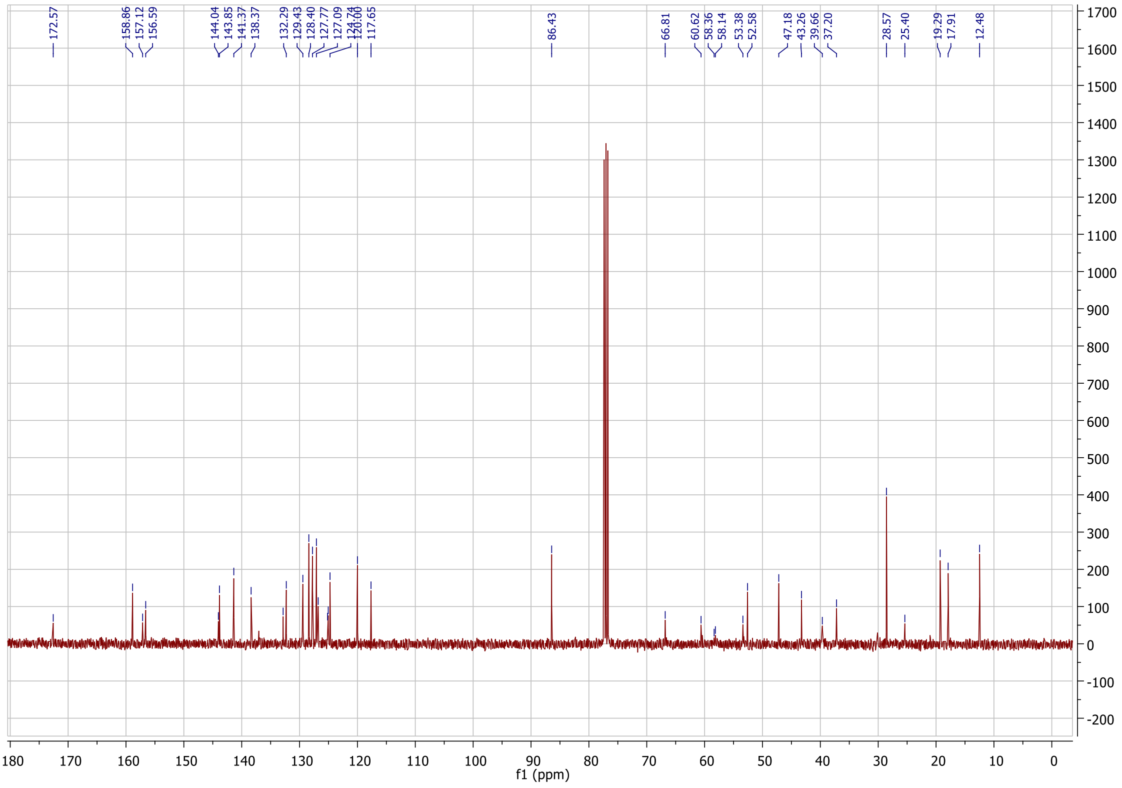

-L.G.S. - Laboratorio Grandi Strumenti - Display Report

Analysis Name	av elp422.d	Acquisition Date	03/09/21 08:26:31	Operator	Walter Panzeri
Sample Name		Method	Copy of $PW_TBLM.MS	Instrument	esquire3000plus
Comment	1 mg/ml dil 1:100 MeOH<br>Richiedente: Lopresti				

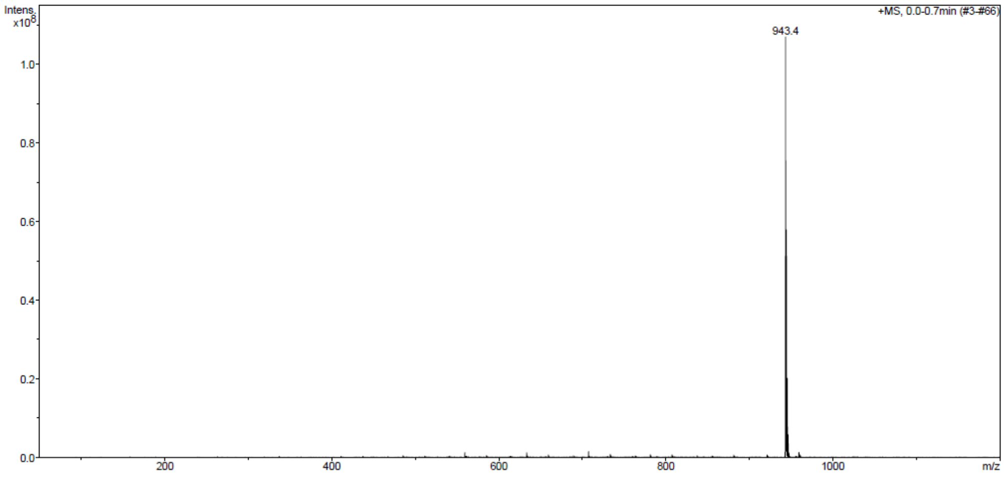

Bruker Daltonics DataAnalysis 3.1 printed: 03/09/21 08:36:15 Page 1 of 1

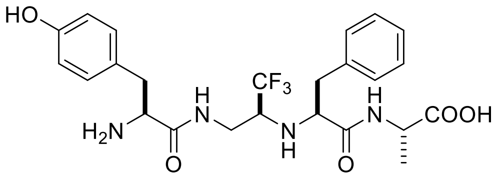

6

HPLC (Shimadzu C18, gradient A:B from 90/10 to 0/100, flow rate 1mL/min, l = 230 nm and 280 nm) tR: 11.124 min (major), 12.486 (minor).

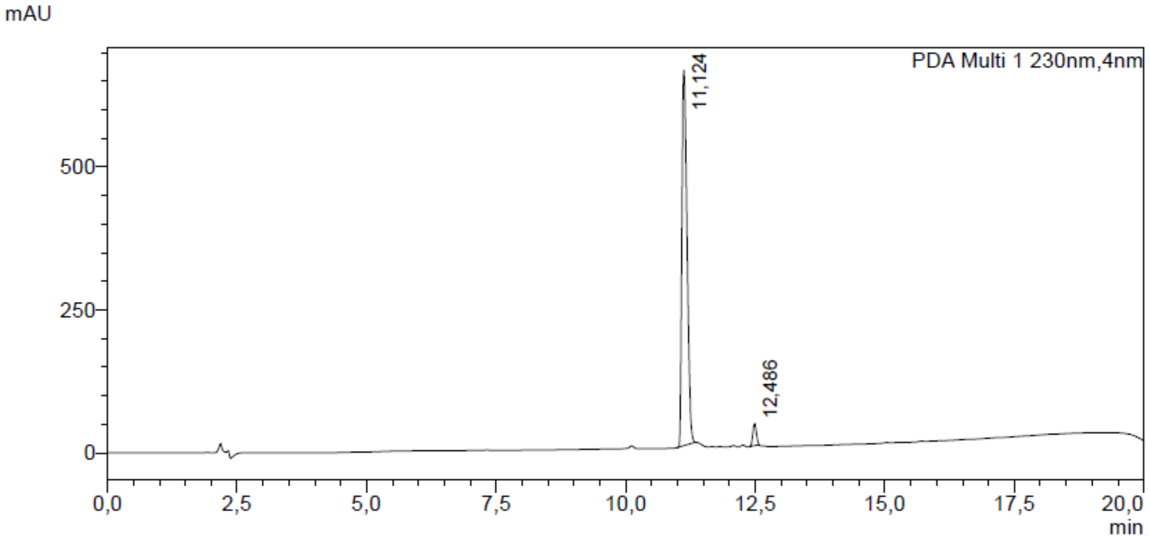

<Peak Table>

PDA Ch1 230nm

Peak#	Ret. Time	Area	Height	Conc.	Unit	Mark	Name
1	11,124	4601995	656871	96,088			
2	12,486	187380	38595	3,912			
Total		4789376	695466				

Analysis Name	av cs96_phe.d	Acquisition Date	11/20/19 11:35:26	Operator	Walter Panzeri
Sample Name		Method	Copy of _01tmix_posneg lm.MS	Instrument	esquire3000plus
Comment	1mg/ml dil 1:100 H2O Richiedente: Sgorbati				

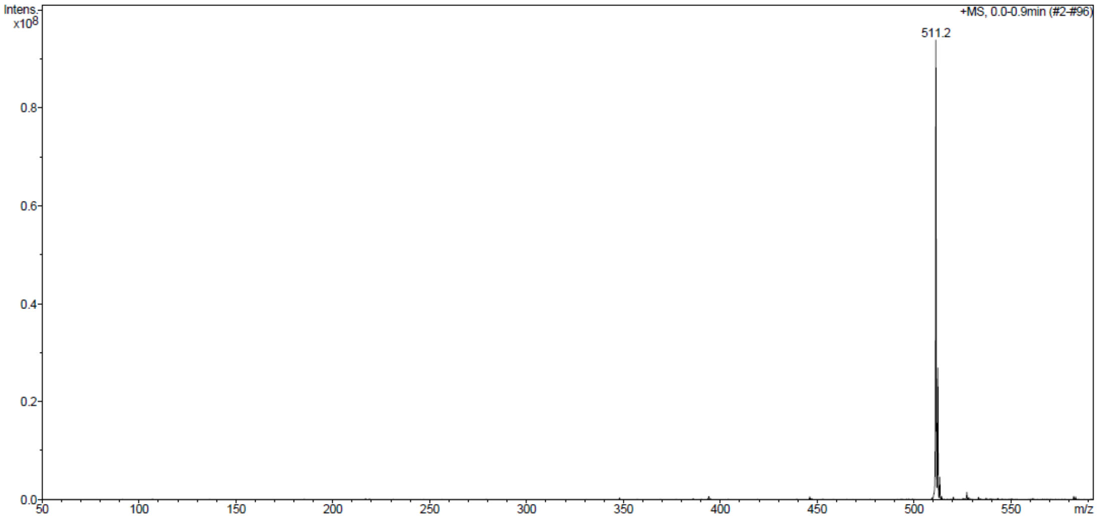

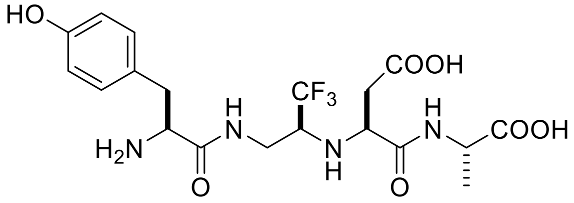

7

HPLC (Shimadzu C18, gradient A:B from 100/0 to 10/90, flow rate 1mL/min, l = 230 nm and 280 nm) tR: 8.662.

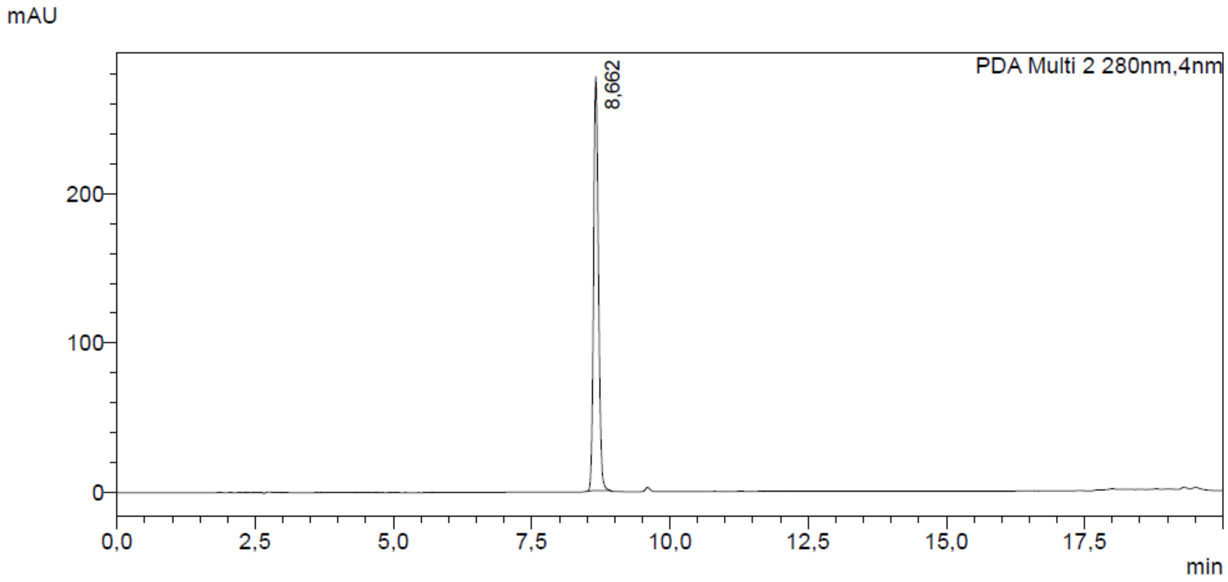

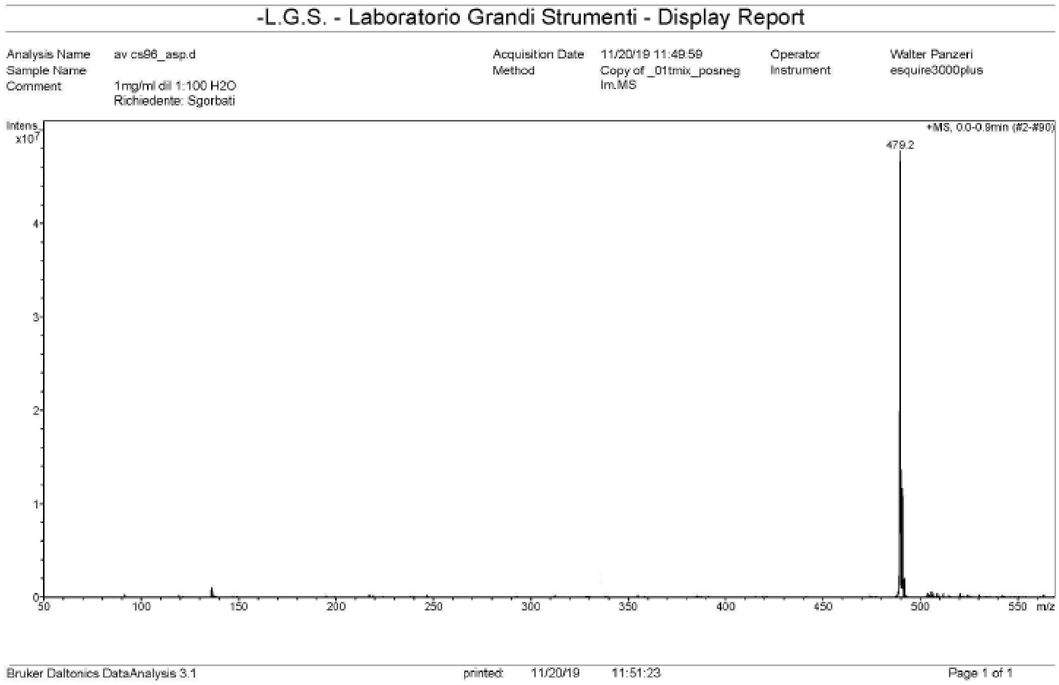

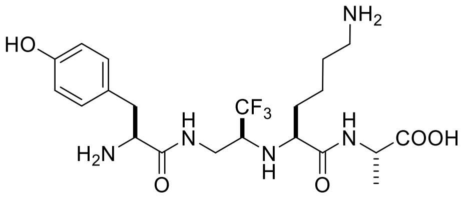

8

HPLC (Shimadzu C18, gradient A:B from 100/0 to 10/90, flow rate 1mL/min, l = 230 nm and 280 nm) tR: 5.594 (minor), 6.904 (major).

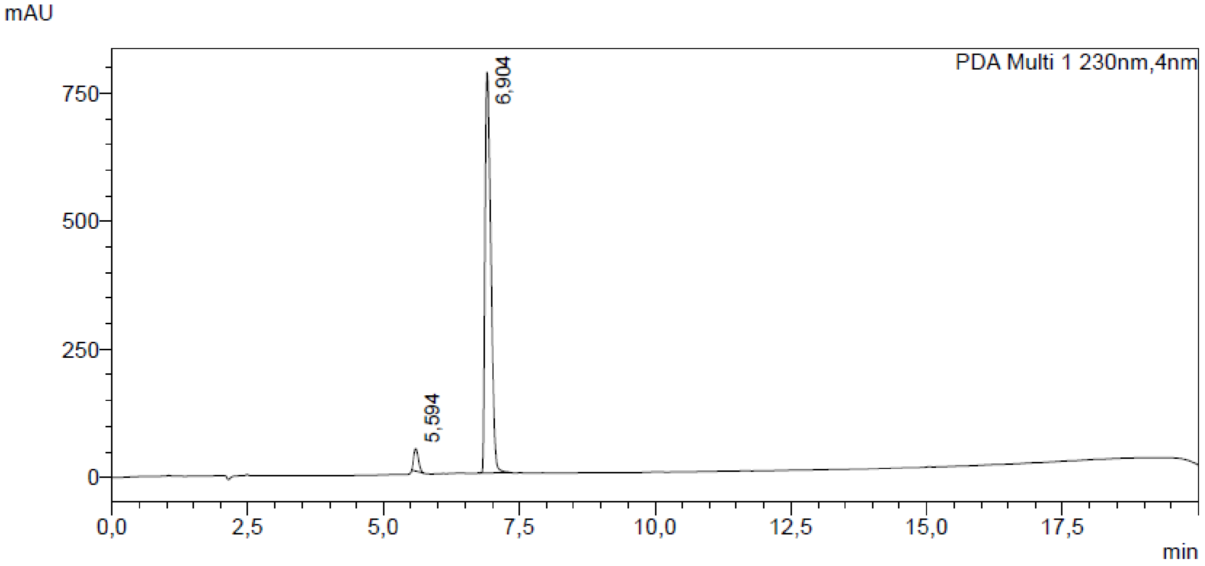

<Peak Table>

PDA Ch1 230nm

Peak#	Ret. Time	Area	Height	Conc.	Unit	Mark	Name
1	5,594	259907	43972	4,266			
2	6,904	5832530	783479	95,734			
Total		6092437	827451				

Analysis Name	av cs96_lys-b.d	Acquisition Date	11/20/19 11:49:59	Operator	Walter Panzeri
Sample Name		Method	Copy of _01tmix_posneg lm.MS	Instrument	esquire3000plus
Comment	1mg/ml dil 1:100 H2O Richiedente: Sgorbati				

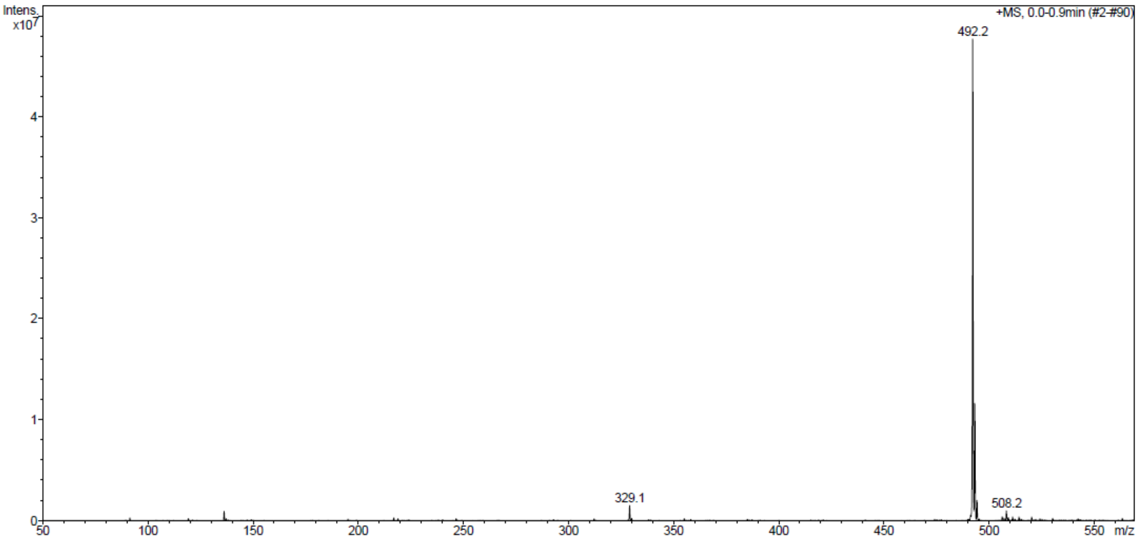

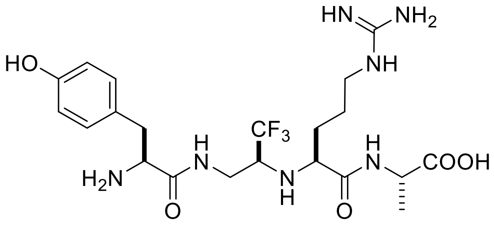

9

HPLC (Shimadzu C18, gradient A:B from 90/10 to 0/100, flow rate 1mL/min, l = 230 nm and 280 nm) tR: 7.081.

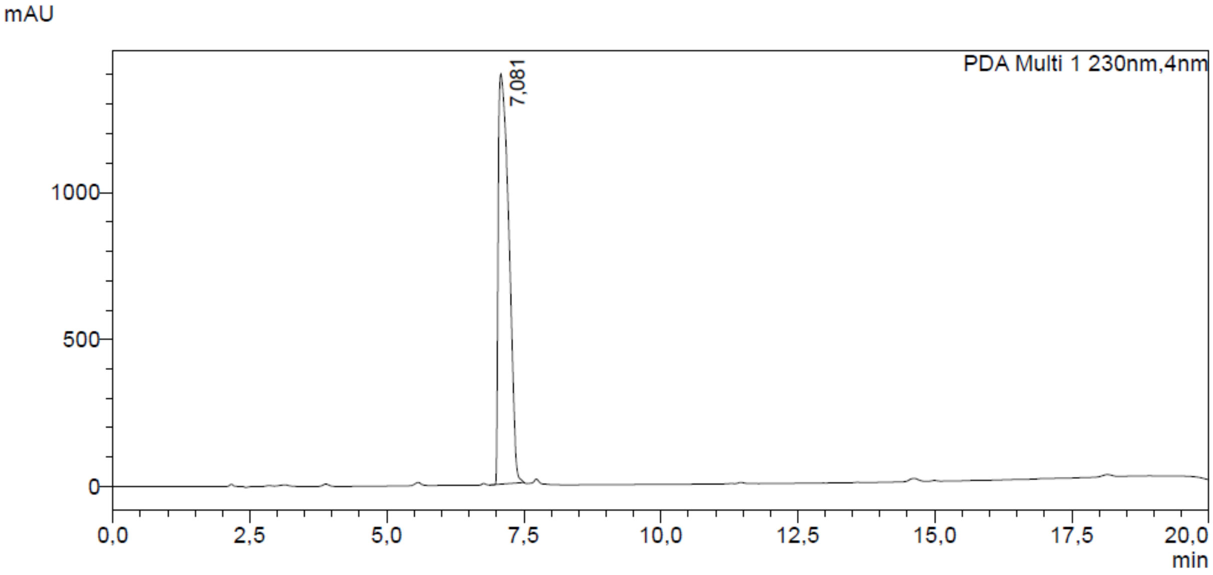

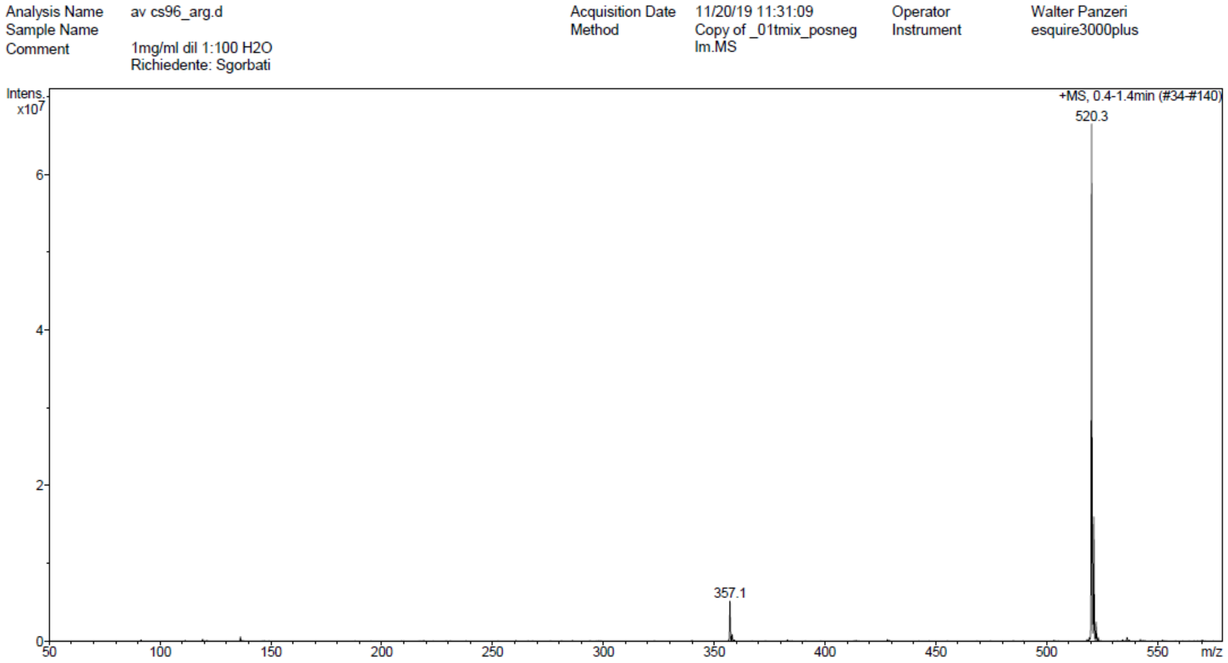

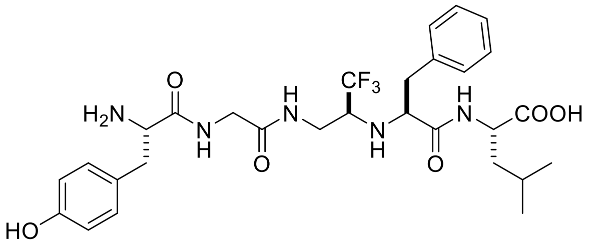

10

HPLC (Shimadzu C18, gradient A:B from 90/10 to 0/100, flow rate 1mL/min, l = 230 nm and 280 nm) tR: 10.043.

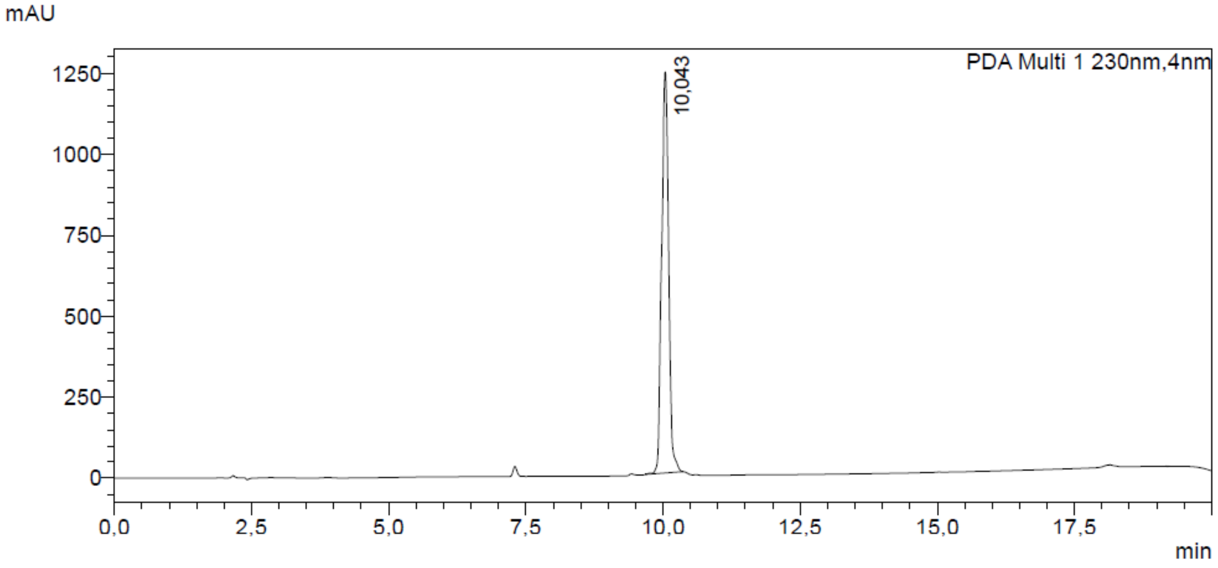

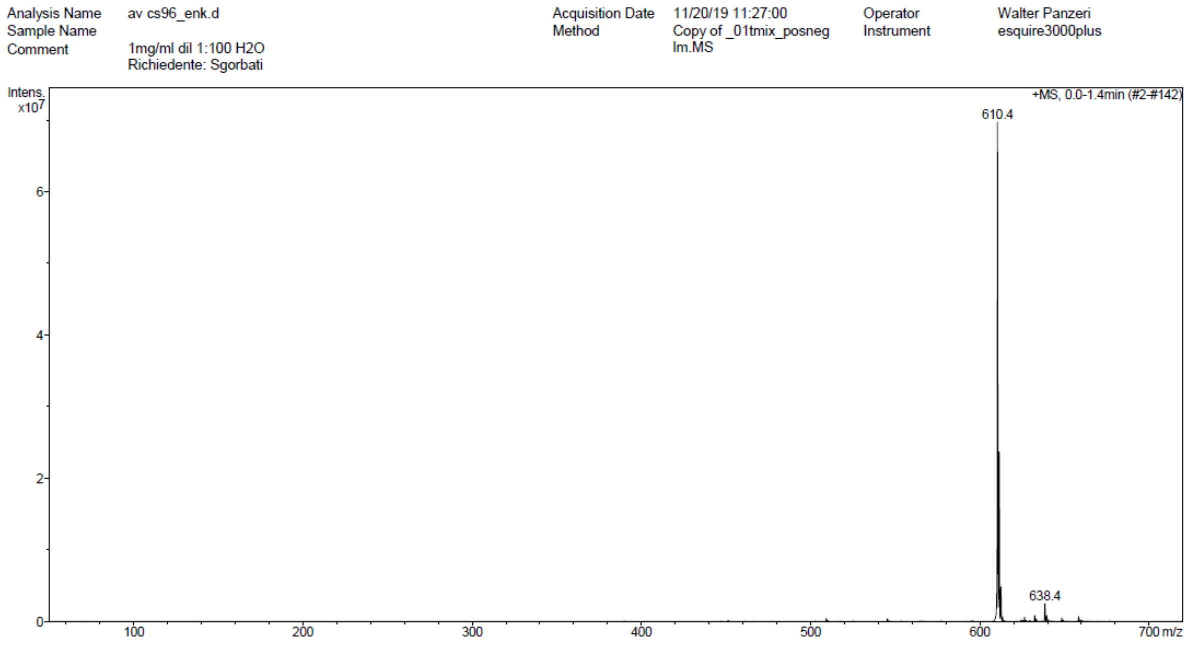

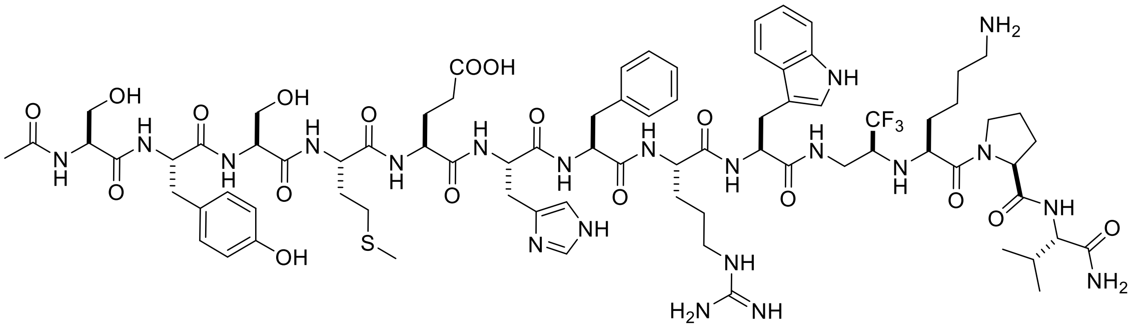

11

HPLC (Shimadzu C18, gradient A:B from 10/90 to 0/100, flow rate 1mL/min, l = 230 nm and 280 nm) tR: 9.663 (major), 10.082 (minor), 11.224 (minor).

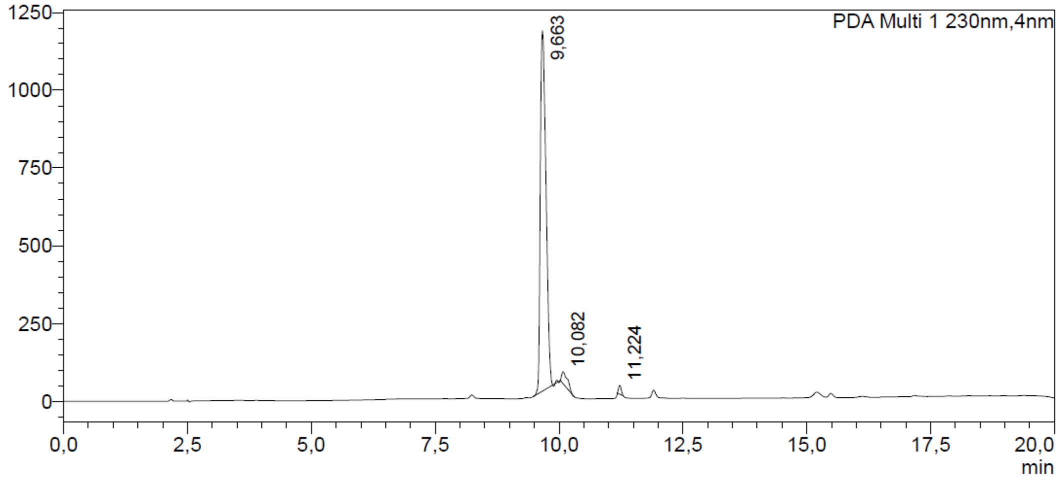

<Peak Table>

PDA Ch1 230nm

Peak#	Ret. Time	Area	Height	Conc.	Unit	Mark	Name
1	9,663	9267912	1159034	95,460			
2	10,082	331058	38908	3,410			
3	11,224	109731	28798	1,130			
Total		9708701	1226740				

HPLC (Shimadzu C18, gradient A:B from 10/90 to 0/100, flow rate 1mL/min, l = 230 nm and 280 nm) tR: 9.721 – Second purification.

<Chromatogram>

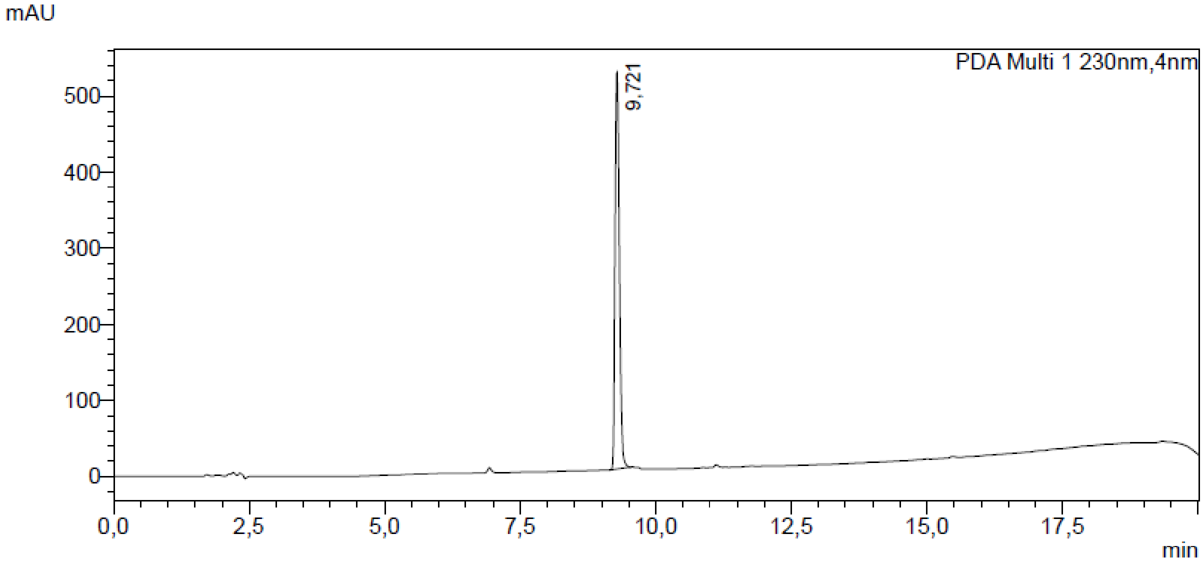

<Peak Table>

PDA Ch1 230nm

Peak#	Ret. Time	Area	Height	Conc.	Unit	Mark	Name
1	9,721	2835616	519563	100,000			
Total		2835616	519563				

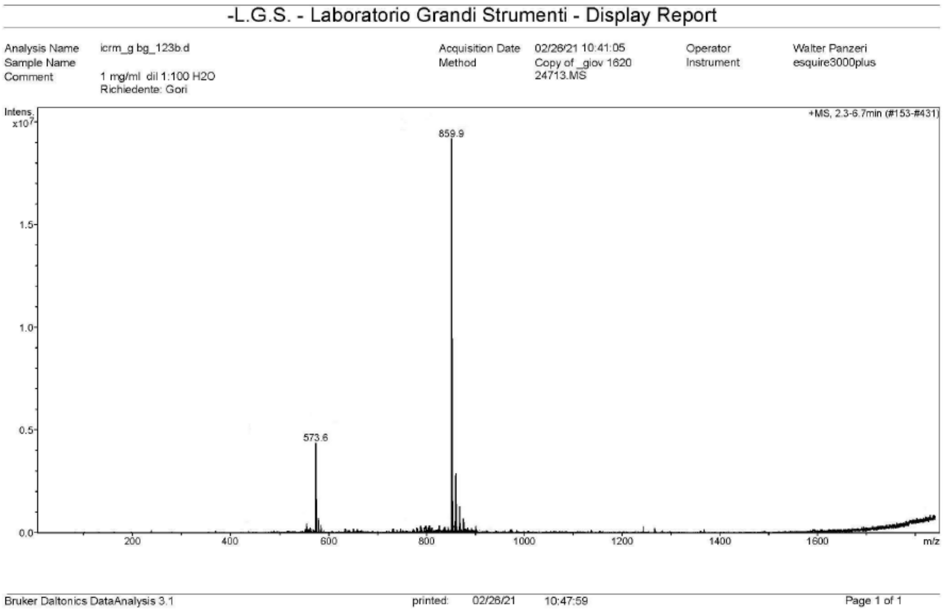

Supplement: Supplementary file 1 — jo1c00853_si_001.pdf [file jo1c00853_si_001.pdf]
